# Titanium‐Catalyzed Intermolecular Hydrothiomethylation of Alkenes

**DOI:** 10.1002/chem.202503427

**Published:** 2025-11-28

**Authors:** Hermann Thye, Felix Fornfeist, Levi L. Schlüschen, Sven Doye

**Affiliations:** ^1^ Institut für Chemie Carl von Ossietzky Universität Oldenburg Oldenburg Germany

**Keywords:** alkenes, C−H activation, catalysis, sulfides, titanium

## Abstract

A catalyst screening for six different α‐alkylation reactions of methyl sulfides with unsaturated substrates performed in hermetically sealable and inexpensive 42‐well aluminum reactor blocks is described. As a result, a new titanium‐catalyzed hydrothiomethylation reaction of alkenes, which takes place under C─H bond activation at the α‐carbon atom of simple methyl sulfides, is presented. The best catalyst is prepared in situ from the readily available titanium catalyst precursor Ti(CH_2_SiMe_3_)_4_, a formamidinato ligand precursor, and the Lewis acid [Ph_3_C][B(C_6_F_5_)_4_]. Although selected 1,2‐disubstituted alkenes, for example, cyclohexene and cyclopentene, undergo successful hydrothiomethylation reactions, the best results are obtained with monosubstituted 1‐alkenes. In these cases, the reactions take place with excellent regioselectivity and give branched hydrothiomethylation products exclusively (23 examples). When dimethyl sulfide is used as a substrate, either the monohydrothiomethylation product or the dihydrothiomethylation product can be obtained with very good selectivity.

## Graphical abstract

Hermetically sealable, inexpensive 42‐well aluminum reactor blocks are used to explore new reactivities of air‐ and moisture‐sensitive titanium catalysts. As a result, methyl sulfides undergo efficient α‐alkylation with alkenes in the presence of a titanium catalyst precursor, a formamidinato ligand, and a Lewis acid, yielding branched hydrothiomethylation products with excellent regioselectivity. Graphical design by Melissa Schlüschen.

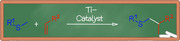

## 1 Introduction

**SCHEME 1:**
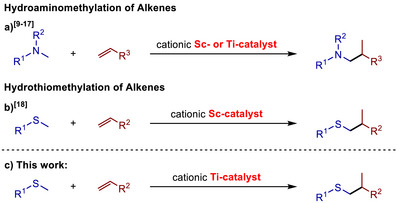
α‐Alkylation reactions of tertiary amines or thioethers with alkenes catalyzed by cationic scandium or titanium complexes.

Transition metal‐catalyzed addition reactions of C─H bonds across π‐bonds of alkenes or similarly unsaturated substrates offer an exciting possibility to form new C─C bonds in a completely atom‐efficient and by‐product‐free manner.[1, 2] In this context, hydroaminoalkylation reactions (Scheme 1a), which take place under C─H bond activation at the α‐carbon atom of simple amines have extensively been studied in recent years and as a result, corresponding reactions of secondary amines can be achieved in the presence of a large number of catalysts based on various transition metals.[3, 4, 5, 6, 7, 8] In contrast, transition metal‐catalyzed hydroaminoalkylation reactions employing simple tertiary amines that do not contain an additional metal‐binding directing group are relatively rare, although corresponding reactions can be achieved with cationic scandium [9, 10, 11, 12, 13, 14] or titanium [15, 16, 17] catalysts (Scheme 1a). In addition to the latter hydroaminoalkylation reactions, closely related hydrothiomethylation reactions of alkenes with simple methyl sulfides using cationic scandium catalysts have also been reported by the Hou‐group (Scheme 1b).[18] The latter reaction deserves particular attention because thioether moieties are found in a wide variety of natural products, agrochemicals, or pharmaceutical products [19, 20] and correspondingly, the development of efficient synthetic methods for the formation and functionalization of thioethers is of great importance. Inspired by the fact that nontoxic titanium, the second most abundant transition metal in the earth´s crust,[21] is significantly less expensive than scandium and by the similar behavior of cationic scandium and titanium catalysts in hydroaminoalkylation chemistry, we recently decided to investigate whether hydrothioalkylation reactions of unsaturated substrates can be achieved in the presence of readily available titanium catalysts (Scheme 1c).

## 2 Results and Discussion

**FIGURE 1:**
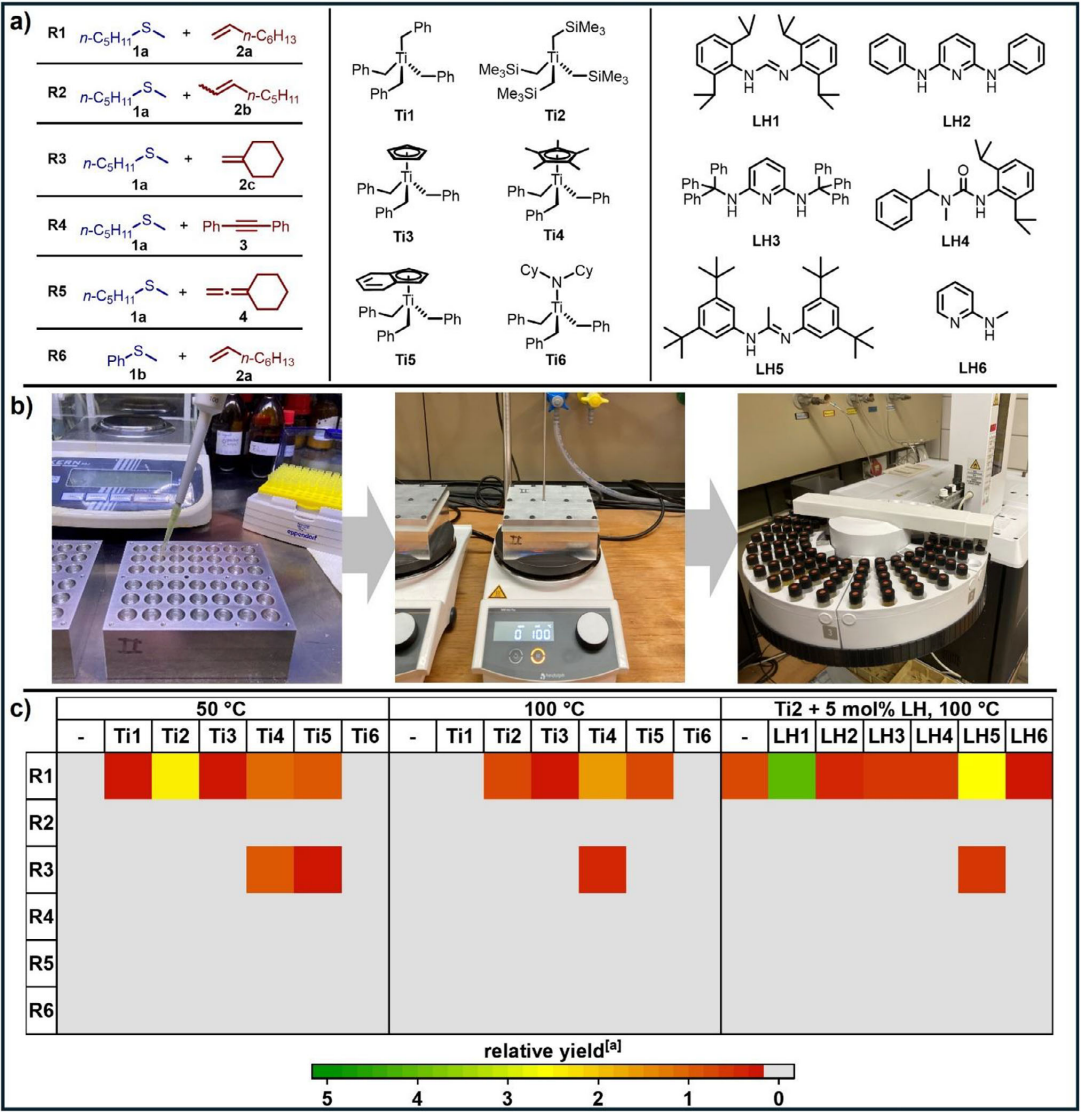
a) Substrate combinations **R1–R6**, titanium complexes **Ti1–Ti6**, and ligand precursors **LH1**‐**LH6** investigated in the initial screening study. b) Screening procedure using in‐house manufactured 42‐well aluminum reactor blocks in combination with subsequent GC analysis. c) Heatmap representation of the screening results. [a] Relative yields refer to the ratios of the GC integrals of the putative product and *p*‐cymene (internal standard). A relative yield of 0 (no reaction) is shown in gray for better visibility. For experimental details, see the Supporting Information.

Based on our experience with studies on the titanium‐catalyzed α‐alkylation of tertiary amines with alkenes,[15, 16] alkynes,[16, 17] and allenes,[16] we chose six reactions involving two different methyl sulfides (**1a**, **1b**, Figure 1a), five unsaturated substrates (**2a–c**, **3**, **4**), and six readily available titanium complexes (**Ti1–Ti6**) for an initial catalyst screening. The screening reactions were carried out on a 50 µmol scale at 50 °C or 100 °C for 24 h in toluene in the presence of *p*‐cymene as an internal standard, 10 mol% of one of the air and moisture sensitive titanium complexes **Ti1–Ti6**, and 10 mol% of the Lewis acid [Ph_3_C][B(C_6_F_5_)_4_] which is essential for the formation of a catalytically active cationic titanium species.[15, 16, 17] To speed up the screening, we used in‐house manufactured, hermetically sealable, inexpensive 42‐well aluminum reactor blocks (Figure 1b, 32€ per block) designed to accommodate standard 1.5 mL screw‐capped glass vials suitable for automated GC analysis. Using Eppendorf pipettes, the vials could easily be charged with previously prepared stock solutions of the various components of the reaction mixtures inside a nitrogen‐filled glovebox (For details, see the Supporting Information). The blocks were then closed with composite flat seals (2 mm PTFE and 3 mm silicone) and an aluminum lid and removed from the glovebox. After heating the reactor blocks on a regular heating plate for 24 h to 50 °C or 100 °C, the vials were simply filled with dichloromethane and transferred to the autosampler of an FID‐equipped gas chromatograph. The results of the GC analysis are presented in Figure 1c; the relative yield for each catalyst‐reaction‐combination refers to the ratio of the GC integrals of the putative product and the internal standard *p*‐cymene.

**SCHEME 2:**
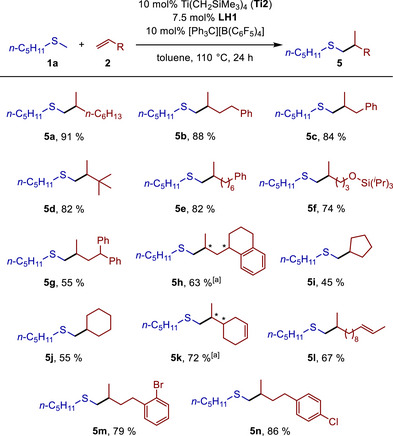
Scope of the titanium‐catalyzed hydrothiomethylation of alkenes with methyl pentyl sulfide (**1a**). Reaction conditions: methyl pentyl sulfide (**1a**, 1.0 mmol), alkene (**2**, 1.5 mmol), Ti(CH_2_SiMe_3_)_4_ (**Ti2**, 0.1 mmol, 10 mol%), **LH1** (0.075 mmol, 7.5 mol%), [Ph_3_C][B(C_6_F_5_)_4_] (0.1 mmol, 10 mol%), toluene (1.0 mL), Schlenk tube (*V* = 5 mL), 110°C, 24 h, yields after purification. [a] Two diastereomers in a ratio of approximately 1:1, stereogenic centers marked with *.

As can be seen from the results of the screenings performed at 50 °C or 100 °C (Figure 1c), none of the titanium complexes **Ti1–Ti6** could be used for successful hydrothiomethylation reactions involving an alkyne [diphenylacetylene (**3**); reaction **R4**], an allene [vinylidenecyclohexane (**4**); reaction **R5**], an unstrained internal alkene [*E*/*Z*‐2‐octene (**2b**); reaction **R2**], or an aryl‐substituted methyl sulfide [thioanisole (**1b**); reaction **R6**]. In contrast, the reactions of methyl pentyl sulfide (**1a**) with 1‐octene (**2a**; reaction **R1**) or methylenecyclohexane (**2c**; reaction **R3**) turned out to be more promising because in both cases, at least trace amounts of putative hydrothiomethylation products could be detected by GC and subsequent GC/MS analysis. While at 50 °C, titanium complex **Ti2** gave by far the best result for reaction **R1**, it was found that at 100 °C increased amounts of various by‐products are generally formed. Although we could not determine the exact structures of the by‐products formed in reaction **R1**, GC/MS analysis suggests that, for example, two α‐alkylation reactions of methyl pentyl sulfide (**1a**) take place at this temperature. To improve the selectivity of the reactions, we then carried out another screening using homoleptic titanium complex **Ti2** in combination with 5 mol% of one of the ligand precursors **LH1**‐**LH6**. It should be emphasized that **LH1**‐**LH6** are ligand precursors commonly used in titanium‐catalyzed hydroaminoalkylation reactions.[3, 4, 5, 6, 7, 8, 15, 16, 17] Fortunately, among these ligand precursors, formamidine **LH1**[22] accelerated reaction **R1**, leading to good conversion of the starting materials after 24 h at 100 °C, and significantly suppressed the formation of undesired by‐products. The product of the reaction between methyl pentyl sulfide (**1a**) and 1‐octene (**2a**, reaction **R1**), which was subsequently isolated from a corresponding experiment on a 1 mmol scale, was then identified as the branched hydrothiomethylation product **5a** (Scheme 2). Unfortunately, all corresponding attempts to isolate a hydrothiomethylation product from the reaction of methylenecyclohexane (**2c**) with methyl pentyl sulfide (**1a**, reaction **R3**) failed due to insufficient conversion of the starting materials, and therefore, we decided to focus exclusively on further optimization of reaction **R1**. For that purpose, we carried out additional screening reactions at 70–130 °C (Figure S4), with ligand precursor loadings of 0–15 mol% (Figure S5), and reaction times of 1–72 h (Figure S6) which led to the conclusion that reaction **R1** is best carried out at 110 °C for 24 h in the presence of 10 mol% **Ti2**, 7.5 mol% **LH1**, and 10 mol% [Ph_3_C][B(C_6_F_5_)_4_] (For details, see the Supporting Information). In this context, it should be emphasized that a larger amount of **LH1** (10.0 or 12.5 mol%) did not lead to significantly improved results that would justify the use of a larger amount of **LH1** (Figure S5).

Under the optimized conditions, we then repeated a 1 mmol scale reaction between methyl pentyl sulfide (**1a**) and 1‐octene (**2a**), which gave access to the branched hydrothiomethylation product **5a** in 91% yield after purification (Scheme 2). It is worth mentioning that no regioisomeric linear hydrothiomethylation product was detected in any of our experiments, indicating excellent regioselectivity of the investigated transformation. As can be seen from Scheme 2, numerous other alkenes could also be reacted successfully with sulfide **1a**, giving access to the hydrothiomethylation products **5b–n** in moderate to very good yields (45%–88%). In case of monosubstituted alkenes, only the branched hydrothiomethylation products (**5a–h**, **5k–n**) were formed. Surprisingly and in sharp contrast to the screening results for reaction **R2**, where *E*/*Z*‐2‐octene (**2b**) was used as an example of an unstrained internal alkene, cyclopentene and cyclohexene underwent successful hydrothiomethylation to give the desired products **5i** and **5j** in 45% and 55% yield, respectively. However, the fact that we did not observe any reactivity of 3‐hexene is in good agreement with the screening results. The relatively low yields of **5i** and **5j** can easily be explained by a competing polymerization of the alkene starting materials cyclopentene and cyclohexene, which we observed under the reaction conditions. In corresponding attempts to convert norbornene or styrene, alkene polymerization completely dominated, and consequently, no hydrothiomethylation products could be obtained from these substrates. While 1,1‐disubstituted alkenes such as methylenecyclohexane and methylenecyclopentane also showed no reactivity, sterically demanding 3,3‐dimethyl‐1‐butene reacted smoothly to give product **5d** in 82% yield. As demonstrated by the products **5m** and **5n**, which were isolated in 79% and 86% yield, respectively, the new reaction tolerates bromo and chloro substitution, offering the possibility of further functionalization, for example, by cross‐coupling reactions.[23] With regard to heteroatom substitution, product **5f** (74% yield) additionally demonstrates that silyl‐protected alcohols are also suitable substrates for hydrothiomethylation reactions. By exploiting the already established fact that monosubstituted terminal double bonds are significantly more reactive than internal double bonds, we were also able to convert 4‐vinyl cyclohexene and (*E*)‐trideca‐1,11‐diene selectively into the branched products **5k** and **5l** in yields of 72% and 67%, respectively. In both cases, no reaction of the internal double bond occurred. Finally, it should be noted that reactions carried out with alkene substrates containing a chiral center did not show any diastereoselectivity. In these cases, the products **5** **h** and **5k** were obtained as mixtures of two diastereomers in a ratio of approximately 1:1 (according to GC and ^13^C NMR analysis).

**SCHEME 3:**
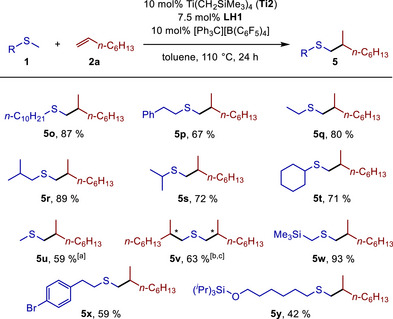
Scope of the titanium‐catalyzed hydrothiomethylation of 1‐octene (**2a**) with sulfides. Reaction conditions: sulfide (**1**, 1.0 mmol), 1‐octene (**2a**, 1.5 mmol), Ti(CH_2_SiMe_3_)_4_ (**Ti2**, 0.1 mmol, 10 mol%), [Ph_3_C][B(C_6_F_5_)_4_] (0.1 mmol, 10 mol%), toluene (1.0 mL), Schlenk tube (*V* = 5 mL), 110°C, 24 h, yields after purification. [a] Dimethyl sulfide (1.5 mmol), 1‐octene (**2a**, 1.0 mmol). [b] Dimethyl sulfide (1.0 mmol), 1‐octene (**2a**, 3.0 mmol). [c] Two diastereomers in a ratio of approximately 1:1, stereogenic centers marked with *.

Next, we turned our attention toward reactions of additional sulfides with 1‐octene (**2a**) and found that various methyl sulfides undergo smooth α‐alkylation at the methyl group under the already established reaction conditions (Scheme 3). In the case of unfunctionalized methyl sulfides, the desired branched hydrothiomethylation products (**5o**‐**t**) could be isolated in good to very good yields (67%–89%) and, as shown by the successful reactions of the sterically demanding substrates cyclohexyl methyl sulfide and isopropyl methyl sulfide, steric hindrance is tolerated to a certain degree. On the other hand, *tert*‐butyl methyl sulfide, adamantyl methyl sulfide, and methyl trityl sulfide did not show any reactivity. The results obtained with the simplest sulfide, dimethyl sulfide, are particularly promising because in this case, either the monohydrothiomethylation product **5u** or the dihydrothiomethylation product **5v** could be obtained with very good selectivity. As suggested by the stoichiometry, the use of 1.5 equivalents of dimethyl sulfide and 1.0 equivalent of 1‐octene (**2a**) favored the formation of the monohydrothioalkylation product **5u**, which could be isolated in 59% yield. In contrast, the use of a threefold excess of 1‐octene (**2a**) favored the dialkylation of dimethyl sulfide and provided access to the dihydrothioalkylation product **5v** in 63% yield. In the latter case, two diastereomers were obtained in a ratio of approximately 1:1. The fact that the dialkylation of dimethyl sulfide selectively occurs at the two methyl groups strongly underlines the fact that the catalytic system is not reactive enough to achieve alkylation reactions at α‐methylene groups of sulfides; for example, diethyl sulfide, tetrahydrothiophene, and tetrahydro‐2*H*‐thiopyran were found to be unreactive under the reaction conditions.[24] With regard to heteroatom substitution, products **5w**‐**y** (42%–93% yield) demonstrate that bromo‐ and trimethylsilyl‐substituted sulfides react successfully and even the presence of a triisopropylsilyl ether is tolerated.

**SCHEME 4:**
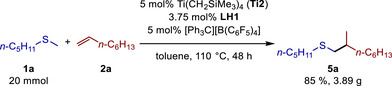
Multigram scale hydrothiomethylation reaction of 1‐octene (**2a**) with methyl pentyl sulfide (**1a**).

To demonstrate the robustness of our catalytic system, we also attempted to reduce the catalyst loading to 5 mol% **Ti2** (3.75 mol% **LH1** accordingly) and to perform the reaction between methyl pentyl sulfide (**1a**) and 1‐octene (**2a**) on a 20 mmol scale (Scheme 4). For this purpose, we increased the reaction time to 48 h, after which the desired alkylated sulfide **5a** could be isolated in 85% yield (3.89 g), a result that compares well with the reaction on the 1 mmol scale (91%).

**SCHEME 5:**
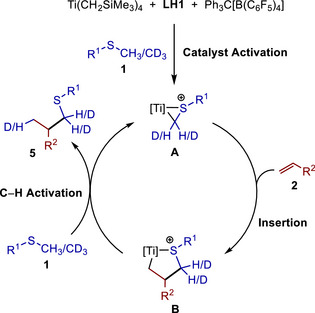
Proposed plausible catalytic cycle of the titanium‐catalyzed hydrothiomethylation of alkenes.

**SCHEME 6:**
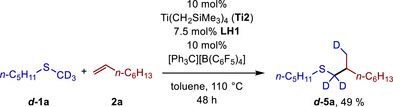
Titanium catalyzed hydrothiomethylation of 1‐octene (**2a**) with methyl‐d3 pentyl sulfide (***d*‐1a**). Reaction conditions: methyl‐d3 pentyl sulfide (***d*‐1a**, 1.0 mmol), 1‐octene (**2a**, 1.5 mmol), Ti(CH_2_SiMe_3_)_4_ (**Ti2**, 0.1 mmol, 10 mol%), **LH1** (0.075 mmol, 7.5 mol%), [Ph_3_C][B(C_6_F_5_)_4_] (0.1 mmol, 10 mol%), toluene (1.0 mL), Schlenk tube (*V* = 5 mL), 110°C, 48 h, yield after purification.

In analogy to mechanistic studies on the α‐alkylation of tertiary amines or sulfides with alkenes in the presence of cationic Sc catalysts,[9, 10, 11, 12, 13, 14, 18, 25] it is assumed that a cationic titanathiirane (**A**, Scheme 5) acts as the catalytically active species. Although the exact structure of **A** remains unclear, C─C bond‐forming, regioselective insertion of the alkene **2** into the Ti─C bond of **A** would deliver titanatetrahydrothiophene **B**, from which **A** could be regenerated by C─H bond activation of the methyl sulfide **1**. The orientation of the R^2^‐substituent of the alkene toward the sterically less demanding methylene group of the titanathiirane **A** during the insertion makes the observed regioselectivity of the hydrothiomethylation easily understandable. In good agreement with this proposed catalytic cycle is the fact that a reaction of 1‐octene (**2a**) with the deuterated sulfide methyl‐d3 pentyl sulfide (***d*‐1a**, Scheme 6) selectively delivers product ***d*‐5a**, which exhibits monodeuteration of the methyl group. During this reaction, we also observed that the deuterated sulfide ***d*‐1a** reacts approximately four times more slowly than **1a** and therefore, the reaction time had to be extended to 48 h to isolate the hydrothiomethylation product ***d*‐5a** in 49% yield. This strong kinetic isotope effect, which was also confirmed for the formation of **5p**/***d*‐5p** (Figure S7) (For details, see the Supporting Information), suggests that C─H bond activation is the rate‐determining step in the catalytic cycle.

## 3 Conclusion

In summary, we have shown for the first time that hydrothiomethylation reactions of alkenes with methyl sulfides can be achieved in the presence of titanium catalysts. Optimization studies revealed that the best catalyst is prepared in situ from the readily available catalyst precursor Ti(CH_2_SiMe_3_)_4_, the formamidinato ligand precursor **LH1**, and the Lewis acid [Ph_3_C][B(C_6_F_5_)_4_]. Although cyclic internal alkenes undergo successful hydrothiomethylation, best results are obtained with terminal alkenes. In these cases, the reactions proceed with excellent regioselectivity and give exclusively branched hydrothiomethylation products. In addition, it was demonstrated that screening reactions with highly air‐ and moisture‐sensitive titanium complexes can be easily and reliably performed in inexpensive 42‐well aluminum reactor blocks designed to accommodate standard 1.5 mL screw‐capped glass vials that are suitable for subsequent automated GC analysis. Further optimization studies of the new reaction, as well as detailed mechanistic investigations, are underway in our laboratories.

## Conflicts of Interest

The authors declare no conflict of interest.

## Supporting information




**Supporting Information File 1**: chem70499‐sup‐0001‐SuppMat.pdf.

## Data Availability

The data that support the findings of this study are available in the supplementary material of this article.
