# Supplementary material for: Titanium‐Catalyzed Intermolecular Hydrothiomethylation of Alkenes
Source: Chemistry. 2025 Nov 28;32(1):e03427. doi: 10.1002/chem.202503427 (PMC12759164; doi:10.1002/chem.202503427)
Supplement: Supplementary file 1 — Supporting Information File 1: chem70499‐sup‐0001‐SuppMat.pdf. [file CHEM-32-e03427-s001.pdf]

Table of Contents

1.	General Information	2
2.	Screening Reactions	3
3.	Synthesis of Tetrakis[(trimethylsilyl)methyl]titanium (**Ti2**)	8
4.	Catalytic Reactions	9
5.	Mechanistic Investigations	21
6.	NMR Spectra	23
7.	References	51

1. General Information

All reactions were performed under an inert atmosphere of nitrogen or argon in oven-dried glassware using standard glovebox or Schlenk line techniques. Toluene, Et_2O and *n*-hexane were distilled from sodium wire and degassed prior to use. All substrates were dried, distilled and degassed (freeze-pump-thaw) or recrystallized before being introduced into the glovebox (Vigor, Sci-Lab). Sulfides were either purchased from commercial sources or prepared from the corresponding thiols and methyl iodide according to a literature procedure.[1] $[Ph_3C][B(C_6F_5)_4]$ was purchased from BLD Pharmatech GmbH and used as received. *n*-Pentane and CH_2Cl_2 used for chromatography were distilled prior to use. Silica gel from Grace (particle size = 40-63 μm) was used for chromatography. Silica gel 60 sheets with fluorescent indicator (254 nm) from Macherey-Nagel™ were used for thin layer chromatography; substances were detected with UV light or a H_2PtCl_6/KI solution (3 g KI and 250 mg $H_2PtCl_6 \cdot 6\ H_2O$ in 250 mL H_2O)[2] used as a spray reagent (sulfides typically give light yellow spots on pink background). The yields given in Section 4 (Catalytic Reactions) refer to the yield of a single experiment. Products that have already been reported in the literature were identified by ^1H NMR and ^{13}C NMR spectroscopy; all analytical data was found to be consistent with the literature. New substances were additionally characterized by infrared spectroscopy (IR) and high-resolution mass spectrometry (HRMS). NMR spectra were recorded on a JEOL JNM-ECZL or Bruker Avance III 500 MHz spectrometer at a temperature of 305 K. ^1H NMR spectra are referenced to the residue solvent signals (δ ^1H = 7.26 ppm for $CDCl_3$, δ ^1H = 7.16 ppm for C_6D_6, δ ^1H = 2.05 ppm for $(CD_3)_2CO$ or δ ^1H = 2.09 ppm for toluene-d8). ^{13}C NMR spectra are referenced to the central line of the solvent signal (δ $^{13}C\{^1H\}$ = 77.16 ppm for $CDCl_3$, δ $^{13}C\{^1H\}$ = 128.06 ppm for C_6D_6, δ $^{13}C\{^1H\}$ = 29.84 ppm for $(CD_3)_2CO$ or δ $^{13}C\{^1H\}$ = 20.40 ppm for toluene-d8). ^{29}Si NMR spectra were calibrated against an external standard [δ $^{29}Si(Me_2SiHCl)$ = 11.1 ppm in relation to $SiMe_4$ (δ = 0.0 ppm)]. Infrared spectra were recorded on a Shimadzu IRSpirit QATR-S spectrometer. HRMS analyses were performed on a Thermo Scientific DFS (EI, 70 eV) or Thermo Scientific Orbitrap Exploris 240 (ESI+) spectrometer. GC analyses were performed on a Shimadzu GC-2030 gas chromatograph (column: FS-SE-54-CB-0.25, length = 30 m, inner diameter = 0.32 mm, film thickness = 0.25 μm, (94%-methyl)-(5%-phenyl)-(1%-vinyl)polysiloxane) with a flame ionization detector.

2. Screening Reactions

Catalyst and Ligand Precursor Screenings

Stock Solution Preparation

For a typical catalyst screening, stock solutions of the reactants, titanium complexes, ligand precursors (Figure S1), and the Lewis acid $[Ph_3C][B(C_6F_5)_4]$ had to be prepared first. The entire process, until the heating of the reactor block, was performed inside of a Vigor Sci-Lab glovebox under nitrogen atmosphere. Only thoroughly dried and degassed reactants and solvents were used. For the reactant stock solutions, the corresponding sulfide (**1**, 1.250 mmol) and an unsaturated substrate (**2-4**, 1.875 mmol) were both weighed into the same 1 mL volumetric flask, and the volume was brought up to 1 mL with toluene. The titanium complex and ligand precursor stock solutions were prepared in the same manner, by weighing in the corresponding complex (**Ti1**-**Ti6**, 0.125 mmol) or the ligand precursor (**LH1-LH6**, 0.03125 mmol) into a 1 mL volumetric flask and bringing the volume up to 1 mL with toluene. For the Lewis acid stock solution, $[Ph_3C][B(C_6F_5)_4]$ (0.5 mmol) and the internal standard *p*-cymene (1 mmol) were weighed into a 10 mL volumetric flask before filling the flask with toluene. The different concentrations of the solutions were chosen to ensure solubility of all substances while keeping the overall reaction volume as small as possible.

Reaction Setup

For the screening experiments, in-house manufactured, hermetically sealable, inexpensive 42-well aluminum reactor blocks (116×105×40 mm, 32 € per block, Figure S2) were used. The wells were designed to take up standard 1.5 mL screw neck glass vials used for GC or HPLC analysis (available from Macherey-Nagel™). The reactor blocks were brought into the glovebox and then the glass vials were filled with aliquots of the prepared stock solutions using Eppendorf pipettes. In the case of catalyst screenings without additional ligand precursors, each vial was filled with 40 µL of the corresponding reactant solution (50 µmol sulfide **1**, 1.0 equiv and 75 µmol unsaturated substrate **2-4**, 1.5 equiv), 40 µL of the titanium complex stock solution (5 µmol **Ti1**-**Ti6**, 10 mol%,) in a pattern visualized in Figure S3. Then 100 µL of the Lewis acid solution (5 µmol $[Ph_3C][B(C_6F_5)_4]$, 10 mol% and 10 µmol *p*-cymene, 20 mol%) were also added to every reaction mixture. It should be emphasized that the Lewis acid containing stock solution was continuously stirred to ensure sufficient homogenization. When additional ligand precursors were used, 80 µL of the corresponding stock solution (2.5 µmol **LH1-LH6**, 5 mol%, Figure S3) were also added to each vial. After the 42 vials of an aluminum reactor block had been filled, the block was closed with composite flat seals (2 mm PTFE and 3 mm silicone) and the aluminum lid was screwed tight with 12 screws. To ensure even pressure distribution, the screws were fastened in a star pattern. The sealed aluminum reactor block was then brought outside the glove box and heated on a standard magnetic stirring plate (Ø = 125 mm) with a temperature sensor inserted into the block. Preliminary experiments showed that this method provides sufficiently uniform heat distribution for temperatures up to 180 °C. It should be noted that even though stirring with small magnetic bars inside the vials is possible, it usually does not make a difference compared to the unstirred reactions, at least at that scale of the reactions.

Workup and Analysis

After heating the reactor block to the given temperature for 24 h, the block was allowed to slowly cool to room temperature. Rapid cooling with ice proved to be unsuitable, as it causes some of the glass vials to shatter. Afterwards, the reactor block was opened, and the individual vials were removed, quenched with CH_2Cl_2, sealed with a rubber septum screw cap and placed directly into the autosampler of a Shimadzu GC-2030 gas chromatograph with a flame ionization detector. After letting the solid residues of the quenched mixtures settle at the bottom of the vials for a minimum of 4 hours, gas chromatographic analysis of every reaction mixture was performed.

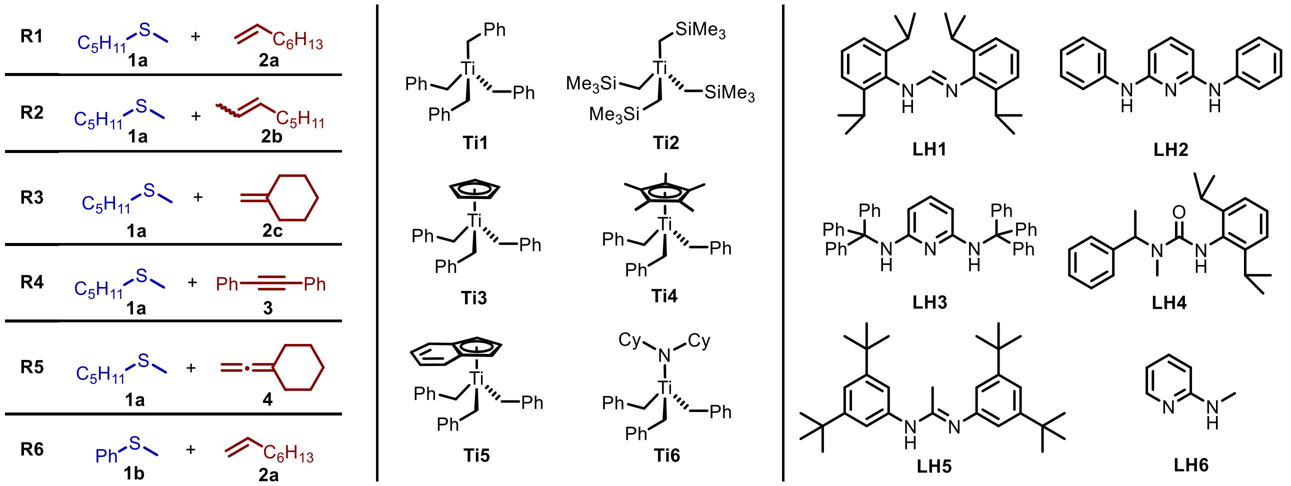

Figure S1: Substrate pairs of reactions **R1-R6**, titanium complexes **Ti1-Ti6**, and ligand precursors **LH1-LH6** investigated.

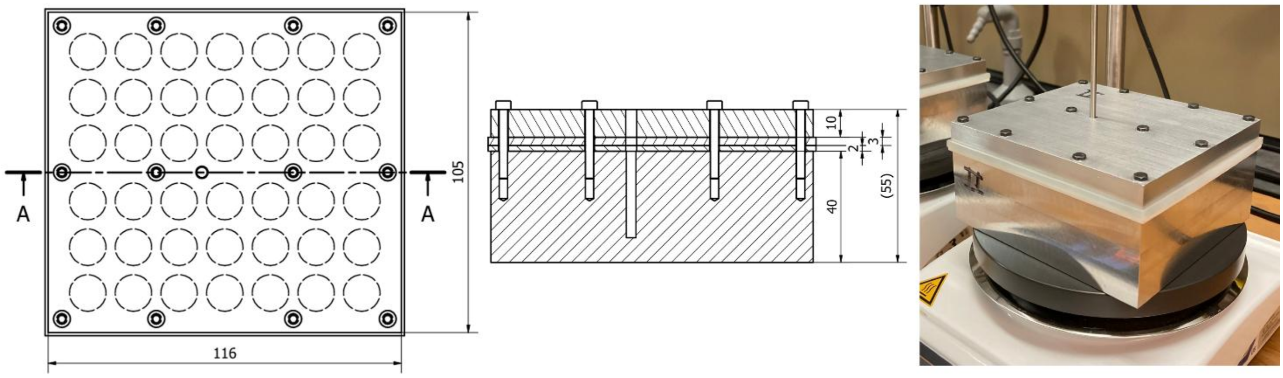

Figure S2: Dimensions (in mm) and view of the assembled 42-well aluminum reactor block.

	50 °C							100 °C							Ti2 + 5 mol% LH, 100 °C						
	-	Ti1	Ti2	Ti3	Ti4	Ti5	Ti6	-	Ti1	Ti2	Ti3	Ti4	Ti5	Ti6	-	LH1	LH2	LH3	LH4	LH5	LH6
R1	0,00	0,28	2,34	0,32	1,02	0,93	0,00	0,00	0,00	0,83	0,31	1,61	0,74	0,00	0,83	4,13	0,45	0,56	0,54	2,52	0,27
R2	0,00	0,00	0,00	0,00	0,00	0,00	0,00	0,00	0,00	0,00	0,00	0,00	0,00	0,00	0,00	0,00	0,00	0,00	0,00	0,00	0,00
R3	0,00	0,00	0,00	0,00	0,88	0,24	0,00	0,00	0,00	0,00	0,00	0,42	0,00	0,00	0,00	0,00	0,00	0,00	0,00	0,64	0,00
R4	0,00	0,00	0,00	0,00	0,00	0,00	0,00	0,00	0,00	0,00	0,00	0,00	0,00	0,00	0,00	0,00	0,00	0,00	0,00	0,00	0,00
R5	0,00	0,00	0,00	0,00	0,00	0,00	0,00	0,00	0,00	0,00	0,00	0,00	0,00	0,00	0,00	0,00	0,00	0,00	0,00	0,00	0,00
R6	0,00	0,00	0,00	0,00	0,00	0,00	0,00	0,00	0,00	0,00	0,00	0,00	0,00	0,00	0,00	0,00	0,00	0,00	0,00	0,00	0,00

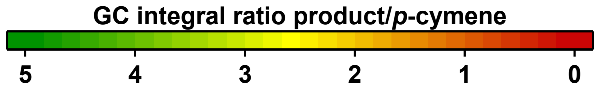

Figure S3: Detailed results of the screening experiments. Integral ratios refer to the ratio of the GC integrals obtained for (assumed) alkylation products and *p*-cymene.

Optimization Reactions 1: Temperature Screening

In a nitrogen filled glovebox, methyl pentyl sulfide (**1a**, 237 mg, 2.0 mmol), 1-octene (**2a**, 337 mg, 3.0 mmol), $Ti(CH_2SiMe_3)_4$ (**Ti2**, 79 mg, 0.2 mmol, 10 mol%), ligand precursor (**LH1**, 37 mg, 0.1 mmol, 5 mol%), and *p*-cymene (54 mg, 0.4 mmol) were weighed into a volumetric flask (5 mL) and the total volume was brought up to 5 mL with toluene to give a stock solution. Individual portions of $[Ph_3C][B(C_6F_5)_4]$ (19 mg, 0.02 mmol, 10 mol%) were weighed into glass vials and an aliquot of the stock solution (500 µL, 0.2 mmol reaction scale) was added. The resulting solutions were then individually transferred into glass ampoules (1 mL) via syringe. After temporarily sealing the ampoules with silicon grease, a propane/oxygen torch was used to seal the ampoules outside of the glovebox.[3] Subsequently, they were heated in an oil bath to the given temperature for 16 h. The reaction mixtures were then cooled to room temperature and transferred into flasks with CH_2Cl_2 (5 mL) before being subjected to GC analysis. Ratios given in Figure S4 refer to the ratios of the integrals obtained for the hydrothiomethylation product **5a** and *p*-cymene.

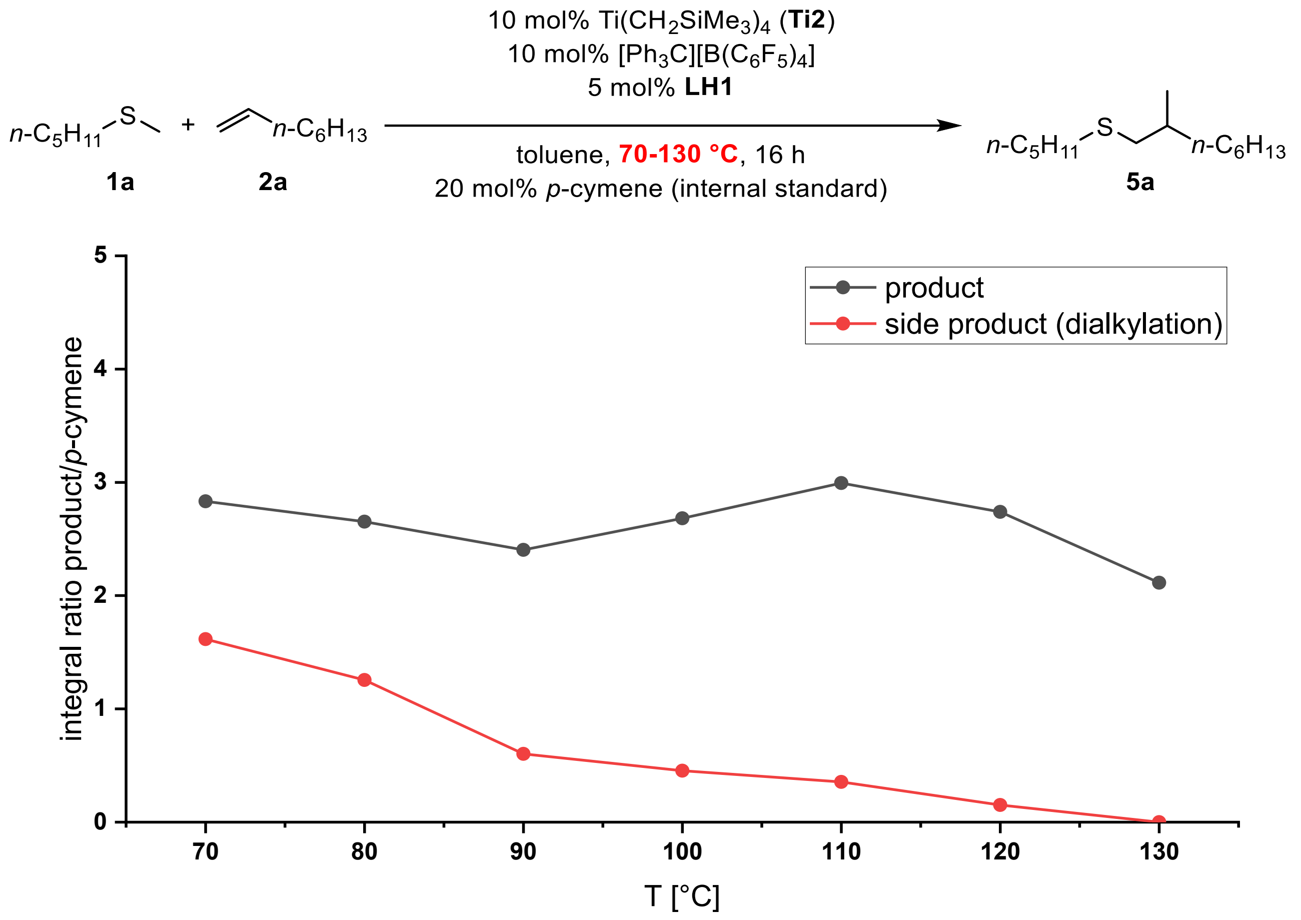

Figure S4: Temperature screening for the alkylation of methyl pentyl sulfide (**1a**) with 1-octene (**2a**) in the presence of complex **Ti2** and ligand precursor **LH1**.

Optimization Reactions 2: Ligand Precursor Loading Screening

In a nitrogen filled glovebox, methyl pentyl sulfide (**1a**, 237 mg, 2.0 mmol), 1-octene (**2a**, 337 mg, 3.0 mmol), $Ti(CH_2SiMe_3)_4$ (**Ti2**, 79 mg, 0.2 mmol, 10 mol%), and *p*-cymene (54 mg, 0.4 mmol) were weighed into a volumetric flask (2 mL) and the total volume was brought up to 2 mL with toluene to give a stock solution. In addition, a separate ligand precursor stock solution was prepared from **LH1** (68 mg, 0.1875 mmol) in a 2 mL volumetric flask filled with toluene. Aliquots of this stock solution corresponding to 0-15 mol% ligand loading (0-320 µL), the reciprocal amount of toluene (320-0 µL), and aliquots of the reactant stock solution (200 µL, 0.2 mmol reaction scale) were mixed with individual portions of $[Ph_3C][B(C_6F_5)_4]$ (19 mg, 0.02 mmol, 10 mol%) in separate glass vials. The resulting solutions were then individually transferred into glass ampoules (1 mL) via syringe. After temporarily sealing the ampoules with silicon grease, a propane/oxygen torch was used to seal the ampoules outside of the glovebox.[3] Subsequently, they were heated in an oil bath to 110 °C for 16 h. The reaction mixtures were then cooled to room temperature and transferred into flasks with CH_2Cl_2 (5 mL) before being subjected to GC analysis. Ratios given in Figure S5 refer to the ratios of the integrals obtained for the hydrothiomethylation product **5a** and *p*-cymene.

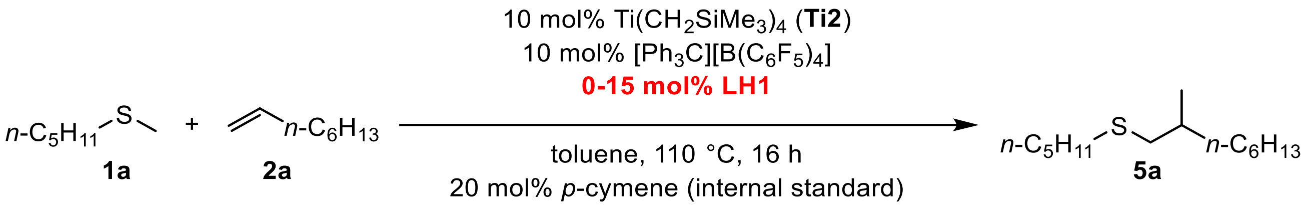

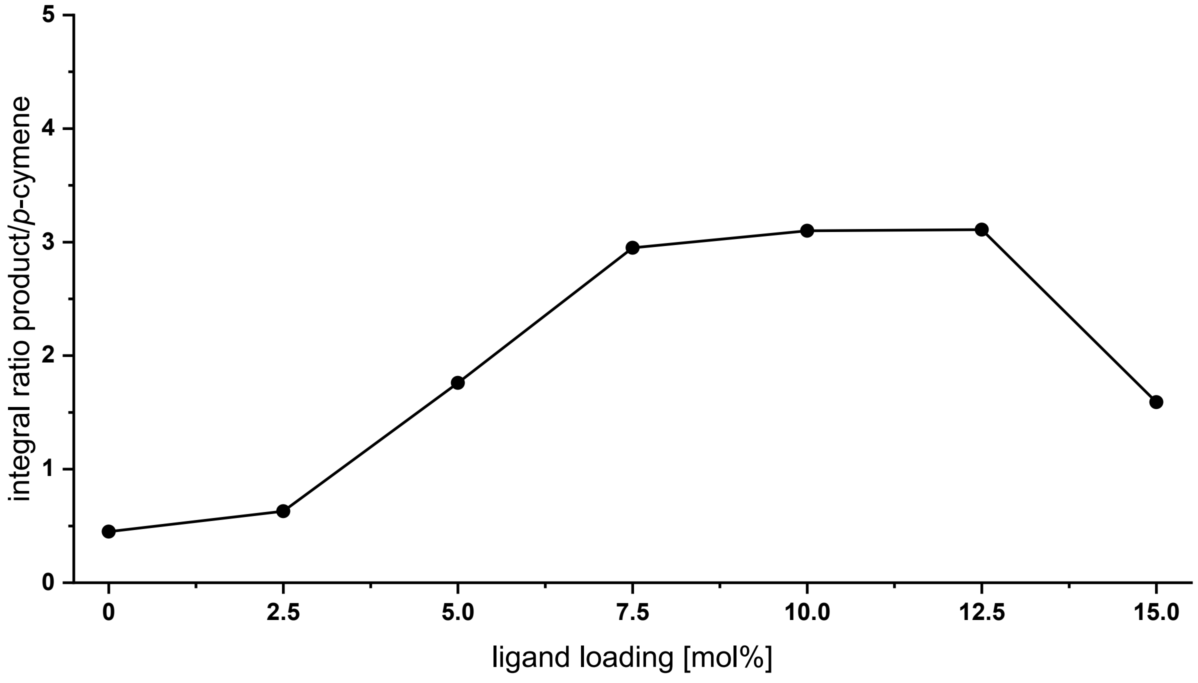

Figure S5: Ligand precursor loading screening for the alkylation of methyl pentyl sulfide (**1a**) with 1-octene (**2a**) in the presence of complex **Ti2** and ligand precursor **LH1**.

Optimization Reactions 3: Time Screening

In a nitrogen filled glovebox, methyl pentyl sulfide (**1a**, 237 mg, 2.0 mmol), 1-octene (**2a**, 337 mg, 3.0 mmol), $Ti(CH_2SiMe_3)_4$ (**Ti2**, 79 mg, 0.2 mmol, 10 mol%), ligand precursor (**LH1**, 37 mg, 55 mg, 0.15 mmol, 7.5 mol%), and *p*-cymene (54 mg, 0.4 mmol) were weighed into a volumetric flask (5 mL) and the total volume was brought up to 5 mL with toluene to give a stock solution. Individual portions of $[Ph_3C][B(C_6F_5)_4]$ (19 mg, 0.02 mmol, 10 mol%) were weighed into glass vials and an aliquot of the stock solution (500 µL, 0.2 mmol reaction scale) was added. The resulting solutions were then individually transferred into glass ampoules (1 mL) via syringe. After temporarily sealing the ampoules with silicon grease, a propane/oxygen torch was used to seal the ampoules outside of the glovebox.[3] Subsequently, they were heated in an oil bath to 110 °C for the corresponding time. The reaction mixtures were then cooled to room temperature and transferred into flasks with CH_2Cl_2 (5 mL) before being subjected to GC analysis. Ratios given in Figure S6 refer to the ratios of the integrals obtained for the hydrothiomethylation product **5a** and *p*-cymene.

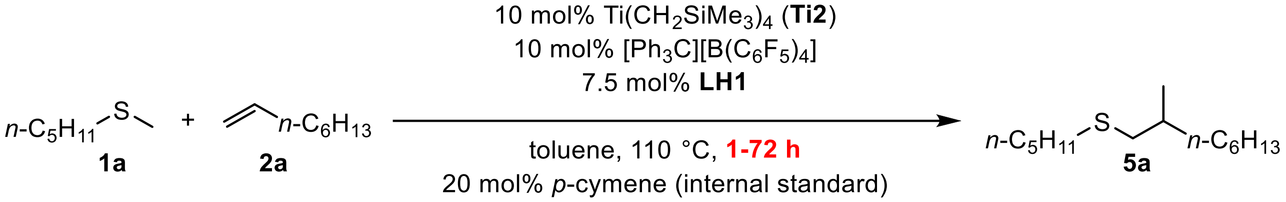

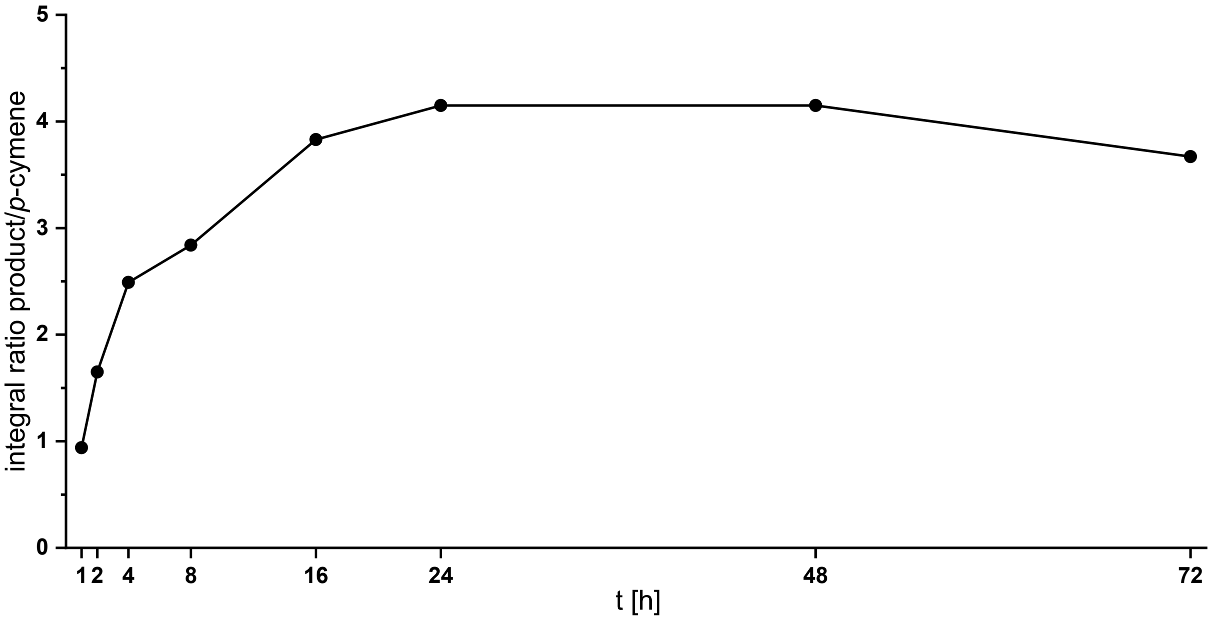

Figure S6: Time screening for the alkylation of methyl pentyl sulfide (**1a**) with 1-octene (**2a**) in the presence of complex **Ti2** and ligand precursor **LH1**.

3. Synthesis of Tetrakis[(trimethylsilyl)methyl]titanium (Ti2)[4]

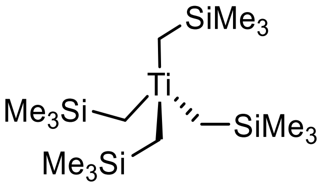

CAUTION: Even small amounts of Ti(CH_2SiMe_3)$_4$ tend to spontaneously self-ignite in ambient air. Therefore, the entire synthesis must be carried out under an inert gas atmosphere, and contaminated objects must be disposed of/cleaned with extreme care.

In a 500 mL oven-dried three-necked round-bottom flask equipped with a reflux condenser and a dropping funnel under an atmosphere of argon, magnesium turnings (4.13 g, 170 mmol) were suspended in Et_2O (200 mL). Subsequently, (chloromethyl)trimethylsilane (19.82 g, 161.6 mmol) dissolved in Et_2O (50 mL) was added dropwise to the stirred suspension, fast enough to keep the solution slightly boiling. After complete addition, the resulting Grignard solution was transferred via Teflon tubing into a dropping funnel which was attached to a 1000 mL three-necked round-bottom flask charged with titanium tetrachloride (7.59 g, 40.0 mmol) and Et_2O (150 mL). The mixture was cooled to –20 °C and the Grignard solution was added dropwise over a period of 3.5 h. Afterwards, the mixture was stirred for additional 1.5 h at 0 °C and then all volatiles were removed under reduced pressure (1×10^{-3} mbar). *n*-Hexane (100 mL) was added to the solid residue, and the resulting slurry was filtered through a pad of sodium sulfate (Schlenk frit). After washing of the solid material with *n*-hexane (2×100 mL), the solvent was removed under reduced pressure (1×10^{-3} mbar) to give Ti(CH_2SiMe_3)$_4$ (**Ti2**, 12.55 g, 31.6 mmol, 79 % yield) as a slightly brown liquid.

^{1}H NMR (500 MHz, C_6D_6): δ = 0.21 (s, 36H), 2.29 (s, 8H) ppm.

^{13}C{^{1}H} NMR (125 MHz, DEPT, C_6D_6): δ = 2.3 (CH_3), 97.6 (CH_2) ppm.

^{29}Si{^{1}H} NMR (99 MHz, INEPT, C_6D_6): δ = –3.7 ppm.

4. Catalytic Reactions

General Procedure for the Hydrothioalkylation of Alkenes

Prior to use, all sulfides and alkenes were dried with CaH_2 or molecular sieves (3 Å) and degassed. In a nitrogen filled glovebox, a 10 mL vial was charged with $[Ph_3C][B(C_6F_5)_4]$ (92 mg, 0.10 mmol, 10 mol%) and the ligand precursor **LH1** (27 mg, 0.075 mmol, 7.5 mol%). In a separate 10 mL vial, $Ti(CH_2SiMe_3)_4$ (**Ti2**, 40 mg, 0.10 mmol, 10 mol%), the sulfide (**1**, 1.0 mmol), and the alkene (**2**, 1.5 mmol) were dissolved in toluene (1.0 mL). This solution was then transferred into the vial containing $[Ph_3C][B(C_6F_5)_4]$ and **LH1**. After homogenizing the resulting suspension, the entire mixture was transferred via syringe into a 5 mL Schlenk tube equipped with a magnetic stir bar. The tube was sealed with a Teflon stopcock, removed from the glovebox, and heated to 110 °C in an aluminum heating block for 24 h. After the crude reaction mixture had been cooled to room temperature, it was diluted with toluene (2 mL) and directly purified by column chromatography (silica gel, *n*-pentane/CH_2Cl_2) to give the desired hydrothiomethylation product.

2-Methyl-1-(pentylthio)octane (5a)[5]

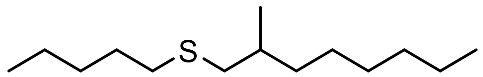

The general procedure was used to react methyl pentyl sulfide (**1a**, 118 mg, 1.0 mmol) with 1-octene (**2a**, 168 mg, 1.5 mmol) for 24 h at 110 °C. Purification by column chromatography (SiO_2, *n*-pentane/CH_2Cl_2 = 20:1, R_f = 0.43) gave product **5a** (210 mg, 0.91 mmol, 91 %) as a colorless oil.

Multigram scale reaction: In a nitrogen filled glovebox, a 80 mL Schlenk tube equipped with a magnetic stir bar was charged with $[Ph_3C][B(C_6F_5)_4]$ (922 mg, 1.0 mmol, 5 mol%) and the ligand precursor **LH1** (273 mg, 0.75 mmol, 3.75 mol%). In a separate 50 mL vial, $Ti(CH_2SiMe_3)_4$ (**Ti2**, 397 mg, 1.0 mmol, 5 mol%), methyl pentyl sulfide (**1a**, 2.37 g, 20.0 mmol), and 1-octene (**2a**, 3.37 mg, 30.0 mmol) were dissolved in toluene (20 mL). This solution was subsequently added to the Schlenk tube containing $[Ph_3C][B(C_6F_5)_4]$ and **LH1**. The tube was sealed with a Teflon stopcock, removed from the glovebox, and heated to 110 °C in an aluminum heating block for 48 h. After the crude reaction mixture had been cooled to room temperature, the solvent was carefully removed under reduced pressure. Purification of the residue by column chromatography (SiO_2, pentane/CH_2Cl_2 = 20:1, R_f = 0.43) gave product **5a** (3.89 g, 16.9 mmol, 85 %) as a colorless oil.

^{1}H NMR (500 MHz, $CDCl_3$): δ = 0.84-0.91 (m, 6H), 0.96 (d, J = 6.7 Hz, 3H), 1.10-1.48 (m, 14H), 1.53-1.66 (m, 3H), 2.29-2.37 (m, 1H), 2.43-2.54 (m, 3H) ppm.

^{13}C{^{1}H} NMR (125 MHz, DEPT, $CDCl_3$): δ = 14.1 (CH_3), 14.2 (CH_3), 19.6 (CH_3), 22.5 (CH_2), 22.8 (CH_2), 27.1 (CH_2), 29.6 (CH_2), 29.7 (CH_2), 31.3 (CH_2), 32.0 (CH_2), 33.0 (CH_2), 33.5 (CH), 36.4 (CH_2), 40.2 (CH_2) ppm.

1-[(4-Phenyl-2-methylbutyl)thio]pentane (5b)[5]

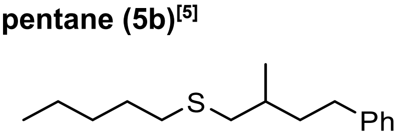

The general procedure was used to react methyl pentyl sulfide (**1a**, 118 mg, 1.0 mmol) with 4-phenyl-1-butene (198 mg, 1.5 mmol) for 24 h at 110 °C. Purification by column chromatography (SiO_2, *n*-pentane/CH_2Cl_2 = 20:1, R_f = 0.17) gave product **5b** (220 mg, 0.88 mmol, 88 %) as a colorless oil.

^{1}H NMR (500 MHz, $CDCl_3$): δ = 0.93 (t, J = 7.1 Hz, 3H), 1.08 (d, J = 6.6 Hz, 3H), 1.30-1.41 (m, 4H), 1.50-1.63 (m, 3H), 1.68-1.78 (m, 1H), 1.79-1.88 (m, 1H), 2.36-2.76 (m, 6H), 7.17-7.24 (m, 3H), 7.30 (t, J = 7.6 Hz, 2H) ppm.

$^{13}C\{^1H\}$ **NMR** (125 MHz, DEPT, $CDCl_3$): δ = 14.1 (CH_3), 19.5 (CH_3), 22.4 (CH_2), 29.6 (CH_2), 31.2 (CH_2), 32.9 (CH_2), 33.0 (CH), 33.4 (CH_2), 38.0 (CH_2), 39.9 (CH_2), 125.8 (CH), 128.4 (CH), 128.5 (CH), 142.6 (C) ppm.

1-[(3-Phenyl-2-methylpropyl)thio]pentane (5c)[5]

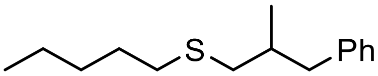

The general procedure was used to react methyl pentyl sulfide (**1a**, 118 mg, 1.0 mmol) with 3-phenylpropene (177 mg, 1.5 mmol) for 24 h at 110 °C. Purification by column chromatography (SiO_2, *n*-pentane/CH_2Cl_2 = 20:1, R_f = 0.18) gave product **5c** (198 mg, 0.84 mmol, 84 %) as a colorless oil.

^{1}**H NMR** (500 MHz, $CDCl_3$): δ = 0.92 (t, *J* = 7.0 Hz, 3H), 1.00 (d, *J* = 6.7 Hz, 3H), 1.30-1.41 (m, 4H), 1.57 (p, *J* = 7.3 Hz, 2H), 1.99 (oct, *J* = 6.9 Hz, 1H), 2.30-2.65 (m, 4H), 2.49 (dd, *J* = 7.9, 13.4 Hz, 1H), 2.81 (dd, *J* = 6.3, 13.4 Hz, 1H), 7.15-7.24 (m, 3H), 7.30 (t, *J* = 7.3 Hz, 2H) ppm.

$^{13}C\{^1H\}$ **NMR** (125 MHz, DEPT, $CDCl_3$): δ = 14.1 (CH_3), 19.4 (CH_3), 22.4 (CH_2), 29.6 (CH_2), 31.2 (CH_2), 32.9 (CH_2), 35.6 (CH), 39.3 (CH_2), 42.5 (CH_2), 126.0 (CH), 128.3 (CH), 129.3 (CH), 140.8 (C) ppm.

1-[(2,3,3-Trimethylbutyl)thio]pentane (5d)

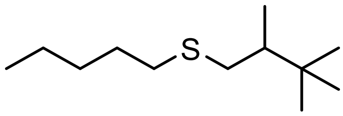

The general procedure was used to react methyl pentyl sulfide (**1a**, 118 mg, 1.0 mmol) with 3,3-dimethyl-1-butene (126 mg, 1.5 mmol) for 24 h at 110 °C. Purification by column chromatography (SiO_2, *n*-pentane/CH_2Cl_2 = 100:2, R_f = 0.31) gave product **5d** (165 mg, 0.82 mmol, 82 %) as a colorless oil.

^{1}**H NMR** (500 MHz, $CDCl_3$): δ = 0.86 (s, 9H), 0.89 (t, *J* = 7.1 Hz, 3H), 0.97 (d, *J* = 6.8 Hz, 3H), 1.28-1.41 (m, 5H), 1.52-1.62 (m, 2H), 2.07 (t, *J* = 11.6 Hz, 1H), 2.38-2.54 (m, 2H), 2.76 (d, *J* = 12.4 Hz, 1H) ppm.

$^{13}C\{^1H\}$ **NMR** (125 MHz, DEPT, $CDCl_3$): δ = 14.1 (CH_3), 14.3 (CH_3), 22.5 (CH_2), 27.4 (CH_3), 29.6 (CH_2), 31.3 (CH_2), 32.8 (CH_2), 33.3 (C), 35.7 (CH_2), 43.7 (CH) ppm.

IR (neat, ATR): λ^{-1} = 2957, 2927, 2872, 1466, 1420, 1396, 1374, 1364, 1299, 1254, 1235, 1217, 1173, 1106, 1083, 1047, 989, 967, 929, 836, 759, 731, 701 cm^{-1}.

HRMS (EI, 70 eV): *m*/*z* $[M]^+$ calcd for $C_{12}H_{26}S$: 202.1750; found 200.1747.

1-[(2-Methyl-8-phenyloctyl)thio]pentane (5e)

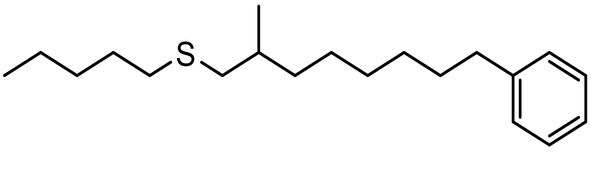

The general procedure was used to react methyl pentyl sulfide (**1a**, 118 mg, 1.0 mmol) with 8-phenyl-1-octene (283 mg, 1.5 mmol) for 24 h at 110 °C. Purification by column chromatography (SiO_2, *n*-pentane/CH_2Cl_2 = 20:1, R_f = 0.22) gave product **5e** (250 mg, 0.82 mmol, 82 %) as a colorless oil.

^{1}**H NMR** (500 MHz, $CDCl_3$): δ = 0.92 (t, J = 7.1 Hz, 3H), 0.98 (d, J = 6.6 Hz, 3H), 1.16-1.47 (m, 12H), 1.54-1.69 (m, 5H), 2.29-2.57 (m, 4H), 2.62 (t, J = 7.7 Hz, 2H), 7.16-7.21 (m, 3H), 7.26-7.31 (m, 2H) ppm.

^{13}**C{^{1}H} NMR** (125 MHz, DEPT, $CDCl_3$): δ = 14.1 (CH_3), 19.6 (CH_3), 22.5 (CH_2), 27.1 (CH_2), 29.4 (CH_2), 29.6 (CH_2), 29.8 (CH_2), 31.3 (CH_2), 31.6 (CH_2), 33.0 (CH_2), 33.5 (CH), 36.1 (CH_2), 36.4 (CH_2), 40.1 (CH_2), 125.7 (CH), 128.3 (CH), 128.5 (CH), 143.0 (C) ppm.

IR (neat, ATR): λ^{-1} = 3084, 3063, 3026, 2954, 2924, 2854, 1604, 1496, 1453, 1376, 1299, 1244, 1214, 1110, 1080, 1030, 964, 906, 746, 697, 587, 571, 494 cm^{-1}.

HRMS (EI, 70 eV): m/z [M]$^+$ calcd for $C_{20}H_{34}S$: 306.2376; found 306.2378.

Triisopropyl((4-methyl-5-(pentylthio)pentyl)oxy)silane (5f)

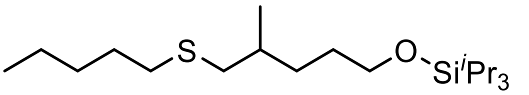

The general procedure was used to react methyl pentyl sulfide (**1a**, 118 mg, 1.0 mmol) with 5-(triisopropylsilyloxy)-1-pentene (364 mg, 1.5 mmol) for 24 h at 110 °C. Purification by column chromatography (SiO_2, *n*-pentane/CH_2Cl_2 = 10:1, R_f = 0.13) gave product **5f** (265 mg, 0.74 mmol, 74 %) as a colorless oil.

^{1}**H NMR** (500 MHz, $CDCl_3$): δ = 0.88 (t, J = 7.0 Hz, 3H), 0.98 (d, J = 6.6 Hz, 3H), 1.01-1.10 (m, 21H), 1.19-1.38 (m, 6H), 1.45-1.70 (m, 5H), 2.34 (dd, J = 7.6, 12.6 Hz, 1H), 2.43-2.54 (m, 3H), 3.66 (t, J = 6.5 Hz, 2H) ppm.

^{13}**C{^{1}H} NMR** (125 MHz, DEPT, $CDCl_3$): δ = 12.1 (CH), 14.1 (CH_3), 18.1 (CH_3), 19.5 (CH_3), 22.5 (CH_2), 29.6 (CH_2), 30.6 (CH_2), 31.3 (CH_2), 32.5 (CH_2), 33.0 (CH_2), 33.4 (CH), 40.1 (CH_2), 63.7 (CH_2) ppm.

IR (neat, ATR): λ^{-1} = 2926, 2864, 1463, 1380, 1297, 1247, 1216, 1102, 1070, 1013, 996, 937, 881, 787, 720, 679, 657, 566, 510 cm^{-1}.

HRMS (ESI+): m/z [M+H]$^+$ calcd for $C_{20}H_{45}OSSi$: 361.2955; found 361.2958.

1-[(4,4-Diphenyl-2-methylbutyl)thio]pentane (5g)

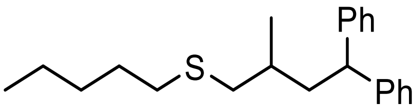

The general procedure was used to react methyl pentyl sulfide (**1a**, 118 mg, 1.0 mmol) with 4,4-diphenyl-1-butene (313 mg, 1.5 mmol) for 24 h at 110 °C. Purification by column chromatography (SiO_2, *n*-pentane/CH_2Cl_2 = 5:1, R_f = 0.22) gave product **5g** (180 mg, 0.55 mmol, 55 %) as a colorless oil.

^{1}**H NMR** (500 MHz, $CDCl_3$): δ = 0.93 (t, J = 7.1 Hz, 3H), 1.06 (d, J = 6.6 Hz, 3H), 1.26-1.38 (m, 4H), 1.49-1.65 (m, 3H), 1.84 (ddd, J = 6.4, 8.4, 13.8 Hz, 1H) 2.23-2.70 (m, 5H), 4.09 (dd, J = 6.4, 9.7 Hz, 1H), 7.17-7.23 (m, 2H), 7.27-7.33 (m, 8H) ppm.

^{13}**C{^{1}H} NMR** (125 MHz, DEPT, $CDCl_3$): δ = 14.1 (CH_3), 19.7 (CH_3), 22.4 (CH_2), 29.5 (CH_2), 30.9 (CH), 31.2 (CH_2), 32.7 (CH_2), 40.0 (CH_2), 42.1 (CH_2), 48.8 (CH), 126.2 (CH), 126.3 (CH), 127.9 (CH), 128.1 (CH), 128.6 (CH), 128.6 (CH), 144.5 (C), 145.4 (C) ppm.

IR (neat, ATR): λ^{-1} = 3062, 3026, 2954, 2924, 2870, 2857, 1600, 1583, 1493, 1450, 1417, 1376, 1299, 1246, 1184, 1156, 1074, 1031, 1001, 910, 843, 784, 747, 737, 696, 630, 620, 589, 554, 520 cm^{-1}.

HRMS (EI, 70 eV): *m*/*z* $[M]^+$ calcd for $C_{22}H_{30}S$: 326.2063; found 326.2066.

1-[(2-Methyl-3-(1,2,3,4-tetrahydronaphthalen-1-yl)propyl)thio]pentane (5h)

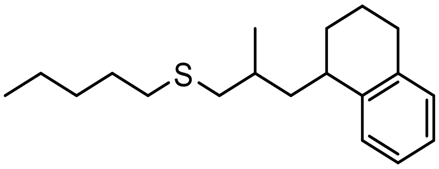

The general procedure was used to react methyl pentyl sulfide (**1a**, 118 mg, 1.0 mmol) with 1-allyl-1,2,3,4-tetrahydronaphthalene (258 mg, 1.5 mmol) for 24 h at 110 °C. Purification by column chromatography (SiO_2, *n*-pentane/CH_2Cl_2 = 5:1, R_f = 0.19) gave product **5h** (182 mg, 0.63 mmol, 63 %) as a colorless oil. The diastereomeric ratio was determined to be approximately 1:1 according to GC analysis.

^{1}H NMR (500 MHz, $CDCl_3$): δ = 0.95 (t, *J* = 7.1 Hz, 3H), 0.97 (t, *J* = 7.1 Hz, 3H), 1.13 (d, *J* = 6.6 Hz, 3H), 1.16 (d, *J* = 6.6 Hz, 3H), 1.34-1.57 (m, 10H), 1.60-1.68 (m, 4H), 1.71.-1.96 (m, 12H), 2.31-2.89 (m, 12H), 2.89-2.96 (m, 2H), 7.07-7.24 (m, 8H) ppm (mixture of 2 diastereomers).

^{13}C{^{1}H} NMR (125 MHz, DEPT, $CDCl_3$): δ = 14.1 (CH_3), 19.1 (CH_3), 19.4 (CH_2), 19.5 (CH_2), 20.8 (CH_3), 22.4 (CH_2), 26.8 (CH_2), 27.8 (CH_2), 29.6 (CH_2), 29.7 (CH_2), 31.1 (CH), 31.2 (CH_2), 31.2 (CH_2), 33.0 (CH_2), 34.9 (CH), 35.1 (CH), 39.2 (CH_2), 41.0 (CH_2), 44.0 (CH_2), 44.2 (CH_2), 125.5 (CH), 125.5 (CH), 125.6 (CH), 125.6 (CH), 128.7 (CH), 128.7 (CH), 129.1 (CH), 136.9 (C), 137.0 (C), 141.6 (C), 141.7 (C) ppm (mixture of 2 diastereomers).

IR (neat, ATR): λ^{-1} = 3059, 3016, 2924, 2857, 1602, 1579, 1490, 1450, 1376, 1274, 1244, 1216, 1160, 1114, 1040, 970, 939, 909, 870, 800, 753, 731, 647, 546 cm^{-1}.

HRMS (EI, 70 eV): *m*/*z* $[M]^+$ calcd for $C_{19}H_{30}S$: 290.2063; found 290.2069.

1-[(Cyclopentylmethyl)thio]pentane (5i)

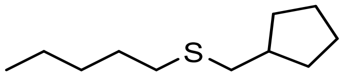

The general procedure was used to react methyl pentyl sulfide (**1a**, 118 mg, 1.0 mmol) with cyclopentene (102 mg, 1.5 mmol) for 24 h at 110 °C. Purification by column chromatography (SiO_2, *n*-pentane/CH_2Cl_2 = 100:1, R_f = 0.28) gave product **5i** (83 mg, 0.45 mmol, 45 %) as a colorless oil.

^{1}H NMR (500 MHz, $CDCl_3$): δ = 0.89 (t, *J* = 7.1 Hz, 3H), 1.18-1.26 (m, 2H), 1.28-1.39 (m, 4H), 1.48-1.66 (m, 6H), 1.76-1.86 (m, 2H), 2.04 (sept, *J* = 7.6 Hz, 1H), 2.32-2.64 (m, 4H) ppm.

^{13}C{^{1}H} NMR (125 MHz, DEPT, $CDCl_3$): δ = 14.1 (CH_3), 22.5 (CH_2), 25.3 (CH_2), 29.6 (CH_2), 31.3 (CH_2), 32.6 (CH_2), 32.8 (CH_2), 38.7 (CH_2), 40.1 (CH) ppm.

IR (neat, ATR): λ^{-1} = 2952, 2926, 2859, 1494, 1452, 1417, 1379, 1349, 1299, 1247, 1214, 1187, 1082, 1043, 964, 894, 836, 753, 731, 700, 606 cm^{-1}.

HRMS (ESI+): *m*/*z* $[M+H]^+$ calcd for $C_{11}H_{23}S$: 187.1515; found 187.1513.

1-[(Cyclohexylmethyl)thio]pentane (5j)

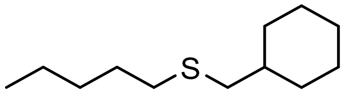

The general procedure was used to react methyl pentyl sulfide (**1a**, 118 mg, 1.0 mmol) with cyclohexene (123 mg, 1.5 mmol) for 24 h at 110 °C. Purification by column chromatography (SiO_2, *n*-pentane/CH_2Cl_2 = 100:3, R_f = 0.32) gave product **5j** (110 mg, 0.55 mmol, 55 %) as a colorless oil.

^{1}H NMR (500 MHz, $CDCl_3$): δ = 0.89 (t, J = 7.1 Hz, 3H), 0.91-0.98 (m, 2H), 1.12-1.26 (m, 3H), 1.29-1.38 (m, 4H), 1.40-1.48 (m, 1H), 1.57 (quint, J = 7.4 Hz, 2H), 1.61-1.67 (m, 1H), 1.68-1.74 (m, 2H), 1.81-1.87 (m, 2H), 2.30-2.58 (m, 4H) ppm.

^{13}C{^{1}H} NMR (125 MHz, DEPT, $CDCl_3$): δ = 14.1 (CH_3), 22.5 (CH_2), 26.3 (CH_2), 26.6 (CH_2), 29.6 (CH_2), 31.3 (CH_2), 33.0 (CH_2), 33.0 (CH_2), 38.2 (CH), 40.1 (CH_2) ppm.

IR (neat, ATR): λ^{-1} = 3009, 2922, 2850, 2674, 2616, 2536, 2277, 1693, 1420, 1289, 1224, 1203, 1177, 1143, 901, 777, 717, 653, 579, 539, 499 cm^{-1}.

HRMS (EI, 70 eV): *m*/*z* [M]$^+$ calcd for $C_{12}H_{24}S$: 200.1593; found 200.1589.

[2-(Cyclohex-3-en-1-yl)propylthio]pentane (5k)

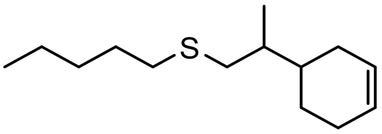

The general procedure was used to react methyl pentyl sulfide (**1a**, 118 mg, 1.0 mmol) with 4-vinyl-1-cyclohexene (162 mg, 1.5 mmol) for 24 h at 110 °C. Purification by column chromatography (SiO_2, *n*-pentane/CH_2Cl_2 = 50:1, R_f = 0.26) gave product **5k** (164 mg, 0.72 mmol, 72 %) as a colorless oil. The diastereomeric ratio was estimated to be approximately 1:1 according to ^{13}C{^{1}H} NMR analysis.

^{1}H NMR (500 MHz, $(CD_3)_2CO$): δ = 0.89 (t, J = 7.0 Hz, 6H), 0.96 (d, J = 6.6 Hz, 3H), 0.98 (d, J = 6.8 Hz, 3H), 1.19-1.41 (m, 10H), 1.54-1.68 (m, 8H), 1.69-1.88 (m, 4H), 1.93-2.04 (m, 6H), 2.37 (dd, J = 8.0, 13.2 Hz, 1H), 2.39 (dd, J = 8.1, 13.0 Hz, 1H), 2.49 (t, J = 7.4 Hz, 2H), 2.50 (t, J = 7.4 Hz, 2H), 2.63 (d, J = 4.4 Hz, 1H), 2.66 (d, J = 5.3 Hz, 1H), 5.59-5.69 (m, 4H) ppm (mixture of 2 diastereomers).

^{13}C{^{1}H} NMR (125 MHz, DEPT, $(CD_3)_2CO$): δ = 14.3 (CH_3), 15.9 (CH_3), 16.3 (CH_3), 23.0 (CH_2), 25.6 (CH_2), 26.5 (CH_2), 26.6 (CH_2), 27.5 (CH_2), 27.9 (CH_2), 30.2 (CH_2), 31.8 (CH_2), 33.0 (CH_2), 33.1 (CH_2), 37.7 (CH_2), 37.8 (CH_2), 38.4 (CH), 38.5 (CH), 38.6 (CH), 38.6 (CH), 127.5 (CH), 127.5 (CH), 127.6 (CH), 127.6 (CH) ppm (mixture of 2 diastereomers).

IR (neat, ATR): λ^{-1} = 3022, 2956, 2922, 2873, 2857, 2839, 1676, 1653, 1456, 1436, 1377, 1339, 1299, 1274, 1244, 1216, 1146, 1107, 1043, 961, 943, 927, 911, 883, 791, 753, 720, 700, 656, 606, 551 cm^{-1}.

HRMS (EI, 70 eV): *m*/*z* [M]$^+$ calcd for $C_{14}H_{26}S$: 226.1750; found 226.1748.

1-[(*E*)-(2-Methyltridec-11-en-1-yl)thio]pentane (5l)

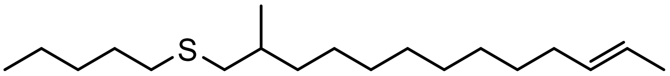

The general procedure was used to react methyl pentyl sulfide (**1a**, 118 mg, 1.0 mmol) with (*E*)-trideca-1,11-diene (270 mg, 1.5 mmol) for 24 h at 110 °C. Purification by column chromatography (SiO_2, *n*-pentane/CH_2Cl_2 = 20:1, R_f = 0.38) gave product **5l** (199 mg, 0.67 mmol, 67 %) as a colorless oil.

^{1}H NMR (500 MHz, $CDCl_3$): δ = 0.89 (t, *J* = 7.1 Hz, 3H), 0.96 (d, *J* = 6.6 Hz, 3H), 1.11-1.45 (m, 19H), 1.57 (quint, *J* = 7.2 Hz, 2H), 1.63 (br. d, *J* = 4.3 Hz, 3H), 1.90-2.04 (m, 2H), 2.27-2.56 (m, 4H), 5.35-5.45 (m, 2H) ppm.

^{13}C{^{1}H} NMR (125 MHz, DEPT, $CDCl_3$): δ = 14.1 (CH_3), 18.0 (CH_3), 19.6 (CH_3), 22.5 (CH_2), 27.1 (CH_2), 29.3 (CH_2), 29.6 (CH_2), 29.7 (CH_2), 29.7 (CH_2), 30.0 (CH_2), 31.3 (CH_2), 32.7 (CH_2), 33.0 (CH_2), 33.5 (CH), 36.4 (CH_2), 40.1 (CH_2), 124.6 (CH), 131.8 (CH) ppm.

IR (neat, ATR): λ^{-1} = 2956, 2923, 2853, 1739, 1456, 1376, 1299, 1243, 1217, 964, 722 cm^{-1}.

HRMS (EI, 70 eV): *m*/*z* $[M]^+$ calcd for $C_{19}H_{38}S$: 298.2689; found 298.2684.

1-[(4-(2-Bromophenyl)-2-methylbutyl)thio]pentane (5m)

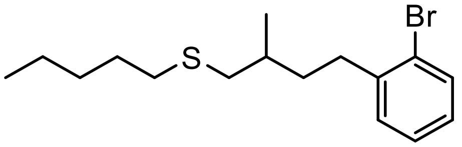

The general procedure was used to react methyl pentyl sulfide (**1a**, 118 mg, 1.0 mmol) with 4-(2-bromophenyl)-1-butene (317 mg, 1.5 mmol) for 24 h at 110 °C. Purification by column chromatography (SiO_2, *n*-pentane/CH_2Cl_2 = 20:1, R_f = 0.17) gave product **5m** (260 mg, 0.79 mmol, 79 %) as a colorless oil.

^{1}H NMR (500 MHz, $CDCl_3$): δ = 0.91 (t, *J* = 7.1 Hz, 3H), 1.10 (d, *J* = 6.6 Hz, 3H), 1.28-1.41 (m, 4H), 1.45-1.55 (m, 1H), 1.55-1.63 (m, 2H), 1.70-1.84 (m, 2H), 2.35-2.65 (m, 4H), 2.67-2.84 (m, 2H), 7.01-7.07 (m, 1H), 7.20-7.25 (m, 2H), 7.52 (d, *J* = 7.6 Hz, 1H) ppm.

^{13}C{^{1}H} NMR (125 MHz, DEPT, $CDCl_3$): δ = 14.1 (CH_3), 19.5 (CH_3), 22.4 (CH_2), 29.6 (CH_2), 31.2 (CH_2), 32.9 (CH_2), 33.4 (CH), 33.8 (CH_2), 36.5 (CH_2), 39.8 (CH_2), 124.5 (C), 127.5 (CH), 127.5 (CH), 130.3 (CH), 132.9 (CH), 141.9 (C) ppm.

IR (neat, ATR): λ^{-1} = 3057, 2954, 2924, 2859, 1567, 1470, 1456, 1439, 1376, 1216, 1044, 1021, 940, 746, 659, 527 cm^{-1}.

HRMS (EI, 70 eV): *m*/*z* $[M]^+$ calcd for $C_{16}H_{25}S^{79}Br$: 328.0855; found 328.0853.

1-[(4-(4-Chlorophenyl)-2-methylbutyl)thio]pentane (5n)

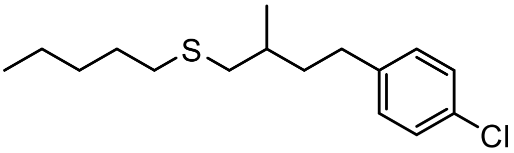

The general procedure was used to react methyl pentyl sulfide (**1a**, 118 mg, 1.0 mmol) with 4-(4-chlorophenyl)-1-butene (250 mg, 1.5 mmol) for 24 h at 110 °C. Purification by column chromatography (SiO_2, *n*-pentane/CH_2Cl_2 = 20:1, R_f = 0.21) gave product **5n** (245 mg, 0.86 mmol, 86 %) as a colorless oil.

^{1}H NMR (500 MHz, $CDCl_3$): δ = 0.91 (t, J = 7.1 Hz, 3H), 1.05 (d, J = 6.7 Hz, 3H), 1.26-1.40 (m, 4H), 1.43-1.52 (m, 1H), 1.57 (quint, J = 7.4 Hz, 2H), 1.63-1.73 (m, 1H), 1.75-1.84 (m, 1H), 2.35-2.70 (m, 6H), 7.11 (d, J = 8.5 Hz, 2H), 7.23 (d, J = 8.4 Hz, 2H) ppm.

^{13}C{^{1}H} NMR (125 MHz, DEPT, $CDCl_3$): δ = 14.1 (CH_3), 19.4 (CH_3), 22.4 (CH_2), 29.5 (CH_2), 31.2 (CH_2), 32.7 (CH_2), 32.9 (CH), 32.9 (CH_2), 37.7 (CH_2), 39.8 (CH_2), 128.4 (CH), 129.8 (CH), 131.4 (C), 141.0 (C) ppm.

IR (neat, ATR): λ^{-1} = 2956, 2926, 2857, 1597, 1492, 1457, 1407, 1376, 1299, 1274, 1244, 1214, 1092, 1014, 957, 929, 813, 751, 731, 713, 660, 629, 526, 511 cm^{-1}.

HRMS (EI, 70 eV): *m*/*z* $[M]^+$ calcd for $C_{16}H_{25}S^{35}Cl$: 284.1360; found 284.1358.

1-[(2-Methyloctyl)thio]decane (5o)

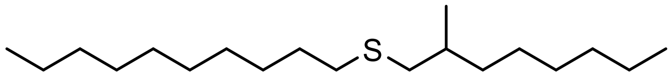

The general procedure was used to react methyl decyl sulfide (188 mg, 1.0 mmol) with 1-octene (**2a**, 168 mg, 1.5 mmol) for 24 h at 110 °C. Purification by column chromatography (SiO_2, *n*-pentane/CH_2Cl_2 = 20:1, R_f = 0.46) gave product **5o** (260 mg, 0.87 mmol, 87 %) as a colorless oil.

^{1}H NMR (500 MHz, $CDCl_3$): δ = 0.87 (t, J = 7.0 Hz, 3H), 0.88 (t, J = 6.9 Hz, 3H), 0.96 (d, J = 6.7, 3H), 1.09-1.45 (m, 24H), 1.52-1.66 (m, 3H), 2.33 (dd, J = 7.5, 12.4 Hz, 1H), 2.44-2.54 (m, 3H) ppm.

^{13}C{^{1}H} NMR (125 MHz, DEPT, $CDCl_3$): δ = 14.2 (CH_3), 19.6 (CH_3), 22.8 (CH_2), 22.8 (CH_2), 27.1 (CH_2), 29.1 (CH_2), 29.4 (CH_2), 29.5 (CH_2), 29.7 (CH_2), 29.7 (CH_2), 29.7 (CH_2), 29.9 (CH_2), 32.0 (CH_2), 32.0 (CH_2), 33.0 (CH_2), 33.5 (CH), 36.4 (CH_2), 40.2 (CH_2) ppm.

IR (neat, ATR): λ^{-1} = 2956, 2922, 2853, 1457, 1377, 1242, 721 cm^{-1}.

HRMS (EI, 70 eV): *m*/*z* $[M]^+$ calcd for $C_{19}H_{40}S$: 300.2845; found 300.2851.

1-[(2-Methyloctyl)thio]-2-phenylethane (5p)

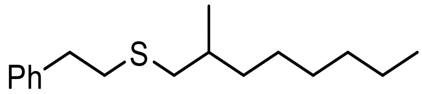

The general procedure was used to react methyl 2-phenylethyl sulfide (152 mg, 1.0 mmol) with 1-octene (**2a**, 168 mg, 1.5 mmol) for 24 h at 110 °C. Purification by column chromatography (SiO_2, *n*-pentane/CH_2Cl_2 = 10:1, R_f = 0.22) gave product **5p** (178 mg, 0.67 mmol, 67 %) as a colorless oil.

^{1}H NMR (500 MHz, $CDCl_3$): δ = 0.89 (t, J = 6.7 Hz, 3H), 0.97 (d, J = 6.8 Hz, 3H), 1.12-1.20 (m, 1H), 1.21-1.34 (m, 8H), 1.38-1.46 (m, 1H), 1.59-1.68 (m, 1H), 2.37 (dd, J = 7.6, 12.5 Hz, 1H), 2.54 (dd, J = 5.7, 12.5 Hz, 1H), 2.71-2.77 (m, 2H), 2.84-2.91 (m, 2H), 7.18-7.23 (m, 3H), 7.27-7.32 (m, 2H) ppm.

^{13}C{^{1}H} NMR (125 MHz, DEPT, $CDCl_3$): δ = 14.3 (CH_3), 19.6 (CH_3), 22.8 (CH_2), 27.1 (CH_2), 29.7 (CH_2), 32.0 (CH_2), 33.5 (CH), 34.5 (CH_2), 36.4 (CH_2), 36.7 (CH_2), 40.3 (CH_2), 126.4 (CH), 128.6 (CH), 128.6 (CH), 140.9 (C) ppm.

IR (neat, ATR): λ^{-1} = 3063, 3027, 2954, 2923, 2854, 1604, 1496, 1453, 1376, 1273, 1224, 1073, 1030, 733, 696, 563, 491 cm^{-1}.

HRMS (EI, 70 eV): m/z $[M]^+$ calcd for $C_{17}H_{28}S$: 264.1906; found 264.1913.

(2-Methyloctyl)thioethane (5q)

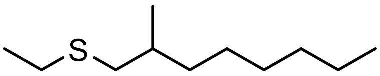

The general procedure was used to react ethyl methyl sulfide (76 mg, 1.0 mmol) with 1-octene (**2a**, 168 mg, 1.5 mmol) for 24 h at 110 °C. Purification by column chromatography (SiO_2, *n*-pentane/CH_2Cl_2 = 20:1, R_f = 0.22) gave product **5q** (150 mg, 0.80 mmol, 80 %) as a colorless oil.

^{1}H NMR (500 MHz, $CDCl_3$): δ = 0.87 (t, J = 6.8 Hz, 3H), 0.96 (d, J = 6.8 Hz, 3H), 1.11-1.18 (m, 1H), 1.20-1.34 (m, 11H), 1.38-1.45 (m, 1H), 1.58-1.67 (m, 1H), 2.34 (dd, J = 7.6, 12.7 Hz, 1H), 2.47-2.54 (m, 3H) ppm.

^{13}C{^{1}H} NMR (125 MHz, DEPT, $CDCl_3$): δ = 14.2 (CH_3), 15.0 (CH_3), 19.5 (CH_3), 22.8 (CH_2), 26.7 (CH_2), 27.1 (CH_2), 29.6 (CH_2), 32.0 (CH_2), 33.4 (CH), 36.4 (CH_2), 39.7 (CH_2) ppm.

IR (neat, ATR): λ^{-1} = 2957, 2924, 2854, 1739, 1456, 1376, 1264, 1240, 1082, 969, 890, 784, 723, 700 cm^{-1}.

HRMS (EI, 70 eV): m/z $[M]^+$ calcd for $C_{11}H_{24}S$: 188.1593; found 188.1591.

1-[(2-Methyloctyl)thio]-2-methylpropane (5r)

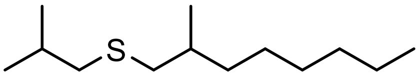

The general procedure was used to react isobutyl methyl sulfide (104 mg, 1.0 mmol) with 1-octene (**2a**, 168 mg, 1.5 mmol) for 24 h at 110 °C. Purification by column chromatography (SiO_2, *n*-pentane/CH_2Cl_2 = 20:1, R_f = 0.54) gave product **5r** (192 mg, 0.89 mmol, 89 %) as a colorless oil.

^{1}H NMR (500 MHz, $CDCl_3$): δ = 0.87 (t, J = 6.3 Hz, 3H), 0.94-1.00 (m, 9H), 1.10-1.47 (m, 10H), 1.57-1.67 (m, 1H), 1.72-1.83 (m, 1H), 2.29-2.40 (m, 3H), 2.48 (dd, J = 5.8, 12.5 Hz, 1H) ppm.

^{13}C{^{1}H} NMR (125 MHz, DEPT, $CDCl_3$): δ = 14.2 (CH_3), 19.6 (CH_3), 22.2 (CH_3), 22.2 (CH_3), 22.8 (CH_2), 27.1 (CH_2), 28.8 (CH), 29.7 (CH_2), 32.0 (CH_2), 33.6 (CH), 36.4 (CH_2), 40.8 (CH_2), 42.4 (CH_2) ppm.

IR (neat, ATR): λ^{-1} = 2956, 2924, 2870, 2854, 1463, 1377, 1366, 1239, 1169, 943, 921, 804, 756, 723 cm^{-1}.

HRMS (EI, 70 eV): m/z $[M]^+$ calcd for $C_{13}H_{28}S$: 216.1906; found 216.1913.

2-[(2-Methyloctyl)thio]propane (5s)

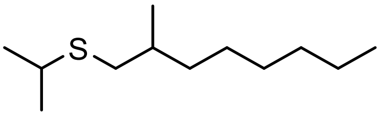

The general procedure was used to react Isopropyl methyl sulfide (90 mg, 1.0 mmol) with 1-octene (**2a**, 168 mg, 1.5 mmol) for 24 h at 110 °C. Purification by column chromatography (SiO_2, *n*-pentane/CH_2Cl_2 = 20:1, R_f = 0.45) gave product **5s** (146 mg, 0.72 mmol, 72 %) as a colorless oil.

^{1}H NMR (500 MHz, $CDCl_3$): δ = 0.87 (t, J = 6.8 Hz, 3H), 0.96 (d, J = 6.7 Hz, 3H), 1.10-1.45 (m, 16H), 1.62 (dq, J = 6.4, 12.9 Hz, 1H), 2.34 (dd, J = 7.6, 12.2 Hz, 1H), 2.51 (dd, J = 5.8, 12.3 Hz, 1H), 2.85 (sept, J = 6.7, 1H) ppm.

^{13}C{^{1}H} NMR (125 MHz, DEPT, $CDCl_3$): δ = 14.2 (CH_3), 19.6 (CH_3), 22.8 (CH_2), 23.6 (CH_3), 27.1 (CH_2), 29.6 (CH_2), 32.0 (CH_2), 33.7 (CH), 35.4 (CH), 36.5 (CH_2), 38.4 (CH_2) ppm.

IR (neat, ATR): λ^{-1} = 2956, 2924, 2856, 1739, 1457, 1377, 1364, 1236, 1156, 1051, 927, 883, 723, 649 cm^{-1}.

HRMS (EI, 70 eV): *m*/*z* $[M]^+$ calcd for $C_{12}H_{26}S$: 202.1750; found 202.1748.

(2-Methyloctyl)thiocyclohexane (5t)

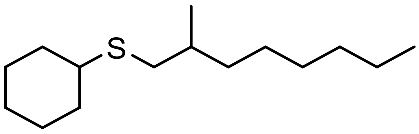

The general procedure was used to react cyclohexyl methyl sulfide (130 mg, 1.0 mmol) with 1-octene (**2a**, 168 mg, 1.5 mmol) for 24 h at 110 °C. Purification by column chromatography (SiO_2, *n*-pentane/CH_2Cl_2 = 20:1, R_f = 0.34) gave product **5t** (172 mg, 0.71 mmol, 71 %) as a colorless oil.

^{1}H NMR (500 MHz, $CDCl_3$): δ = 0.88 (t, J = 6.8 Hz, 3H), 0.96 (d, J = 6.7 Hz, 3H), 1.11-1.35 (m, 14H), 1.38-1.46 (m, 1H), 1.56-1.65 (m, 2H), 1.72-1.78 (m, 2H), 1.93-2.00 (m, 2H), 2.36 (dd, J = 7.5, 12.3 Hz, 1H), 2.48-2.60 (m, 2H) ppm.

^{13}C{^{1}H} NMR (125 MHz, DEPT, $CDCl_3$): δ = 14.2 (CH_3), 19.7 (CH_3), 22.8 (CH_2), 26.0 (CH_2), 26.3 (CH_2), 26.3 (CH_2), 27.1 (CH_2), 29.7 (CH_2), 32.0 (CH_2), 33.8 (CH), 34.0 (CH_2), 36.5 (CH_2), 38.0 (CH_2), 44.2 (CH) ppm.

IR (neat, ATR): λ^{-1} = 2954, 2923, 2852, 1449, 1376, 1342, 1263, 1202, 999, 886, 820, 743, 724 cm^{-1}.

HRMS (EI, 70 eV): *m*/*z* $[M]^+$ calcd for $C_{15}H_{30}S$: 242.2063; found 242.2063.

(2-Methyloctyl)thiomethane (5u)

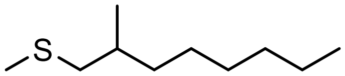

The general procedure was used to react dimethylsulfide (93 mg, 1.5 mmol) with 1-octene (**2a**, 112 mg, 1.0 mmol) for 24 h at 110 °C. Purification by column chromatography (SiO_2, *n*-pentane/CH_2Cl_2 = 20:1, R_f = 0.32) gave product **5u** (102 mg, 0.59 mmol, 59 %) as a colorless oil.

^{1}**H NMR** (500 MHz, $CDCl_3$): δ = 0.88 (t, *J* = 6.8 Hz, 3H), 0.97 (d, *J* = 6.7 Hz, 3H), 1.12-1.20 (m, 1H), 1.22-1.35 (m, 8H), 1.37-1.47 (m, 1H), 1.60-1.71 (m, 1H), 2.09 (s, 3H), 2.30-2.37 (m, 1H), 2.46-2.53 (m, 1H) ppm.

^{13}**C{^{1}H} NMR** (125 MHz, DEPT, $CDCl_3$): δ = 14.2 (CH_3), 16.4 (CH_3), 19.5 (CH_3), 22.8 (CH_2), 27.1 (CH_2), 29.7 (CH_2), 32.0 (CH_2), 33.0 (CH), 36.4 (CH_2), 42.4 (CH_2) ppm.

IR (neat, ATR): λ^{-1} = 2956, 2923, 2854, 1494, 1457, 1376, 1317, 1246, 1187, 1082, 961, 894, 816, 754, 723, 700, 647, 614 cm^{-1}.

HRMS (EI, 70 eV): *m*/*z* $[M]^+$ calcd for $C_{10}H_{22}S$: 174.1437; found 174.1437.

Bis(2-methyloctyl)sulfide (5v)

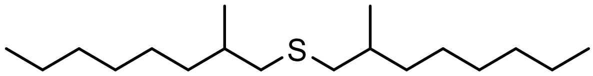

The general procedure was used to react dimethylsulfide (62 mg, 1.0 mmol) with 1-octene (**2a**, 337 mg, 3.0 mmol) for 24 h at 110 °C. Purification by column chromatography (SiO_2, *n*-pentane/CH_2Cl_2 = 20:1, R_f = 0.44) gave product **5v** (181 mg, 0.63 mmol, 63 %) as a colorless oil. The diastereomeric ratio was estimated to be approximately 1:1 according to ^{13}C{^{1}H} NMR analysis.

^{1}**H NMR** (500 MHz, $CDCl_3$): δ = 0.88 (t, *J* = 6.8 Hz, 6H), 0.97 (d, *J* = 6.7 Hz, 6H), 1.11-1.20 (m, 2H), 1.22-1.35 (m, 16H), 1.39-1.47 (m, 2H), 1.57-1.66 (m, 2H), 2.28-2.35 (m, 2H), 2.44-2.51 (m, 2H) ppm (mixture of 2 diastereomers).

^{13}**C{^{1}H} NMR** (125 MHz, DEPT, $CDCl_3$): δ = 14.2 (CH_3), 19.6 (CH_3), 19.6 (CH_3), 22.8 (CH_2), 27.1 (CH_2), 27.1 (CH_2), 29.7 (CH_2), 32.0 (CH_2), 33.6 (CH), 33.6 (CH), 36.4 (CH_2), 36.4 (CH_2), 40.8 (CH_2), 40.9 (CH_2) ppm (mixture of 2 diastereomers).

IR (neat, ATR): λ^{-1} = 2956, 2923, 2854, 1457, 1376, 1237, 1152, 1082, 967, 940, 889, 723 cm^{-1}.

HRMS (EI, 70 eV): *m*/*z* $[M]^+$ calcd for $C_{18}H_{38}S$: 286.2689; found 286.2689.

[(2-Methyloctyl)thio]methyltrimethylsilan (5w)

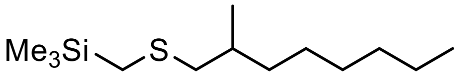

The general procedure was used to react methyl trimethylsilylmethyl sulfide (134 mg, 1.0 mmol) with 1-octene (**2a**, 168 mg, 1.5 mmol) for 24 h at 110 °C. Purification by column chromatography (SiO_2, *n*-pentane/CH_2Cl_2 = 20:1, R_f = 0.45) gave product **5w** (228 mg, 0.93 mmol, 93 %) as a colorless oil.

^{1}**H NMR** (500 MHz, $CDCl_3$): δ = 0.08 (s, 9 H), 0.87 (t, *J* = 6.8 Hz, 3H), 0.95 (d, *J* = 6.7 Hz, 3H), 1.09-1.46 (m, 10H), 1.60-1.78 (m, 3H), 2.33 (dd, *J* = 8.0, 12.0 Hz, 1H), 2.50 (dd, *J* = 5.6, 12.2 Hz, 1H) ppm.

^{13}**C{^{1}H} NMR** (125 MHz, DEPT, $CDCl_3$): δ = −1.6 (CH_3), 14.2 (CH_3), 19.2 (CH_2), 19.5 (CH_3), 22.8 (CH_2), 27.1 (CH_2), 29.6 (CH_2), 32.0 (CH_2), 32.9 (CH), 36.4 (CH_2), 44.4 (CH_2) ppm.

IR (neat, ATR): λ^{-1} = 2956, 2924, 2873, 2854, 1457, 1392, 1376, 1249, 1130, 839, 771, 751, 724, 696, 656 cm^{-1}.

HRMS (EI, 70 eV): *m*/*z* $[M]^+$ calcd for $C_{13}H_{30}SSi$: 246.1832; found 246.1831.

1-[(2-Methyloctyl)thio]-2-(4-bromophenyl)ethane (5x)

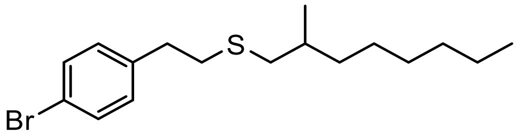

The general procedure was used to react 1-(methylthio)-2-(4-bromophenyl)ethane (231 mg, 1.0 mmol) with 1-octene (**2a**, 168 mg, 1.5 mmol) for 24 h at 110 °C. Purification by column chromatography (SiO_2, *n*-pentane/CH_2Cl_2 = 10:1, R_f = 0.45) gave product **5x** (203 mg, 0.59 mmol, 59 %) as a colorless oil.

^{1}H NMR (500 MHz, $CDCl_3$): δ = 0.89 (t, J = 6.8 Hz, 3H), 0.97 (d, J = 6.7 Hz, 3H), 1.12-1.35 (m, 9H), 1.38-1.45 (m, 1H), 1.59-1.67 (m, 1H), 2.30-2.40 (m, 1H), 2.48-2.57 (m, 1H), 2.67-2.78 (m, 2H), 2.81-2.86 (m, 2H), 7.41 (br. d, J = 8.5 Hz, 1H), 7.08 (br. d, J = 8.5 Hz, 1H) ppm.

^{13}C{^{1}H} NMR (125 MHz, DEPT, $CDCl_3$): δ = 14.2 (CH_3), 19.6 (CH_3), 22.8 (CH_2), 27.1 (CH_2), 29.6 (CH_2), 32.0 (CH_2), 33.5 (CH), 34.3 (CH_2), 35.9 (CH_2), 36.4 (CH_2), 40.3 (CH_2), 120.2 (C), 130.4 (CH), 131.6 (CH), 139.8 (C) ppm.

IR (neat, ATR): λ^{-1} = 2954, 2923, 2854, 1488, 1456, 1403, 1377, 1072, 1011, 842, 799, 699, 524, 495 cm^{-1}.

HRMS (EI, 70 eV): *m*/*z* $[M]^+$ calcd for $C_{17}H_{27}{}^{79}BrS$: 342.1011; found 342.1005.

Triisopropyl((6-((2-methyloctyl)thio)hexyl)oxy)silane (5y)

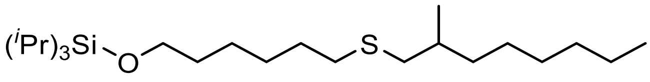

The general procedure was used to react triisopropyl((6-(methylthio)hexyl)oxy)silane (305 mg, 1.0 mmol) with 1-octene (**2a**, 168 mg, 1.5 mmol) for 24 h at 110 °C. Purification by column chromatography (SiO_2, *n*-pentane/CH_2Cl_2 = 5:1, R_f = 0.50) gave product **5y** (176 mg, 0.42 mmol, 42 %) as a colorless oil.

^{1}H NMR (500 MHz, $CDCl_3$): δ = 0.93 (t, J = 6.8 Hz, 3H), 1.02 (d, J = 6.6 Hz, 3H), 1.08-1.13 (m, 21H), 1.20-1.15 (m, 15H), 1.55-1.71 (m, 5H), 2.35-2.60 (m, 3H), 3.72 (t, J = 6.6 Hz, 2H) ppm.

^{13}C{^{1}H} NMR (125 MHz, DEPT, $CDCl_3$): δ= 12.2 (CH_3), 14.2 (CH_3), 18.2 (CH_3), 19.6 (CH), 22.8 (CH_2), 25.6 (CH_2), 27.1 (CH_2), 28.9 (CH_2), 29.7 (CH_2), 30.0 (CH_2), 32.0 (CH_2), 32.9 (CH_2), 33.0 (CH_2), 33.5 (CH), 36.4 (CH_2), 40.2 (CH_2), 63.5 (CH_2) ppm.

IR (neat, ATR): λ^{-1} = 2924, 2864, 1463, 1379, 1246, 1106, 1069, 1013, 996, 919, 881, 789, 719, 679, 657 cm^{-1}.

HRMS (EI, 70 eV): *m*/*z* $[M]^+$ calcd for $C_{24}H_{52}OSSi$: 416.3503; found 416.3493.

Unreactive Substrates

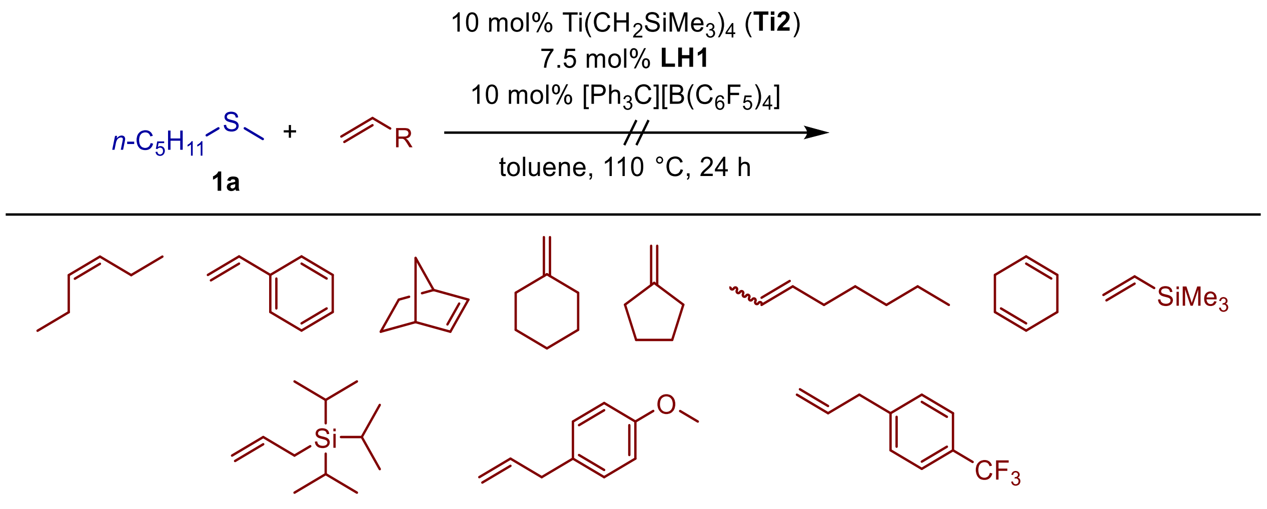

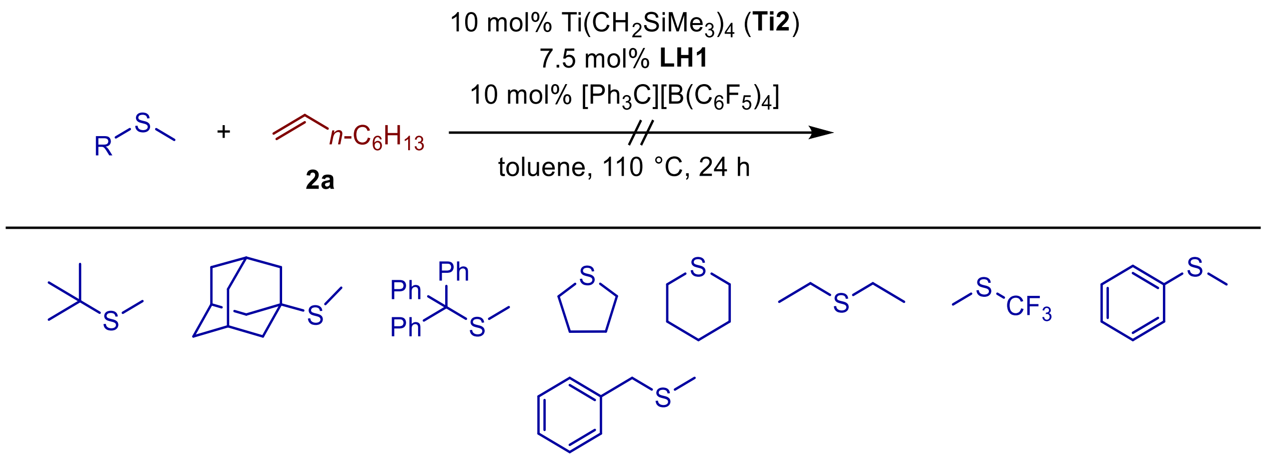

Figure S7: Unreactive substrates in the titanium-catalyzed hydrothiomethylation of alkenes with alkyl sulfides.

5. Mechanistic Investigations

2-Methyl-d1-1-(pentylthio)-1,1-d2-octane (*d*-5a)

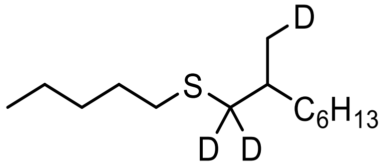

The general procedure was used to react methyl-d3 pentyl sulfide (***d*-1a**, 121 mg, 1.0 mmol) with 1-octene (**2a**, 168 mg, 1.5 mmol) for 48 h at 110 °C. Purification by column chromatography (SiO_2, *n*-pentane/CH_2Cl_2 = 20:1, R_f = 0.41) gave product ***d*-5a** (115 mg, 0.49 mmol, 49 %) as a colorless oil.

^{1}H NMR (500 MHz, $CDCl_3$): δ = 0.85-0.91 (m, 6H), 0.92-0.97 (m, 2H), 1.12-1.44 (m, 14H), 1.51-1.65 (m, 3H), 2.27-2.59 (m, 2H) ppm.

^{13}C{^{1}H} NMR (125 MHz, DEPT, $CDCl_3$): δ = 14.1 (CH_3), 14.2 (CH_3), 19.2 (t, J = 19 Hz, CH_2D), 22.5 (CH_2), 22.8 (CH_2), 27.1 (CH_2), 29.7 (CH_2), 31.3 (CH_2), 32.0 (CH_2), 32.9 (CH_2), 33.2 (CH), 36.3 (CH_2), 39.4 (pent, J = 21 Hz, CD_2) ppm.

IR (neat, ATR): λ^{-1} = 2956, 2923, 2855, 1737, 1466, 1457, 1377, 1299, 1274, 1217, 1129, 1049, 999, 967, 890, 724, 702, 669 cm^{-1}.

HRMS (EI, 70 eV): m/z $[M]^+$ calcd for $C_{14}H_{27}D_3S$: 233.2251; found 233.2248.

Kinetic Investigations

In a nitrogen filled glovebox, two 60 mL Schlenk tubes equipped with magnetic stir bars were each charged with $[Ph_3C][B(C_6F_5)_4]$ (922 mg, 1.0 mmol, 10 mol%) and the ligand precursor **LH1** (273 mg, 0.75 mmol, 7.5 mol%). In two separate 20 mL vials, $Ti(CH_2SiMe_3)_4$ (**Ti2**, 397 mg, 1.0 mmol, 10 mol%), methyl 2-phenylethyl sulfide (1.52 g, 10.0 mmol) or methyl-d3-2-phenylethyl sulfide (1.55 g, 10.0 mmol), 1-octene (**2a**, 1.68 g, 15.0 mmol) and *p*-cymene (134 mg, 1.0 mmol, 10 mol%) were dissolved in toluene (10 mL). The resulting solutions were each added to one of the Schlenk tubes containing $[Ph_3C][B(C_6F_5)_4]$ and **LH1**. The tubes were sealed with Teflon stopcocks, removed from the glovebox, and heated to 110 °C in an aluminum heating block. Every 15 minutes samples of both reaction mixtures (50 µL each) were taken under argon counterflow, quenched with CH_2Cl_2 and subsequently subjected to GC-analysis. Ratios given in Figure S7 refer to the ratios of the integrals obtained for the hydrothiomethylation products and *p*-cymene.

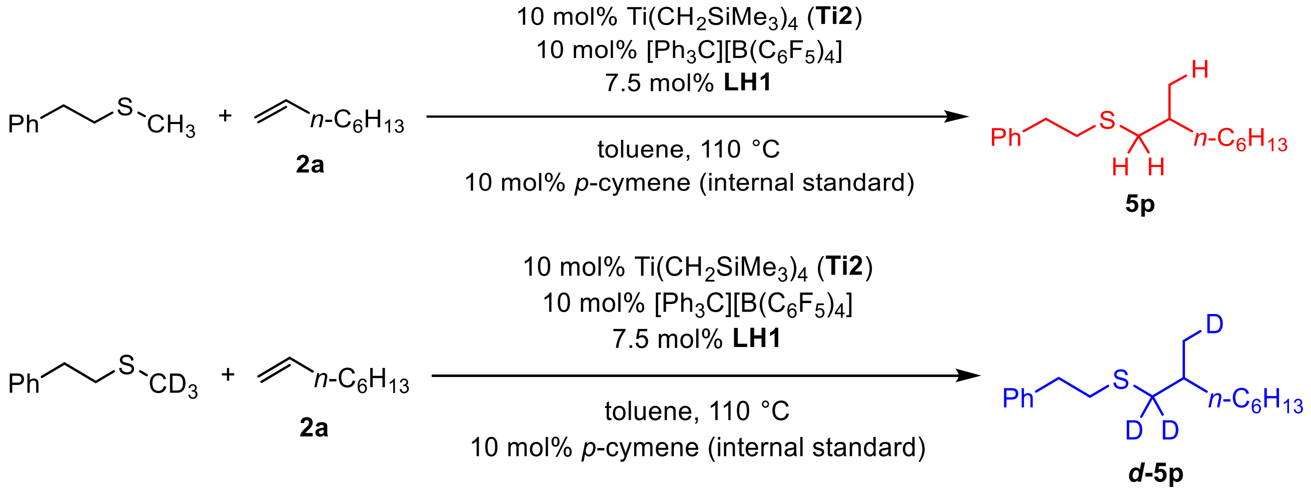

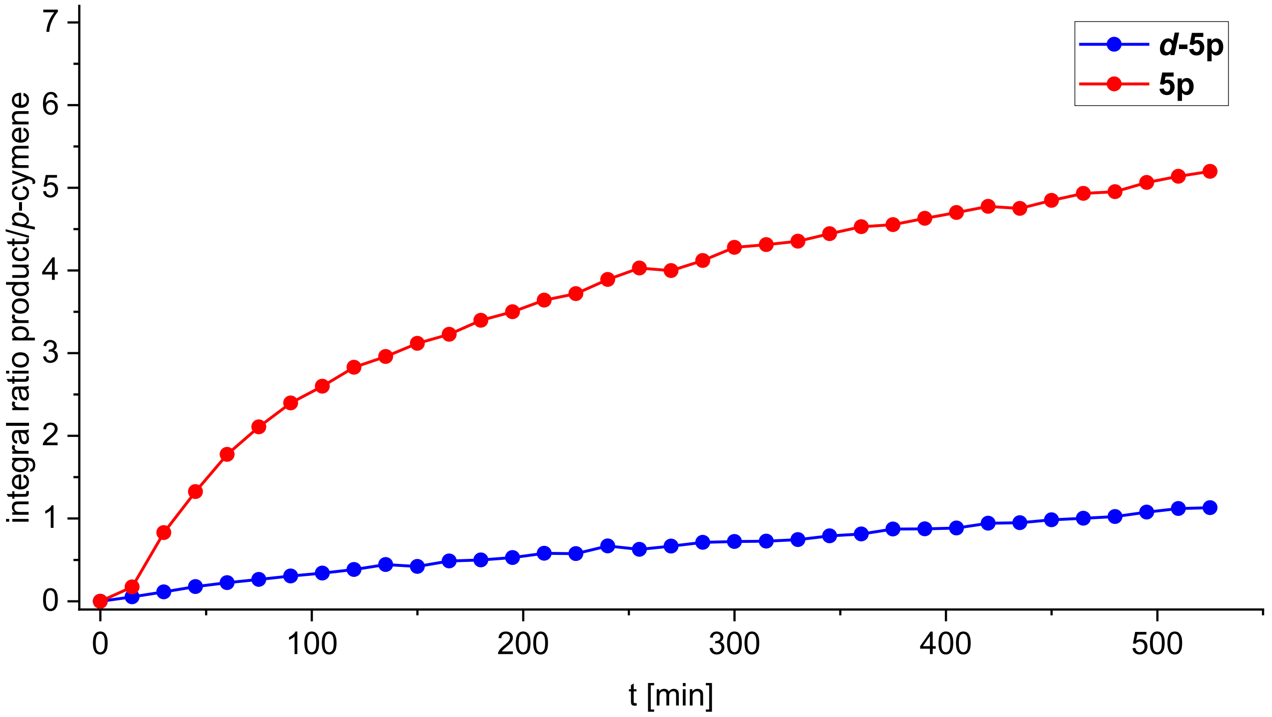

Figure S8: Kinetic investigation of the alkylation of methyl 2-phenylethyl sulfide and methyl-d3-2-phenylethyl sulfide with 1-octene (**2a**) in the presence of complex **Ti2** and ligand precursor **LH1**.

6. NMR Spectra

Tetrakis((trimethylsilyl)methyl)titanium (Ti2)[4]

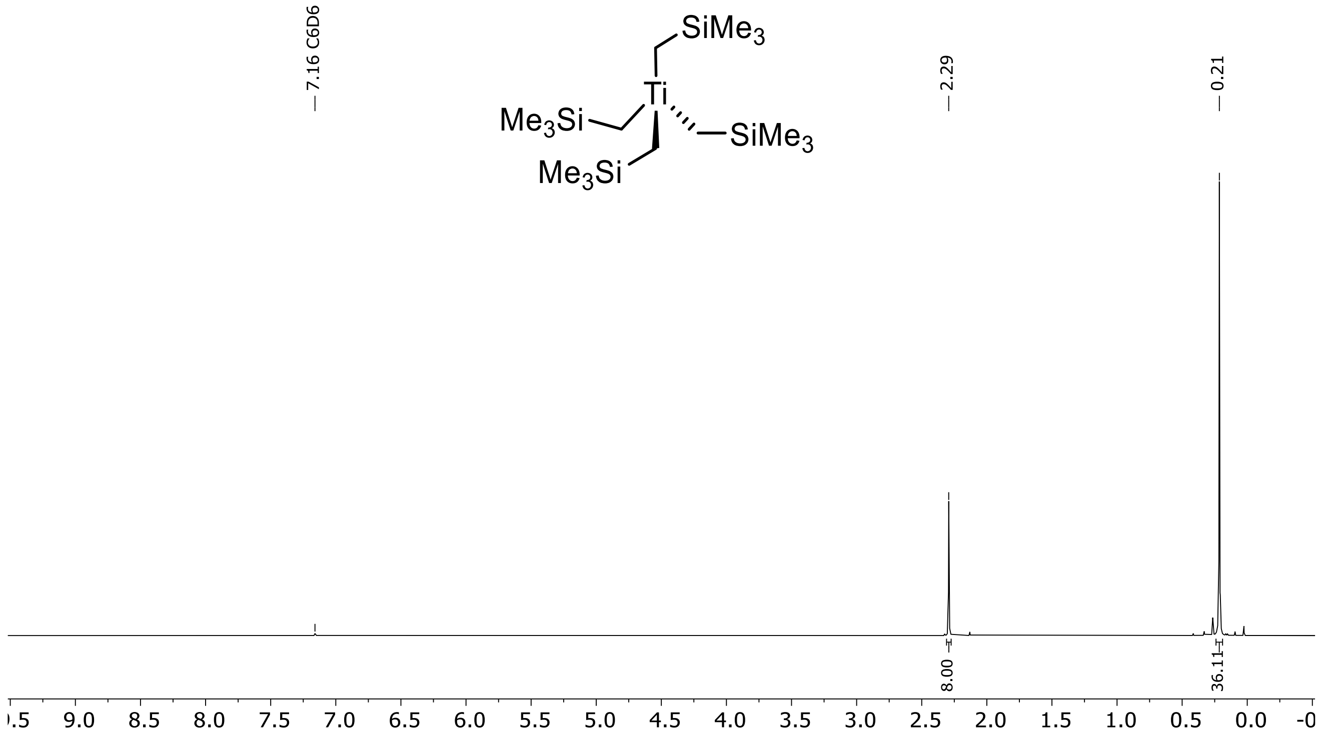

^{1}H NMR spectrum (500 MHz, 305 K, C_6D_6, x-axis in ppm)

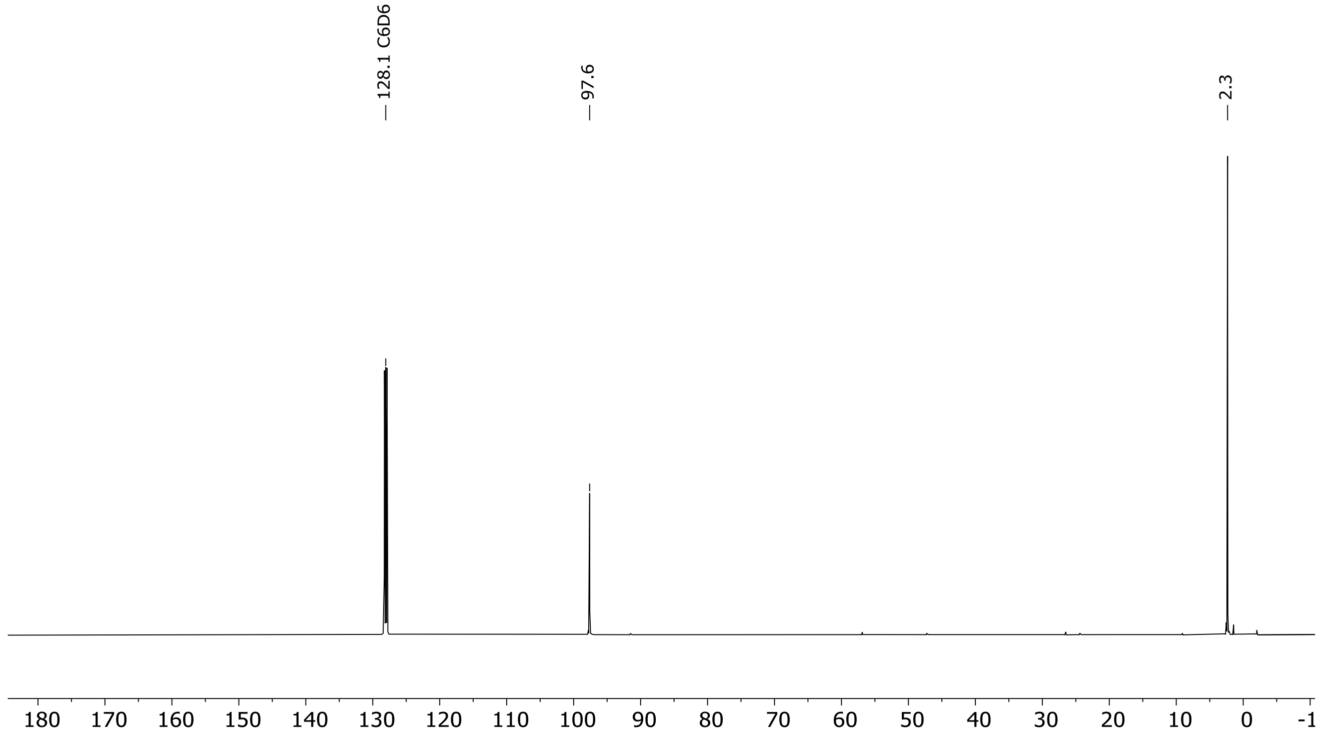

^{13}C{^{1}H} NMR spectrum (125 MHz, 305 K, C_6D_6, x-axis in ppm)

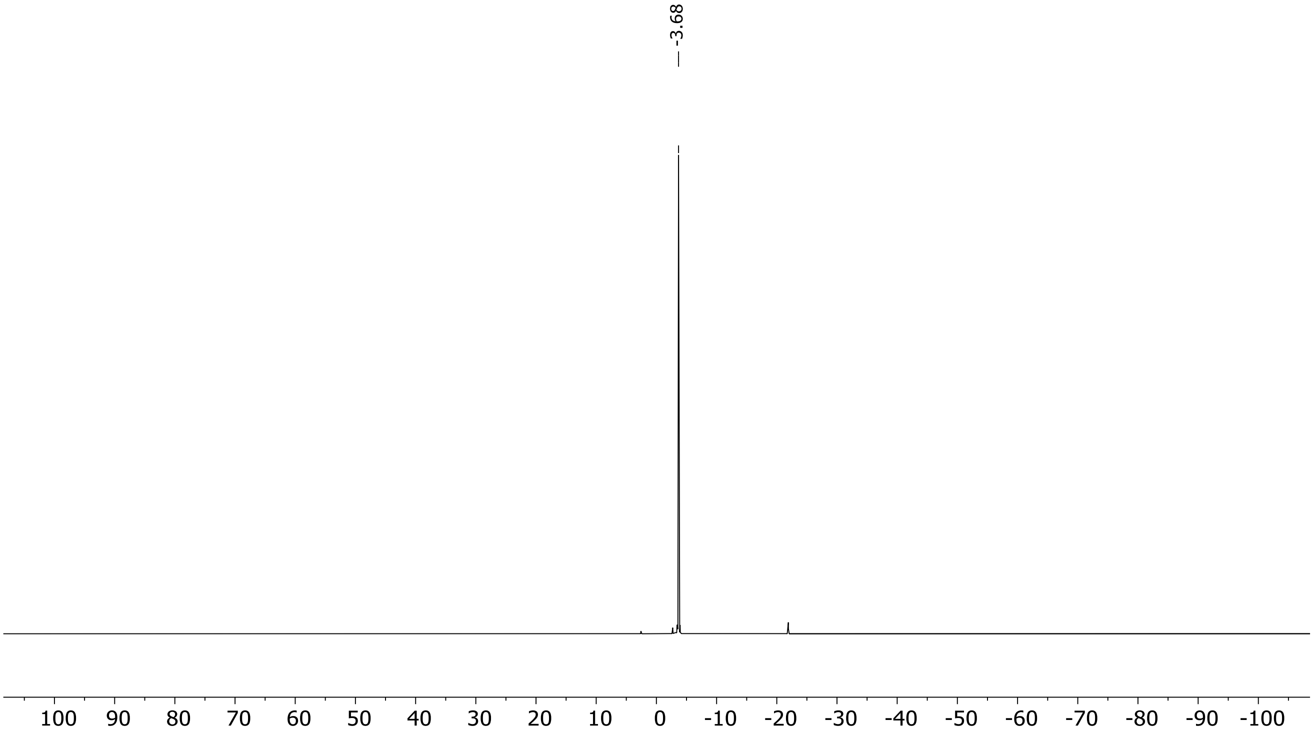

$^{29}Si\{^{1}H\}$ INEPT NMR spectrum (99 MHz, 305 K, C_6D_6, x-axis in ppm)

2-Methyl-1-(pentylthio)octane (5a)[5]

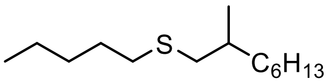

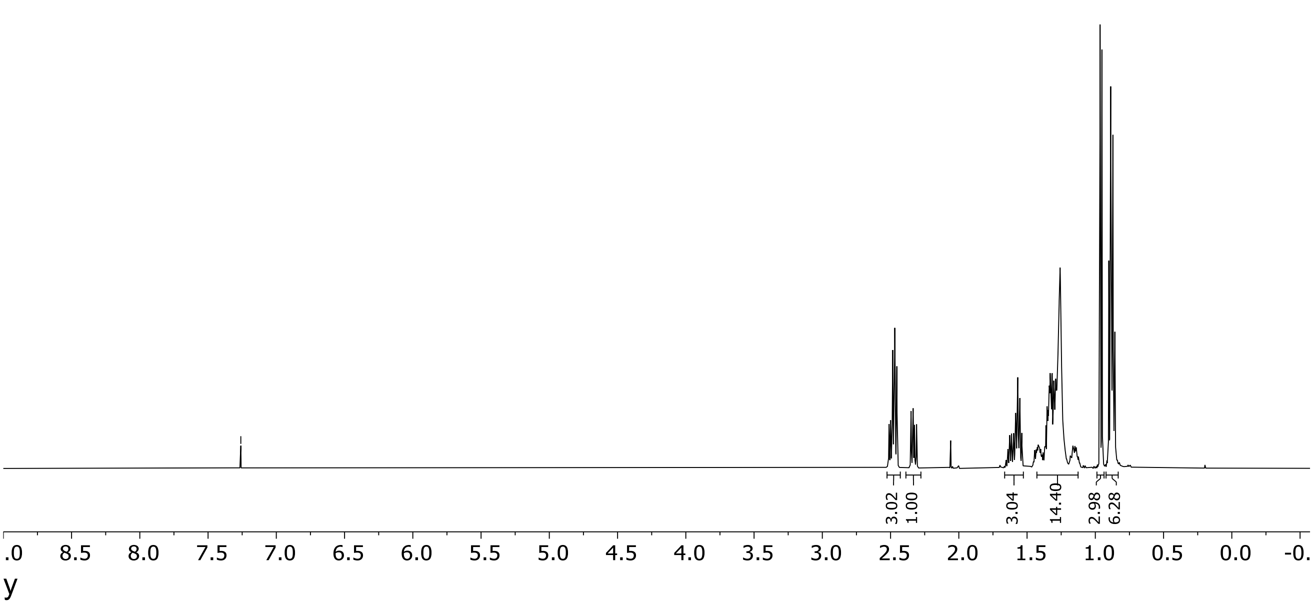

^{1}H NMR spectrum (500 MHz, 305 K, $CDCl_3$, x-axis in ppm)

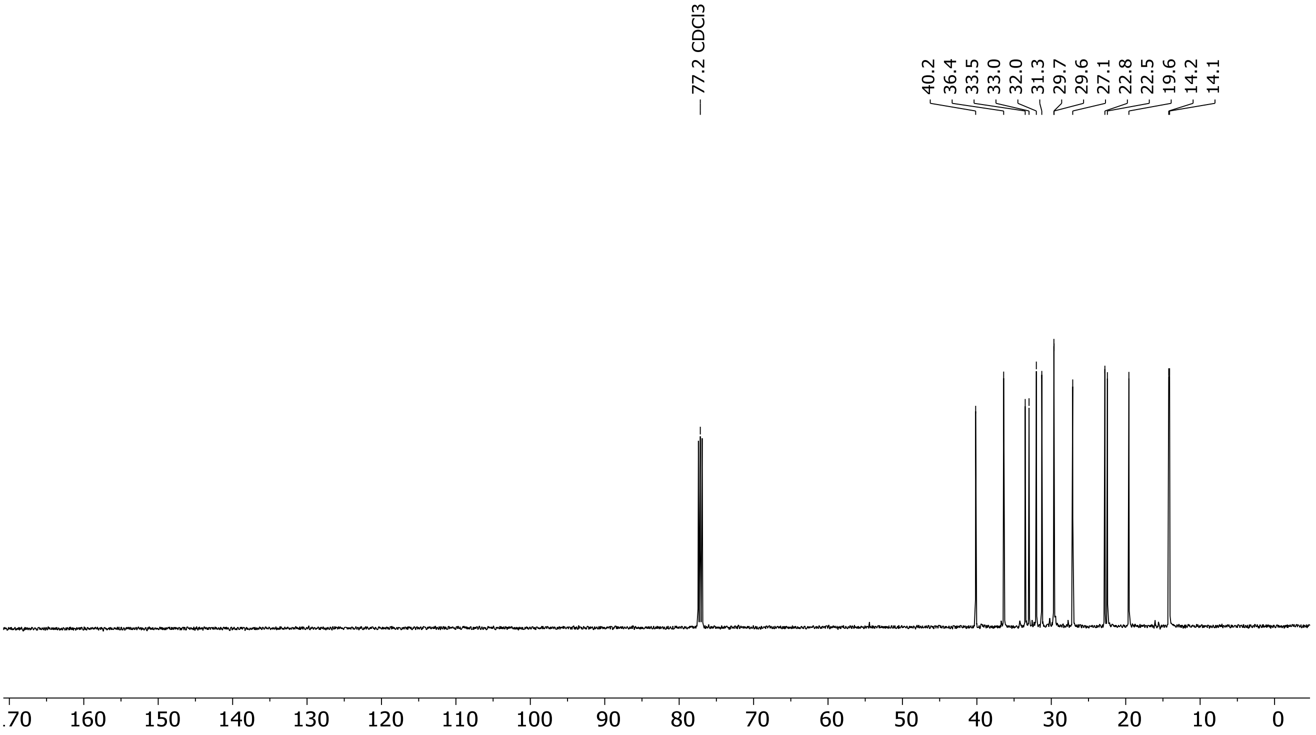

^{13}C{^{1}H} NMR spectrum (125 MHz, 305 K, $CDCl_3$, x-axis in ppm)

1-[(4-Phenyl-2-methylbutyl)thio]pentane (5b)[5]

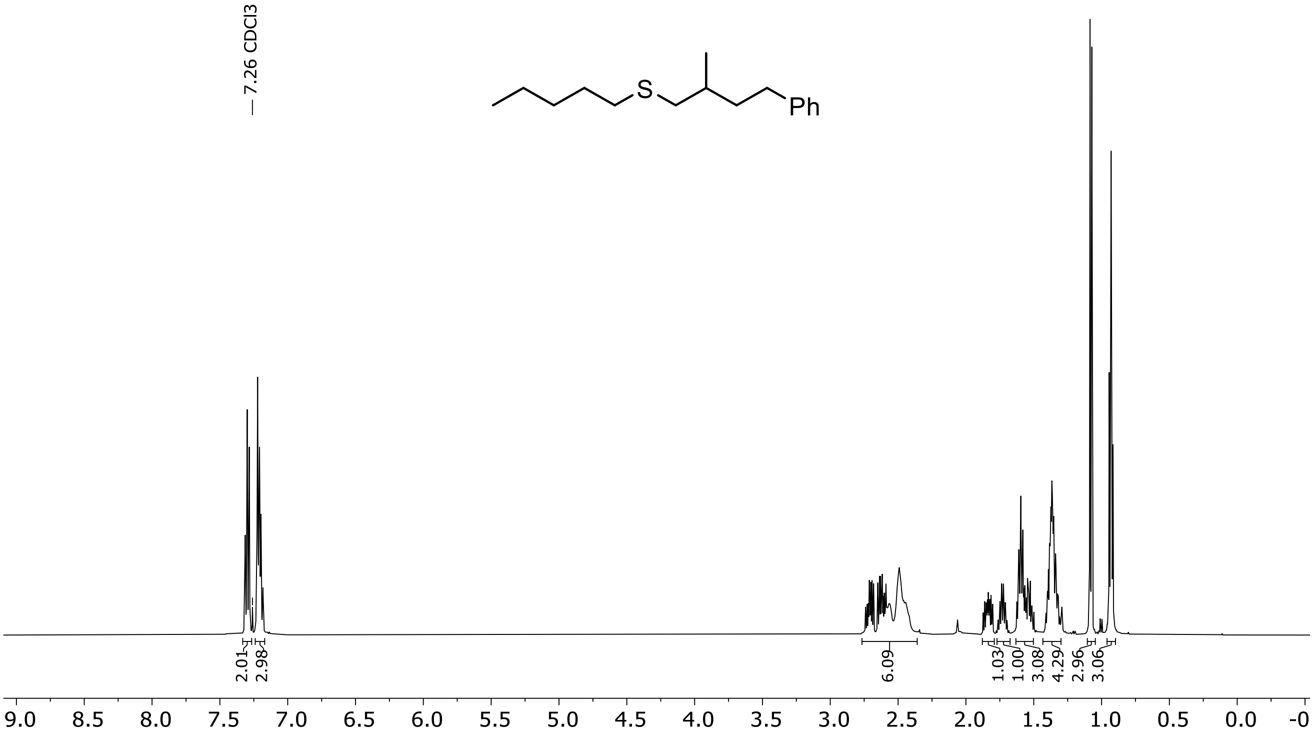

^{1}H NMR spectrum (500 MHz, 305 K, $CDCl_3$, x-axis in ppm)

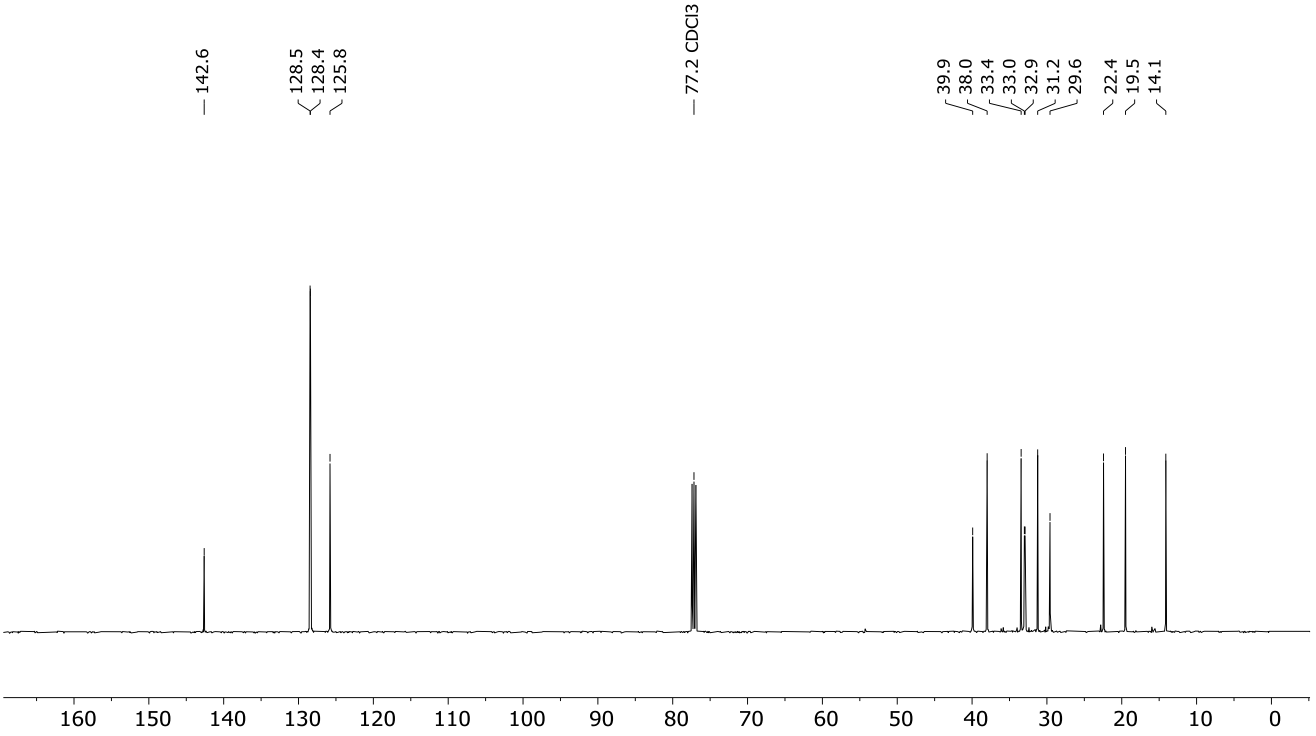

^{13}C{^{1}H} NMR spectrum (125 MHz, 305 K, $CDCl_3$, x-axis in ppm)

1-[(3-Phenyl-2-methylpropyl)thio]pentane (5c)[5]

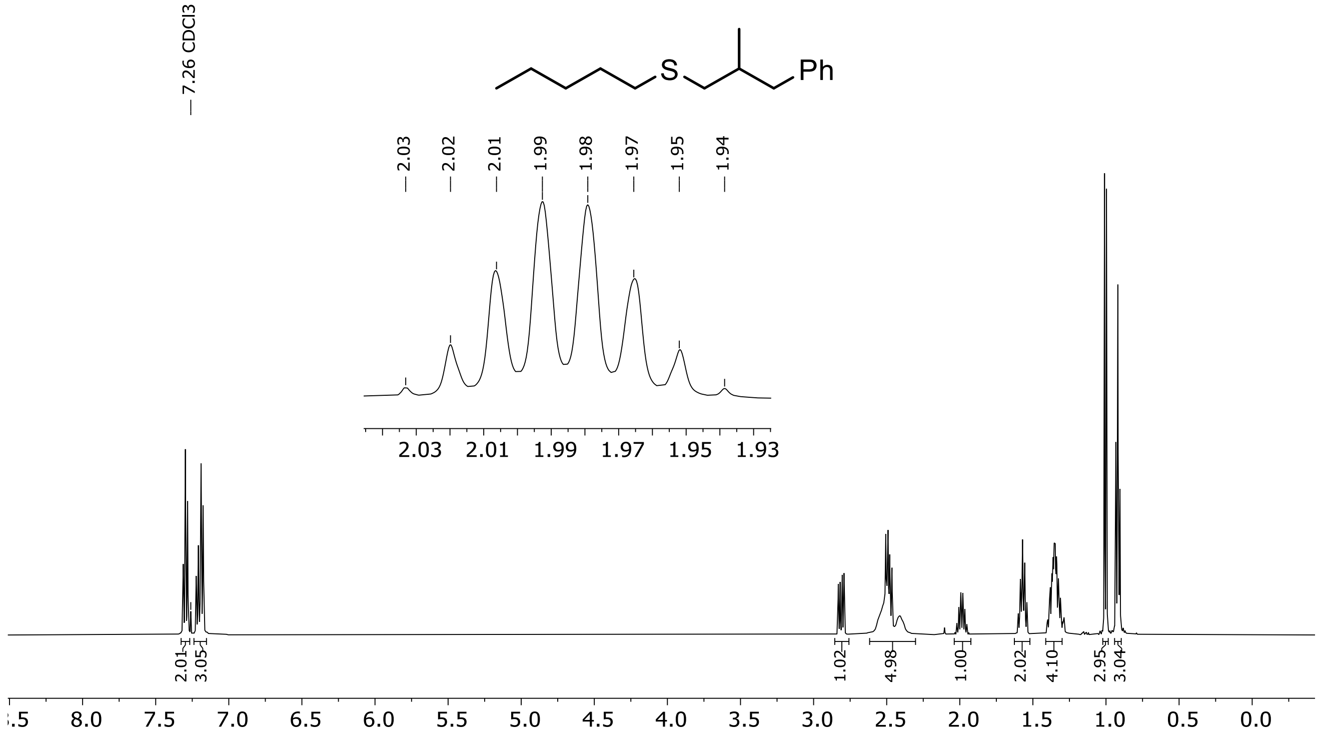

^{1}H NMR spectrum (500 MHz, 305 K, $CDCl_3$, x-axis in ppm)

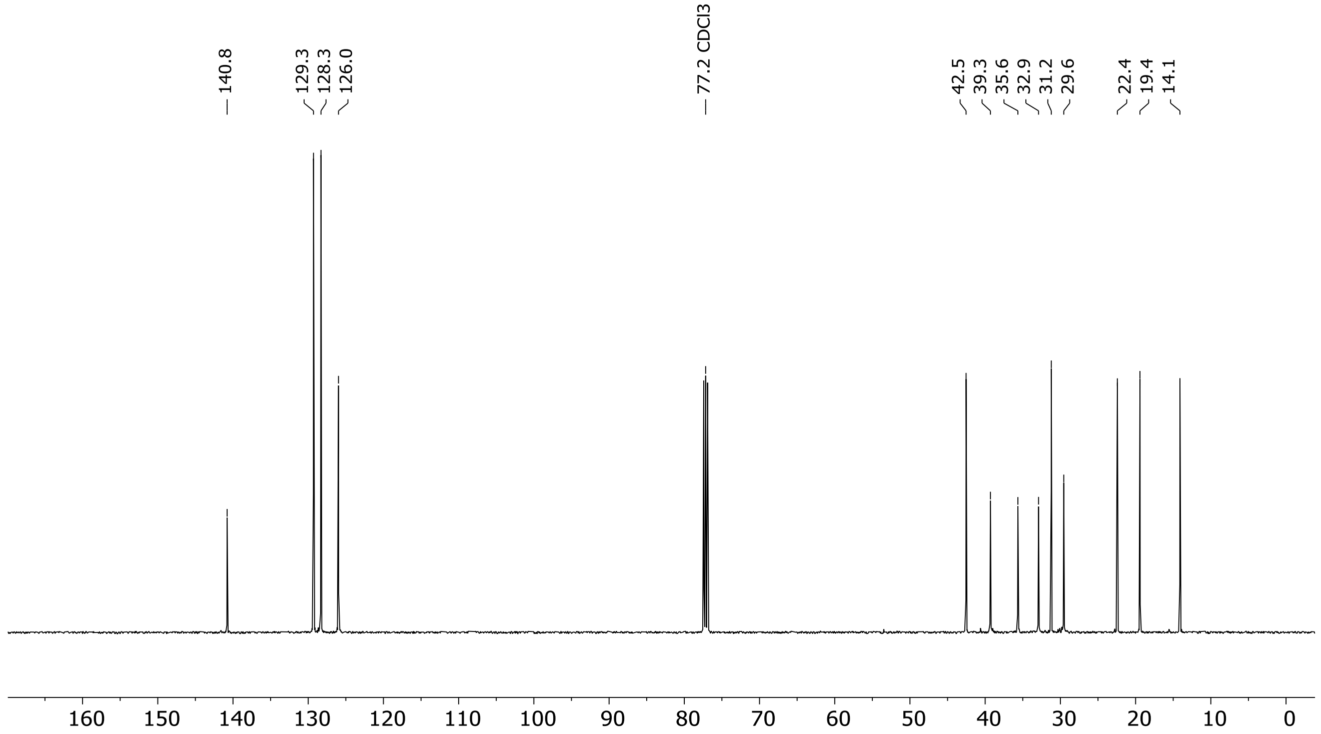

$^{13}C\{^1H\}$ NMR spectrum (125 MHz, 305 K, $CDCl_3$, x-axis in ppm)

1-[(2,3,3-Trimethylbutyl)thio]pentane (5d)

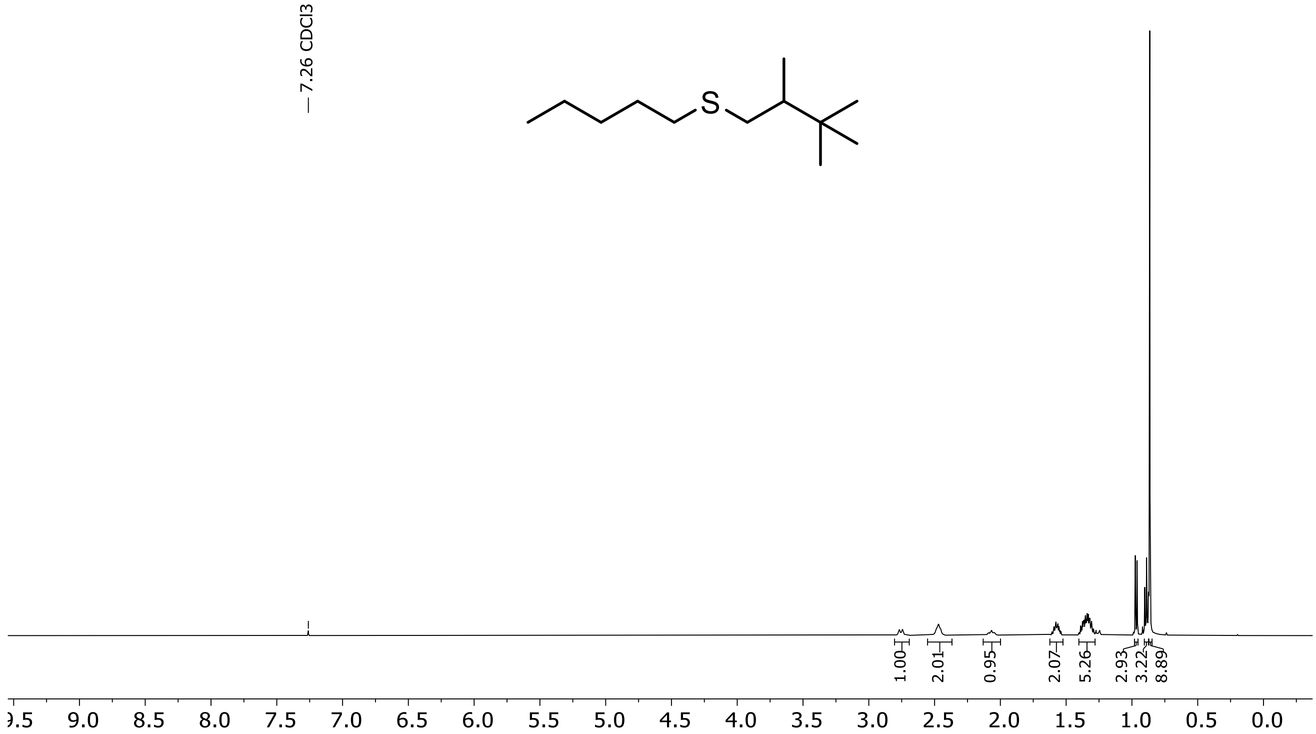

^{1}H NMR spectrum (500 MHz, 305 K, $CDCl_3$, x-axis in ppm)

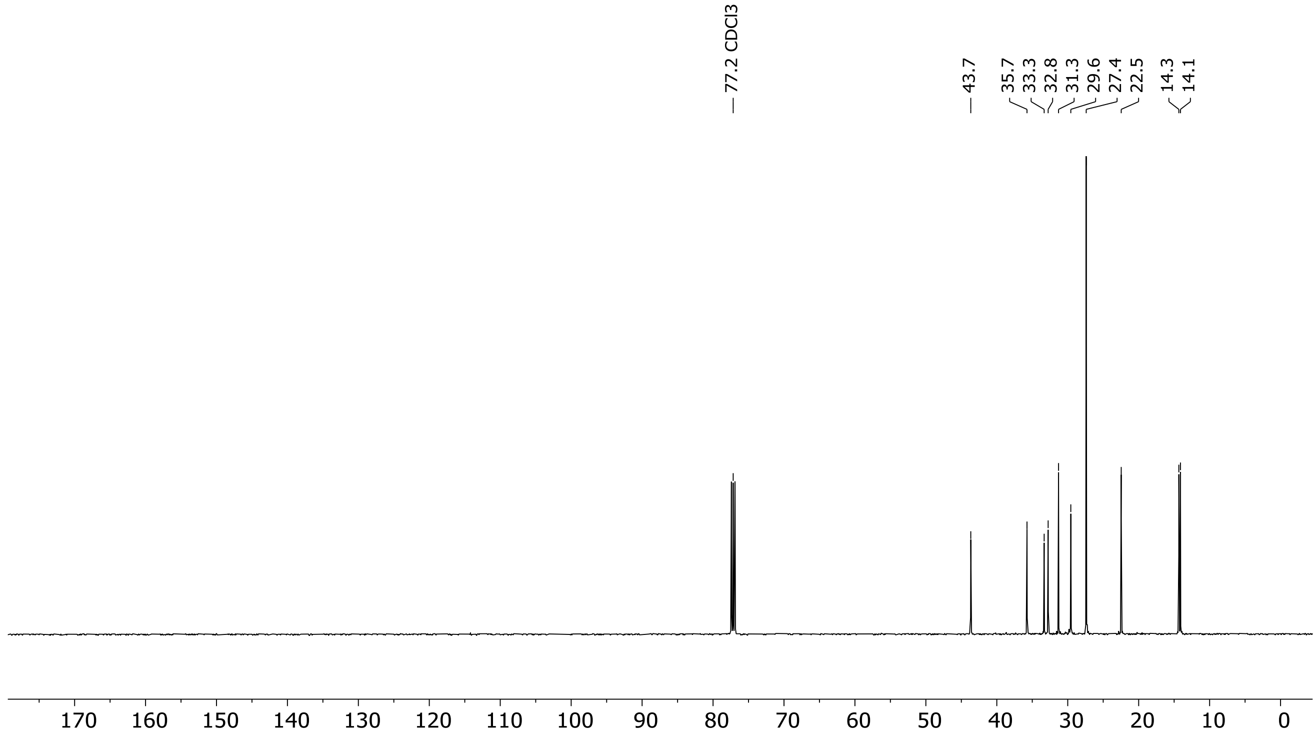

^{13}C{^{1}H} NMR spectrum (125 MHz, 305 K, $CDCl_3$, x-axis in ppm)

1-[(2-Methyl-8-phenyloctyl)thio]pentane (5e)

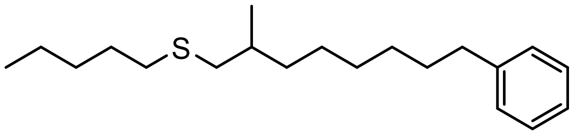

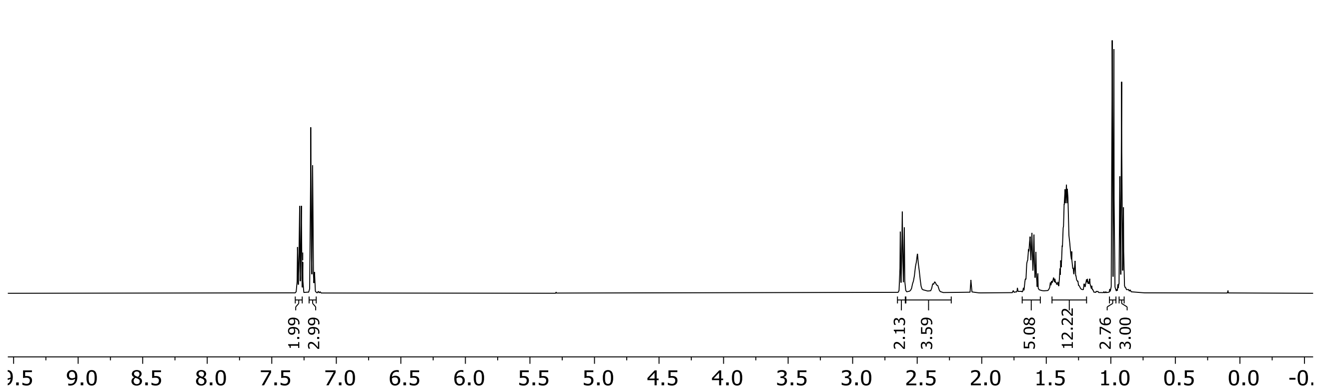

^{1}H NMR spectrum (500 MHz, 305 K, $CDCl_3$, x-axis in ppm)

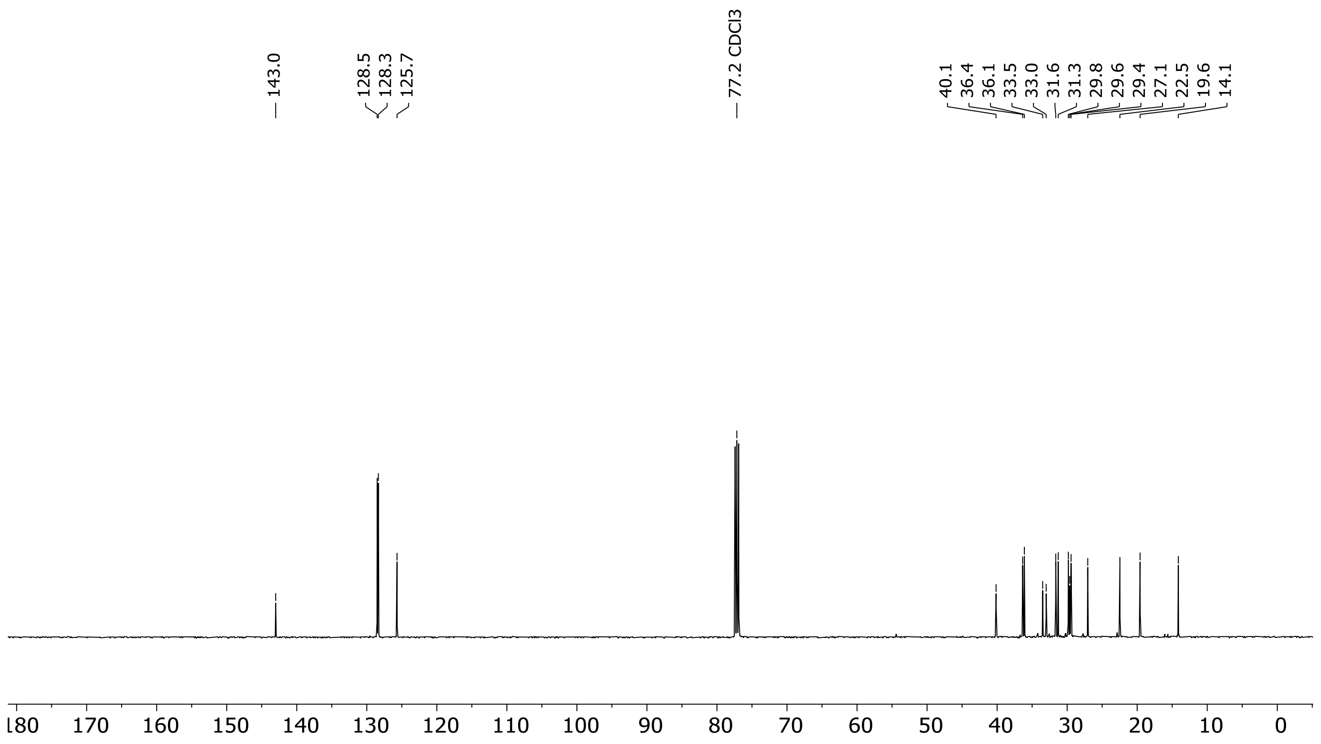

$^{13}C\{^1H\}$ NMR spectrum (125 MHz, 305 K, $CDCl_3$, x-axis in ppm)

Triisopropyl((4-methyl-5-(pentylthio)pentyl)oxy)silane (5f)

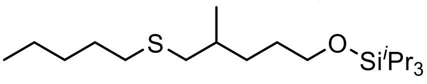

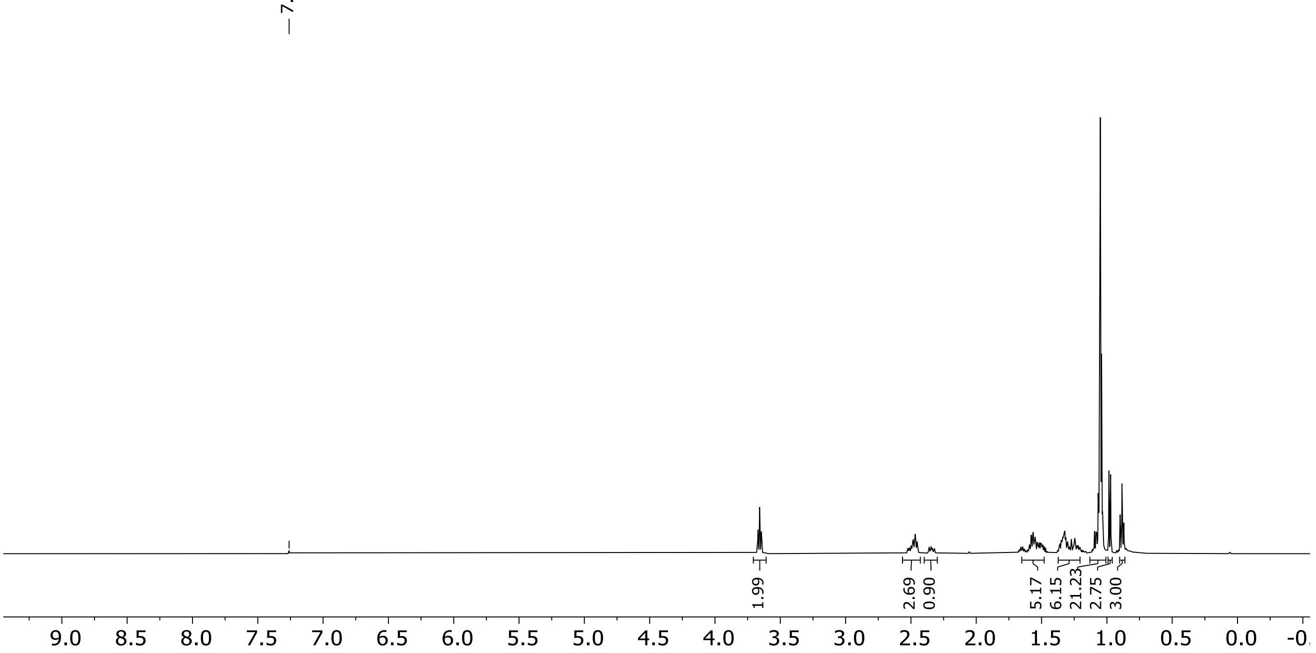

^{1}H NMR spectrum (500 MHz, 305 K, $CDCl_3$, x-axis in ppm)

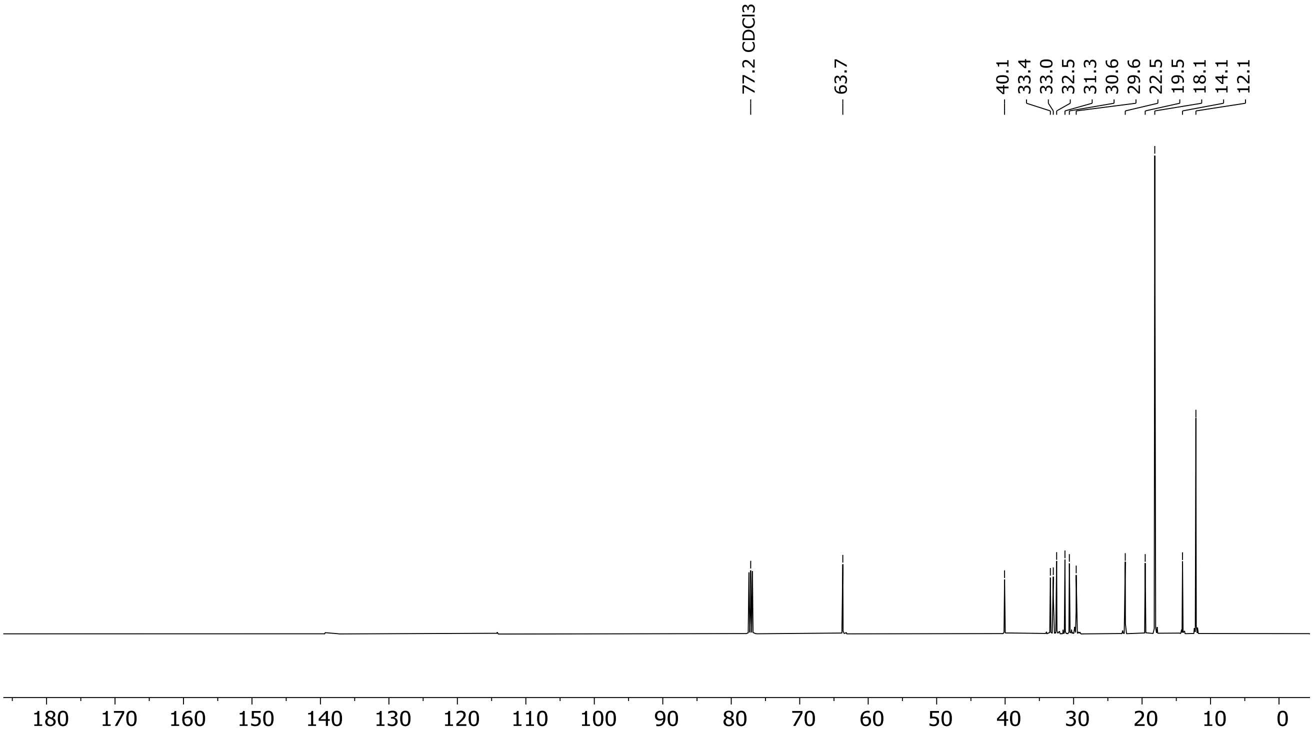

^{13}C{^{1}H} NMR spectrum (125 MHz, 305 K, $CDCl_3$, x-axis in ppm)

1-[(4,4-Diphenyl-2-methylbutyl)thio]pentane (5g)

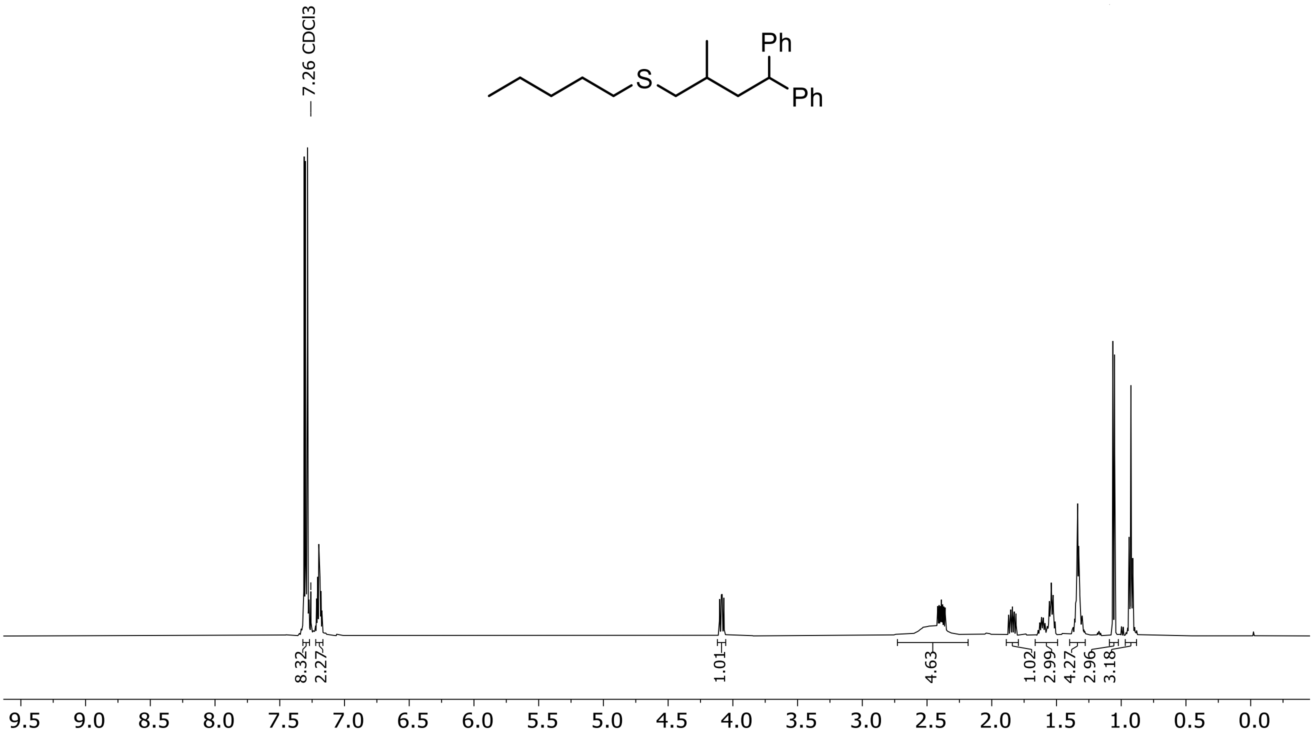

^{1}H NMR spectrum (500 MHz, 305 K, $CDCl_3$, x-axis in ppm)

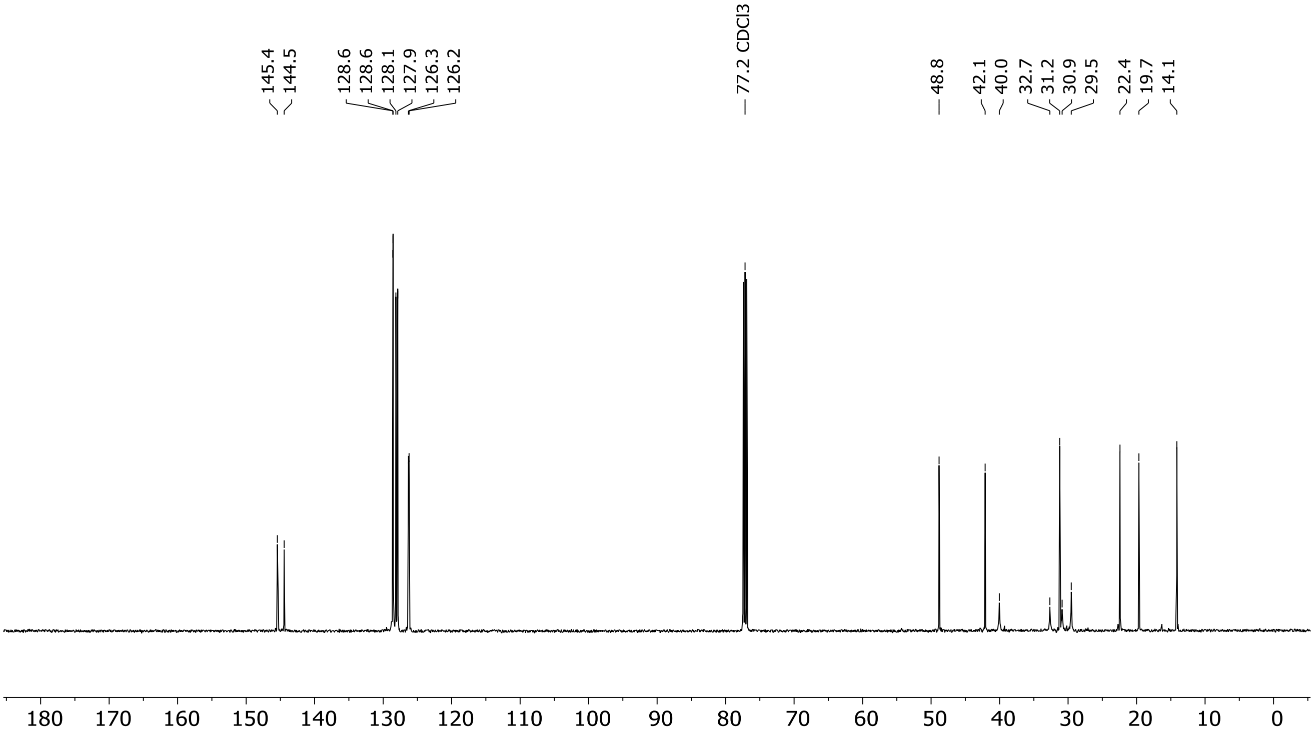

^{13}C{^{1}H} NMR spectrum (125 MHz, 305 K, $CDCl_3$, x-axis in ppm)

1-[(2-Methyl-3-(1,2,3,4-tetrahydronaphthalen-1-yl)propyl)thio]pentane (5h)

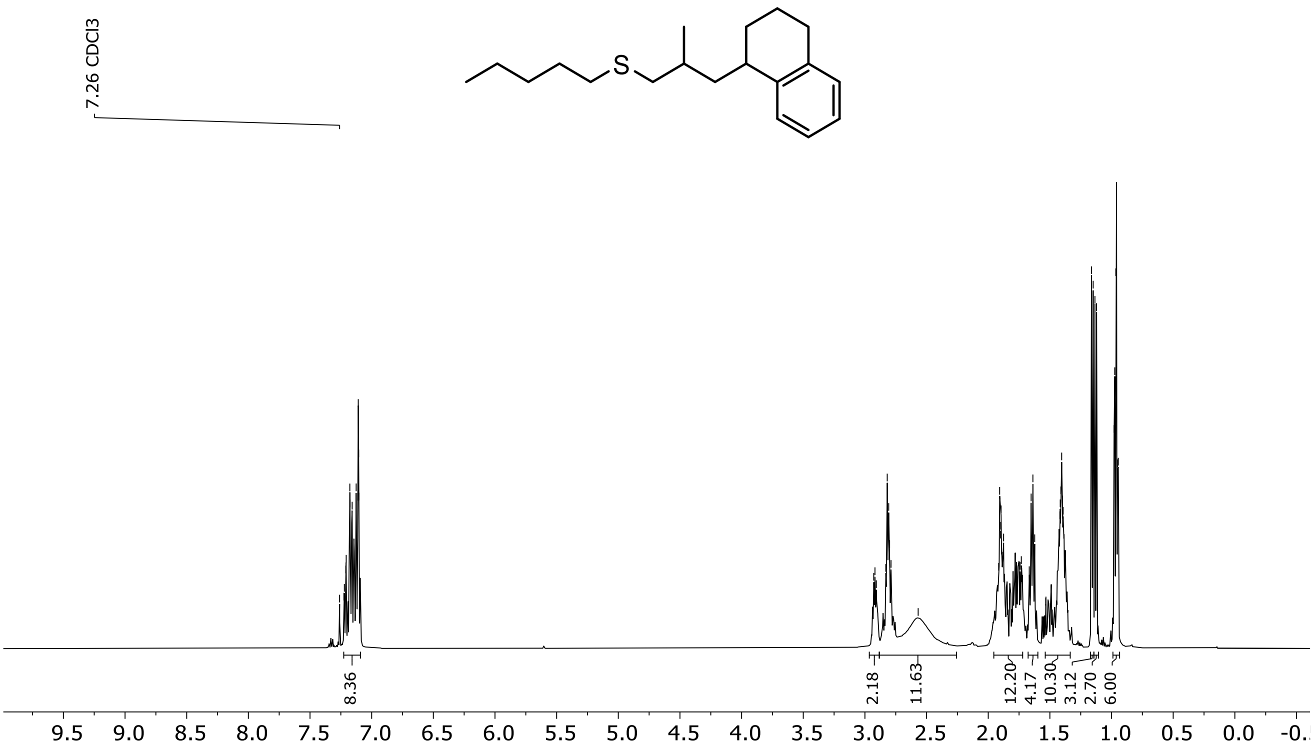

^{1}H NMR spectrum (500 MHz, 305 K, $CDCl_3$, x-axis in ppm)

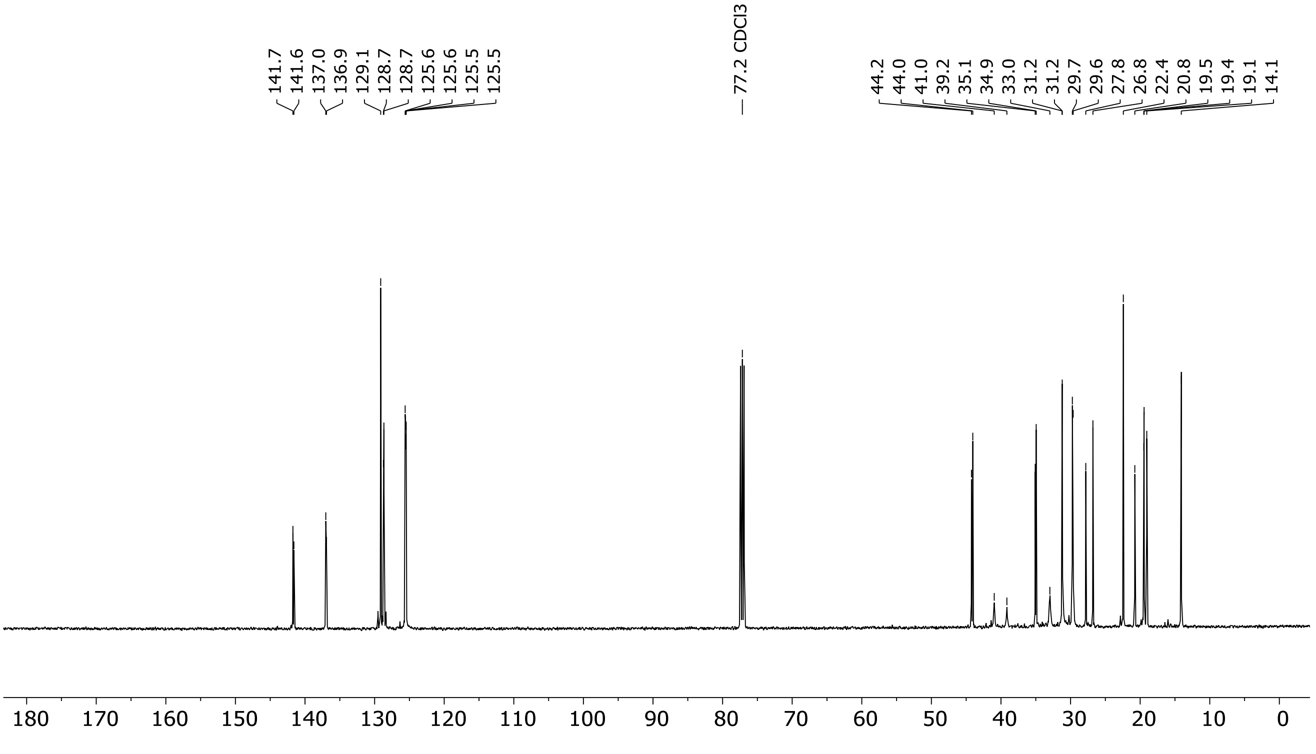

$^{13}C\{^1H\}$ NMR spectrum (125 MHz, 305 K, $CDCl_3$, x-axis in ppm)

1-[(Cyclopentylmethyl)thio]pentane (5i)

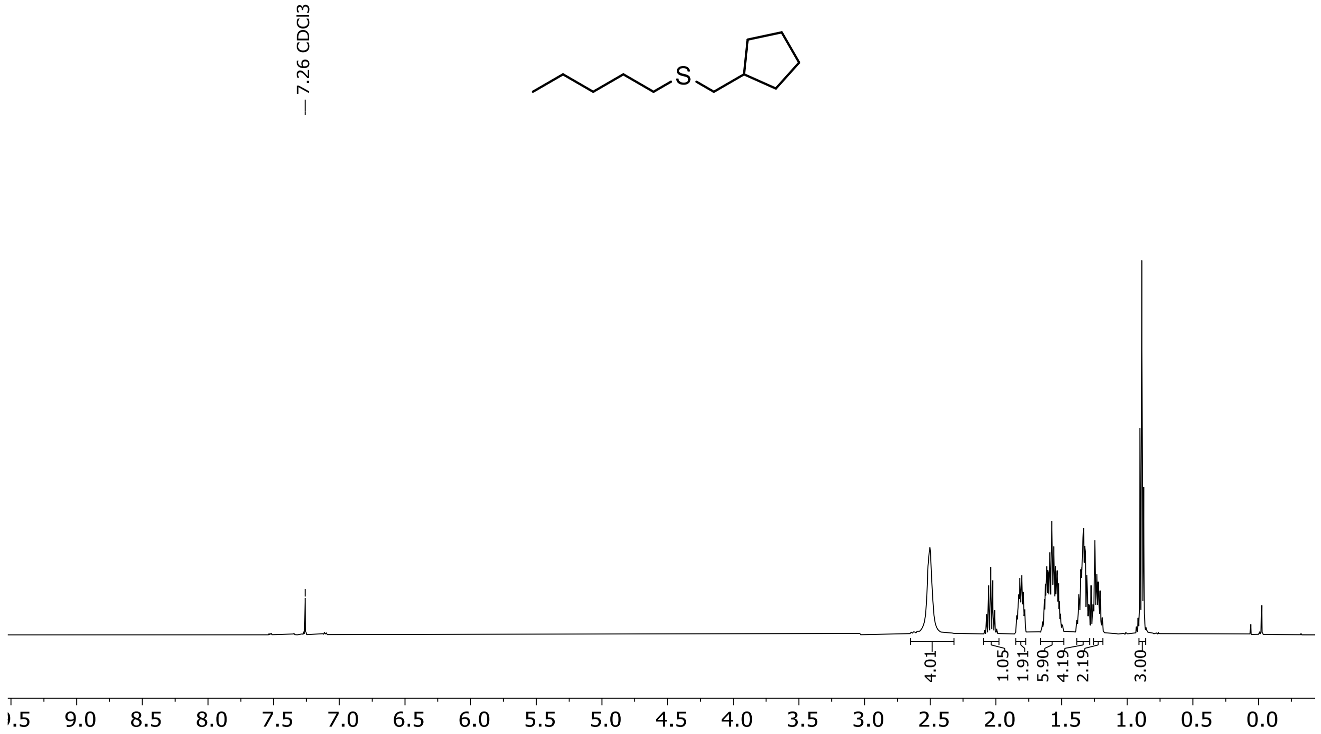

^{1}H NMR spectrum (500 MHz, 305 K, $CDCl_3$, x-axis in ppm)

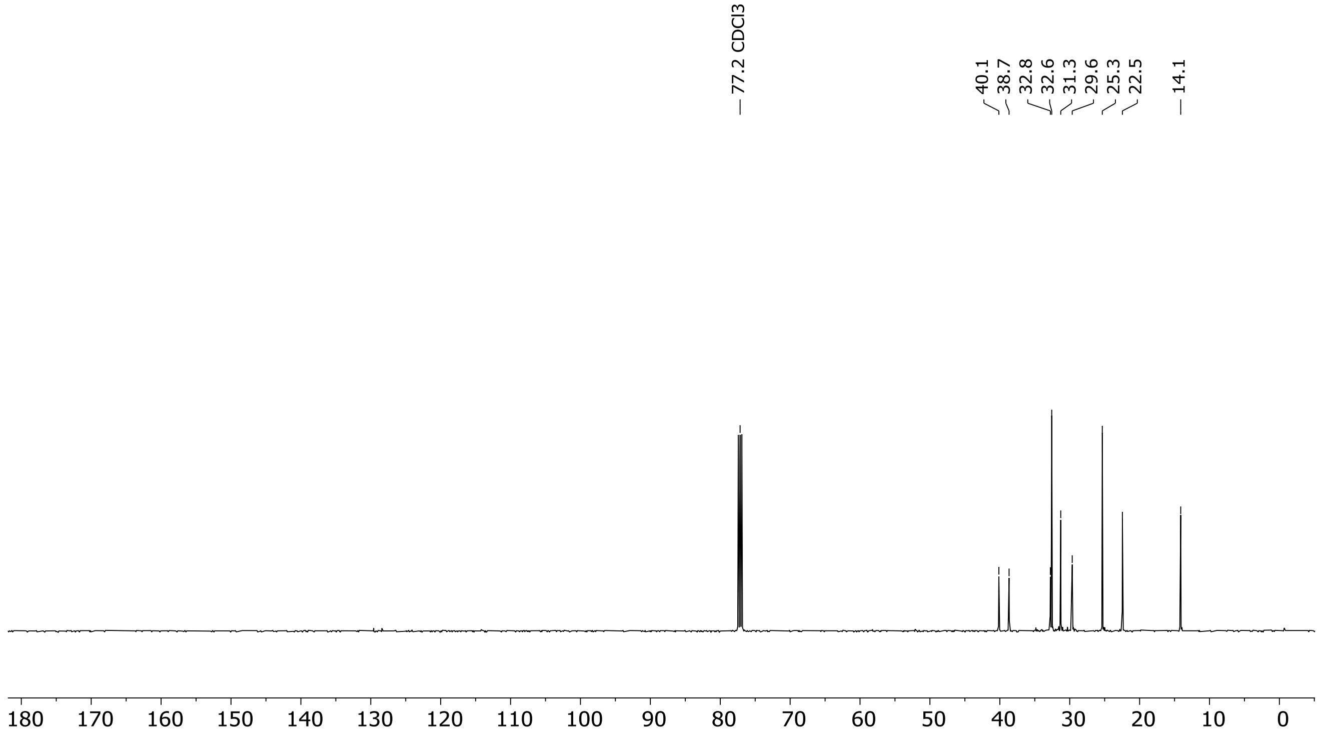

$^{13}C\{^1H\}$ NMR spectrum (125 MHz, 305 K, $CDCl_3$, x-axis in ppm)

1-[(Cyclohexylmethyl)thio]pentane (5j)

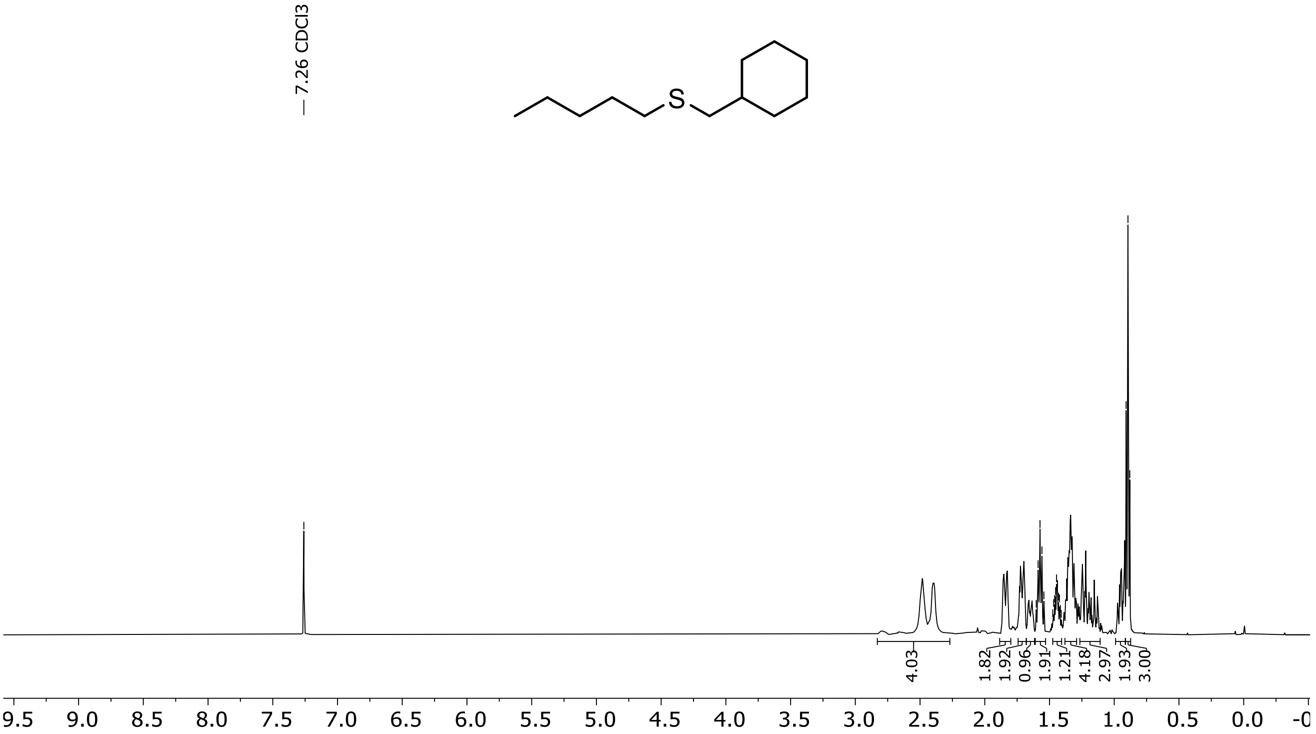

^{1}H NMR spectrum (500 MHz, 305 K, $CDCl_3$, x-axis in ppm)

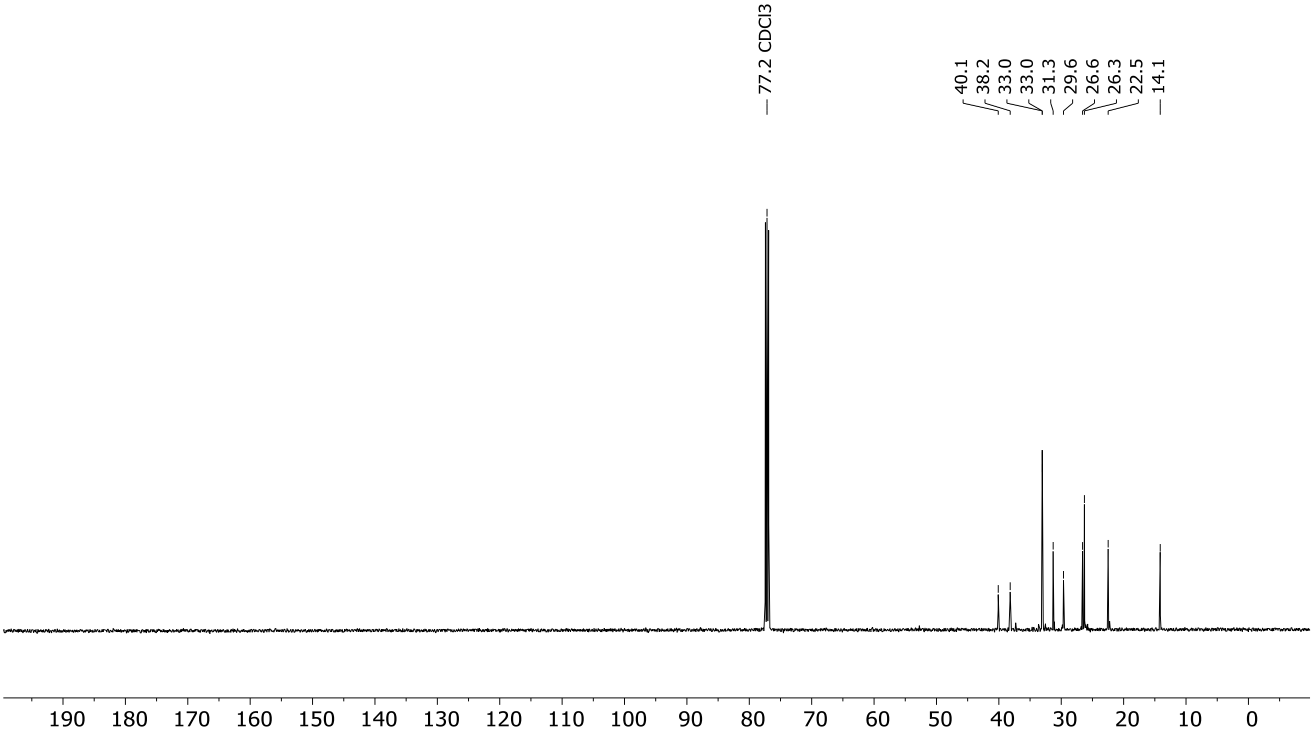

^{13}C{^{1}H} NMR spectrum (125 MHz, 305 K, $CDCl_3$, x-axis in ppm)

[2-(Cyclohex-3-en-1-yl)propylthio]pentane (5k)

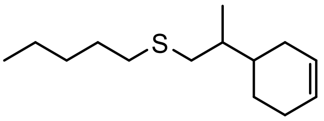

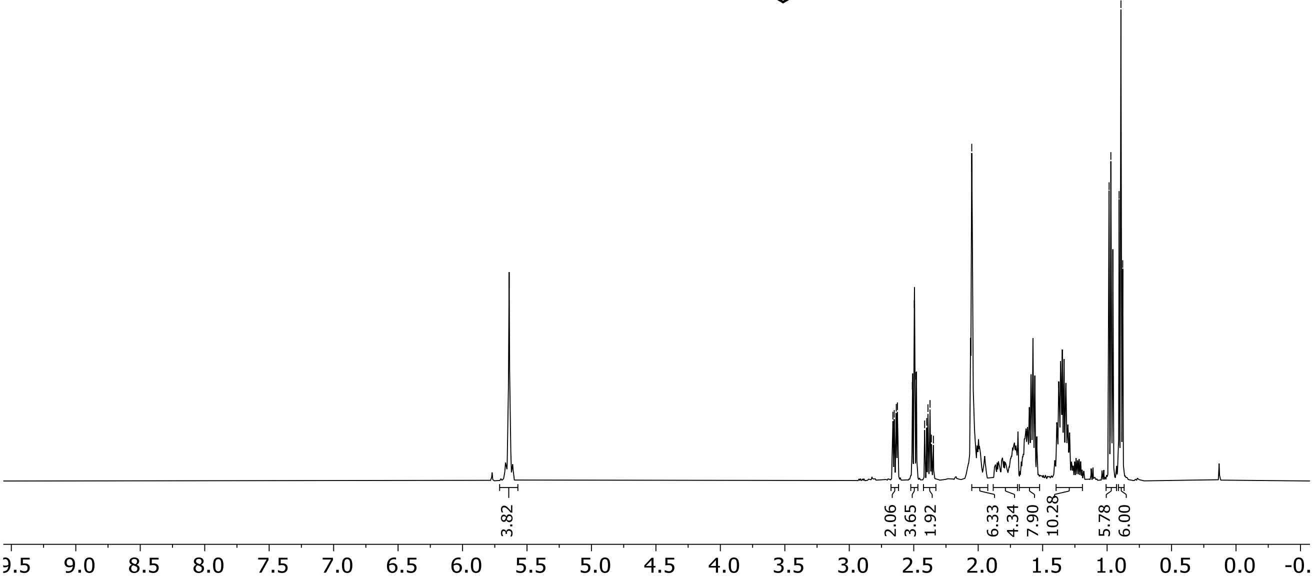

^{1}H NMR spectrum (500 MHz, 305 K, $(CD_3)_2CO$, x-axis in ppm)

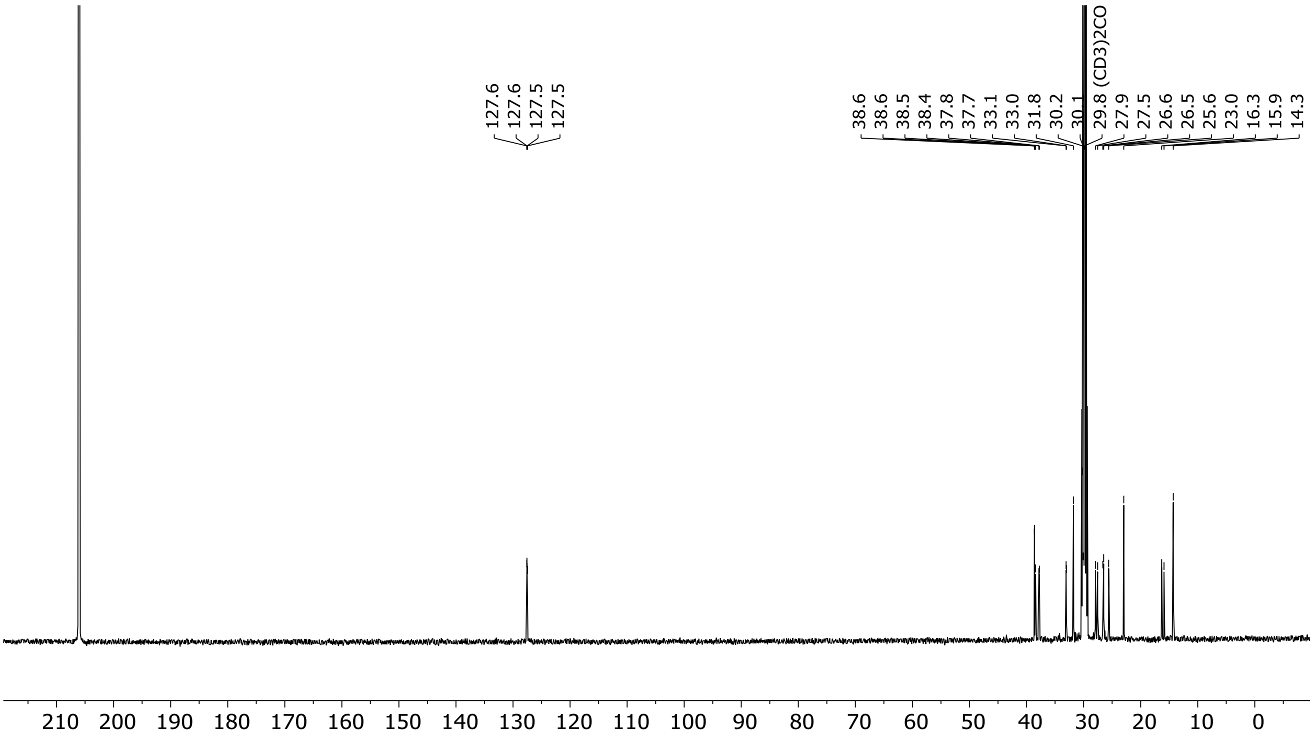

^{13}C{^{1}H} NMR spectrum (125 MHz, 305 K, $(CD_3)_2CO$, x-axis in ppm)

1-[(*E*)-(2-Methyltridec-11-en-1-yl)thio]pentane (5l)

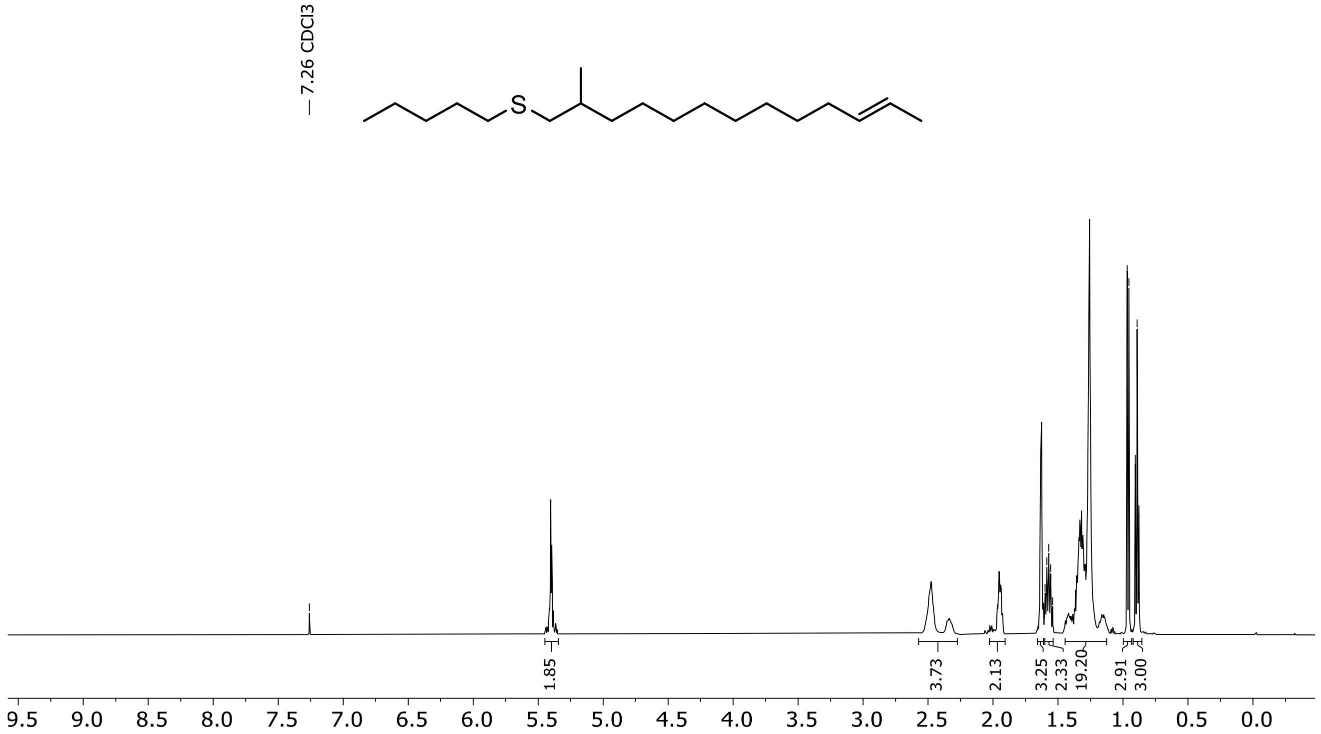

^{1}H NMR spectrum (500 MHz, 305 K, $CDCl_3$, x-axis in ppm)

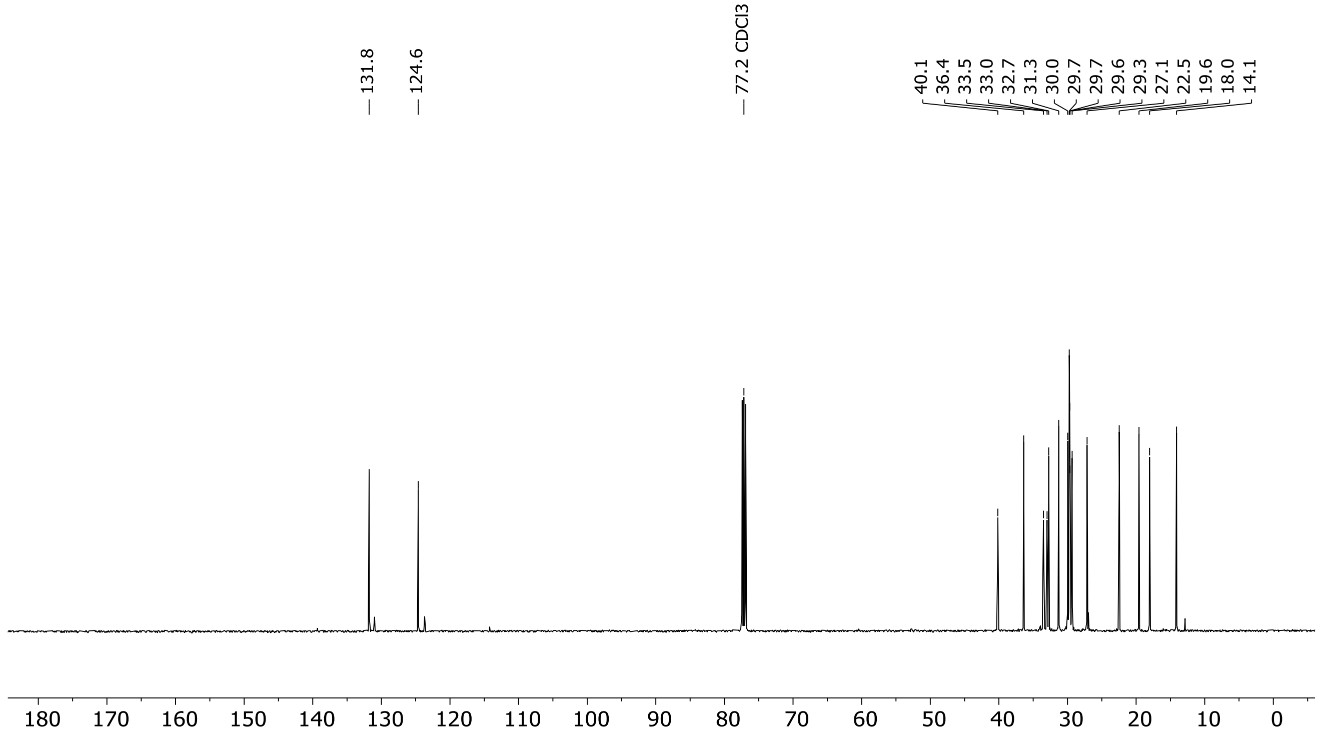

$^{13}C\{^{1}H\}$ NMR spectrum (125 MHz, 305 K, $CDCl_3$, x-axis in ppm)

1-[(4-(2-Bromophenyl)-2-methylbutyl)thio]pentane (5m)

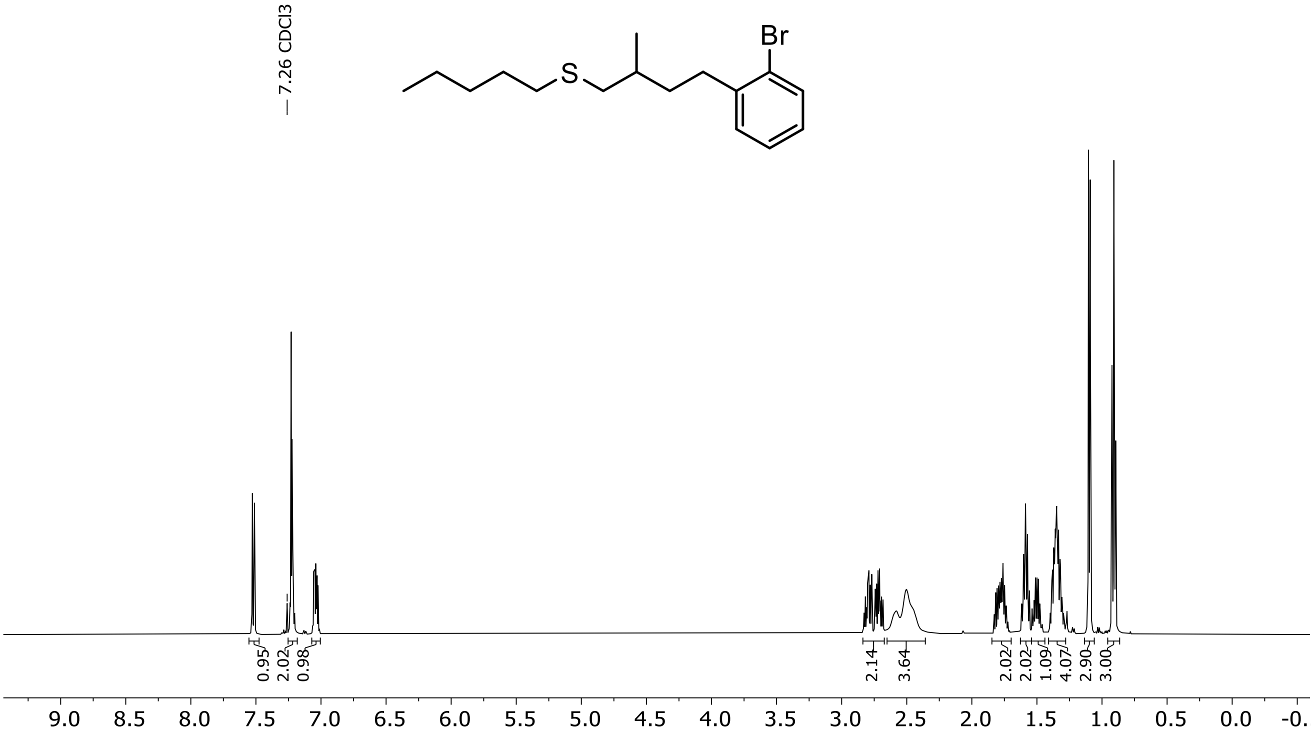

^{1}H NMR spectrum (500 MHz, 305 K, $CDCl_3$, x-axis in ppm)

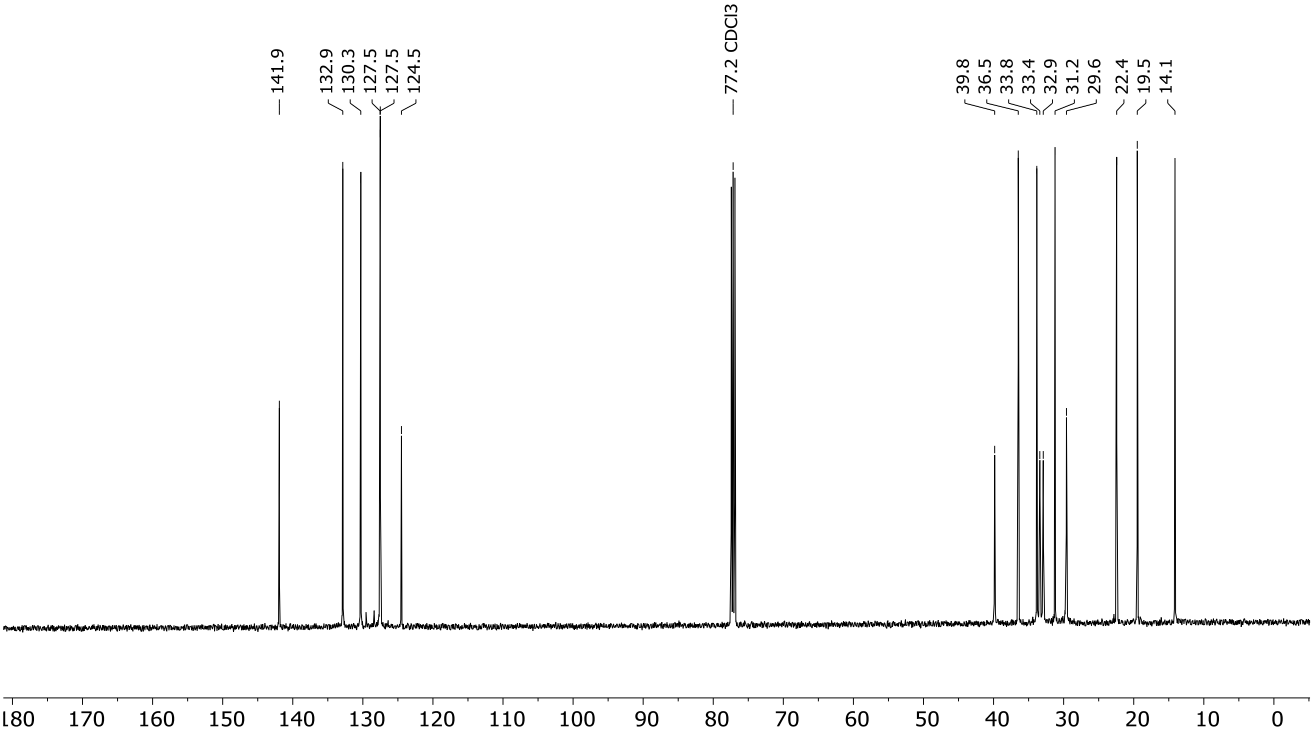

^{13}C{^{1}H} NMR spectrum (125 MHz, 305 K, $CDCl_3$, x-axis in ppm)

1-[(4-(4-Chlorophenyl)-2-methylbutyl)thio]pentane (5n)

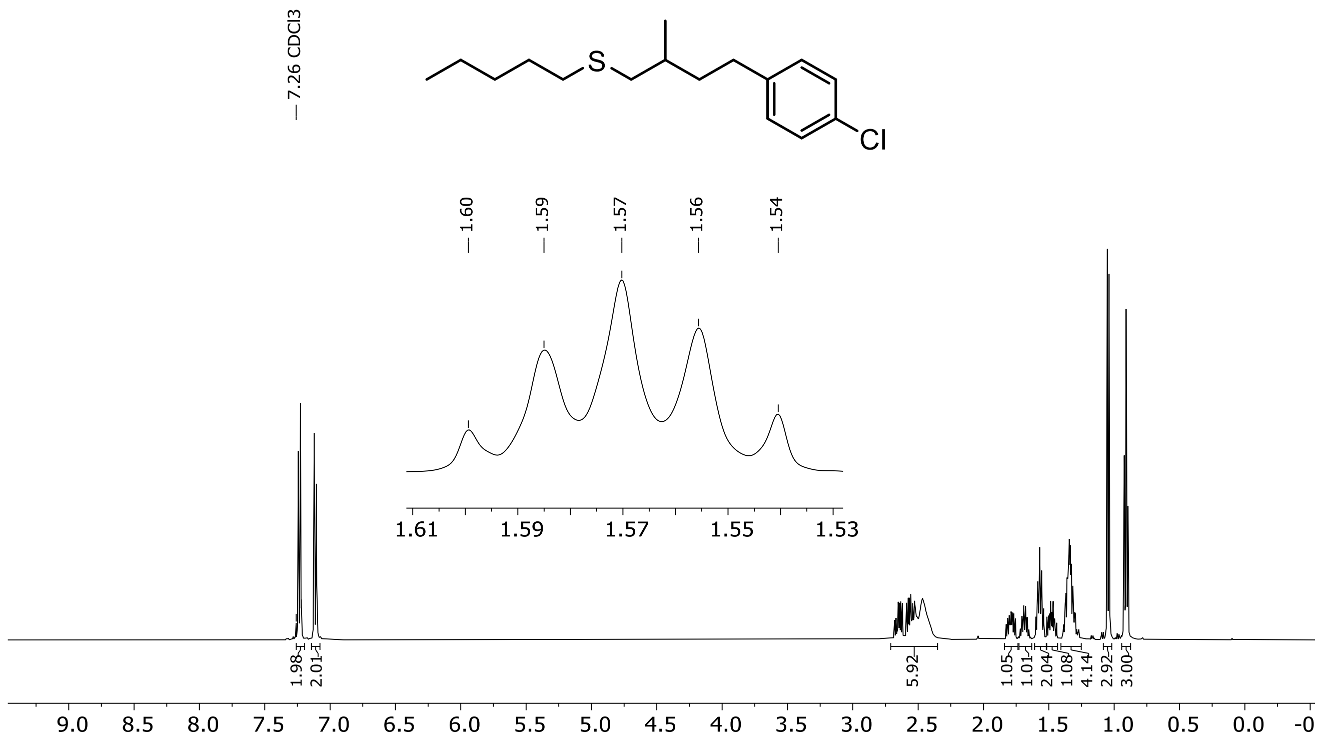

^{1}H NMR spectrum (500 MHz, 305 K, $CDCl_3$, x-axis in ppm)

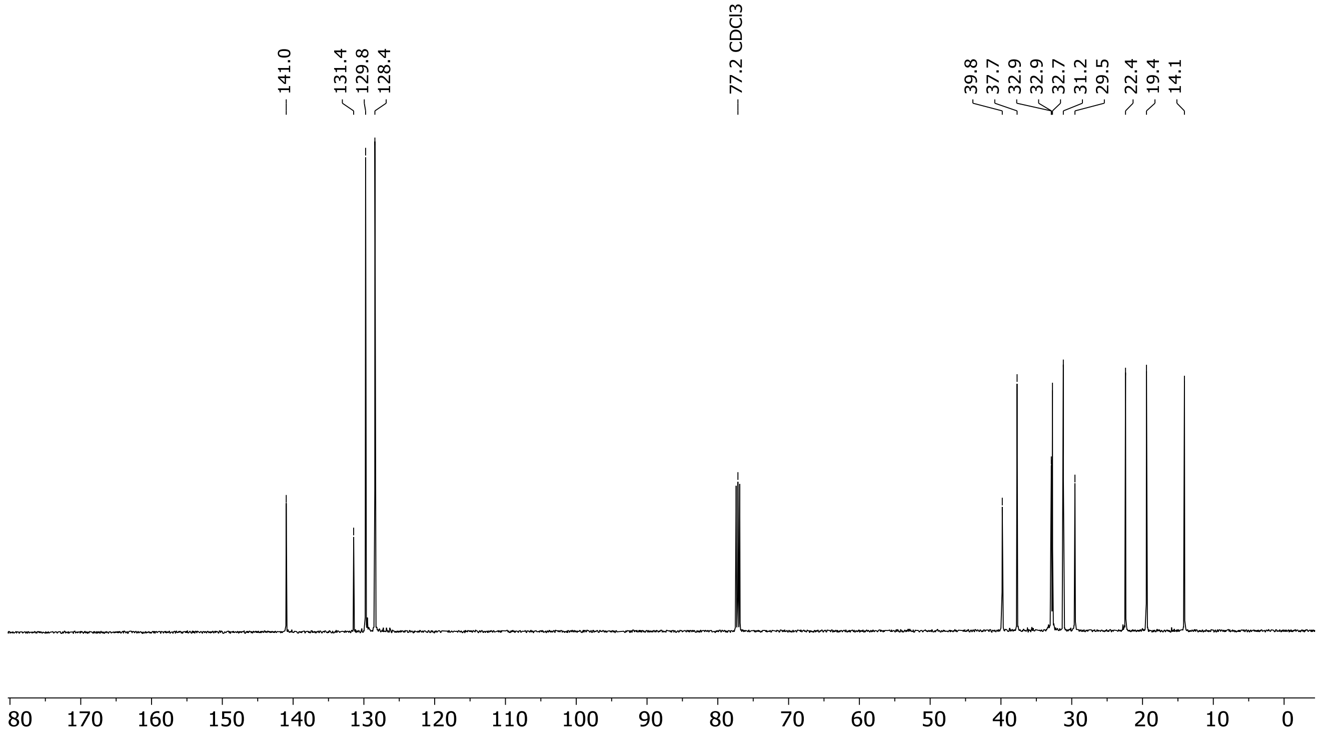

^{13}C{^{1}H} NMR spectrum (125 MHz, 305 K, $CDCl_3$, x-axis in ppm)

1-[(2-Methyloctyl)thio]decane (5o)

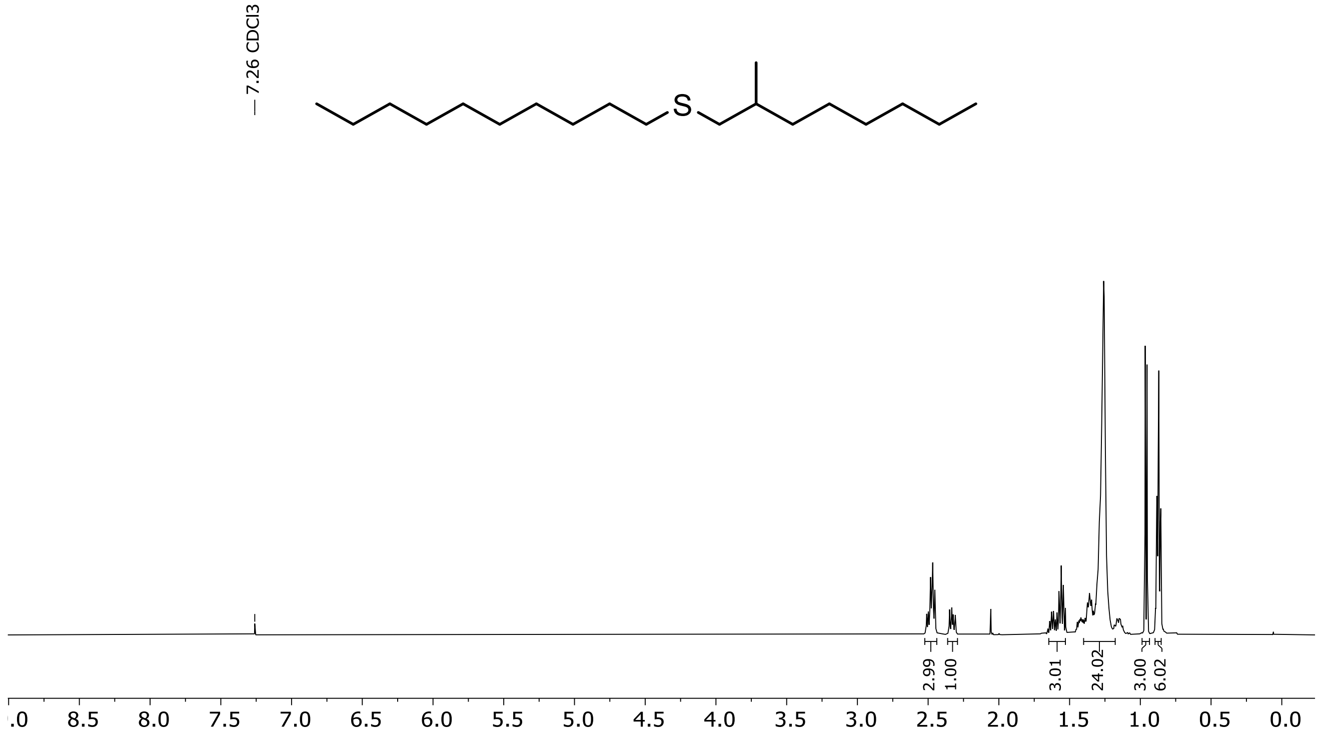

^{1}H NMR spectrum (500 MHz, 305 K, $CDCl_3$, x-axis in ppm)

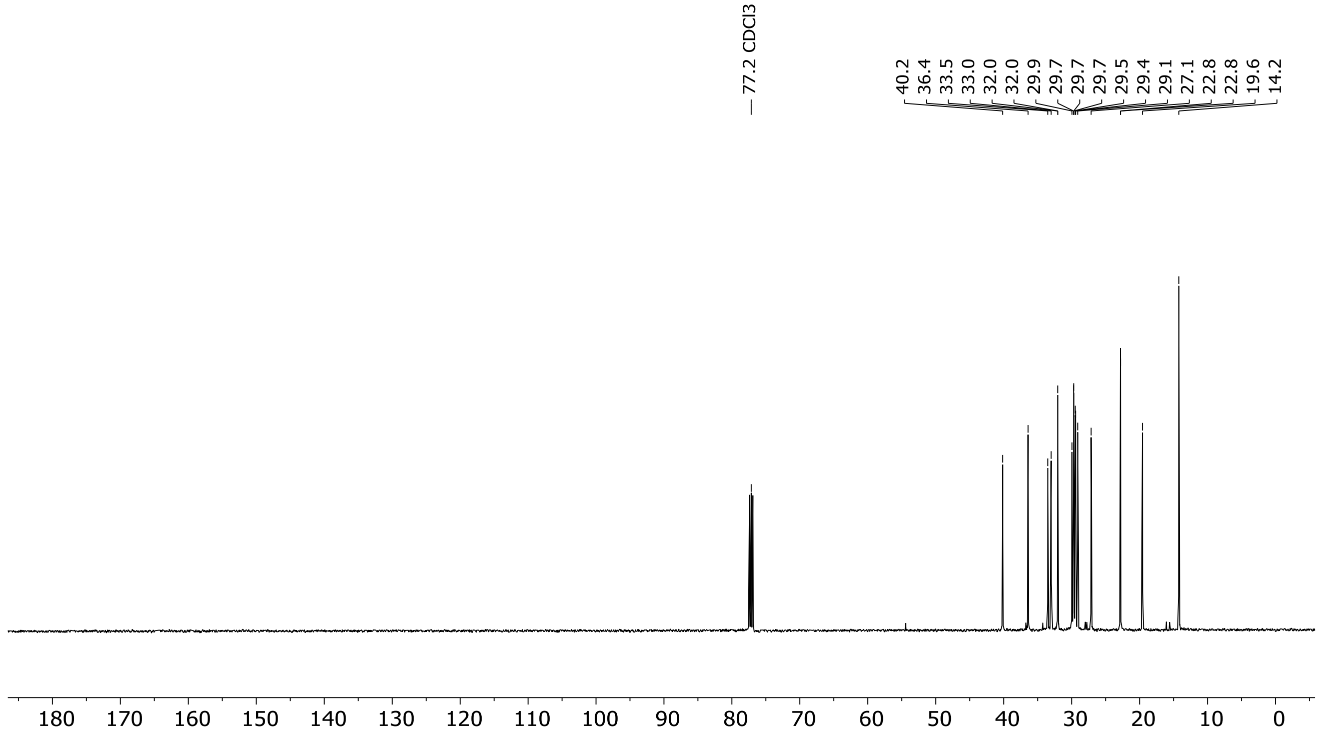

^{13}C{^{1}H} NMR spectrum (125 MHz, 305 K, $CDCl_3$, x-axis in ppm)

1-[(2-Methyloctyl)thio]-2-phenylethane (5p)

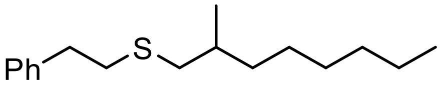

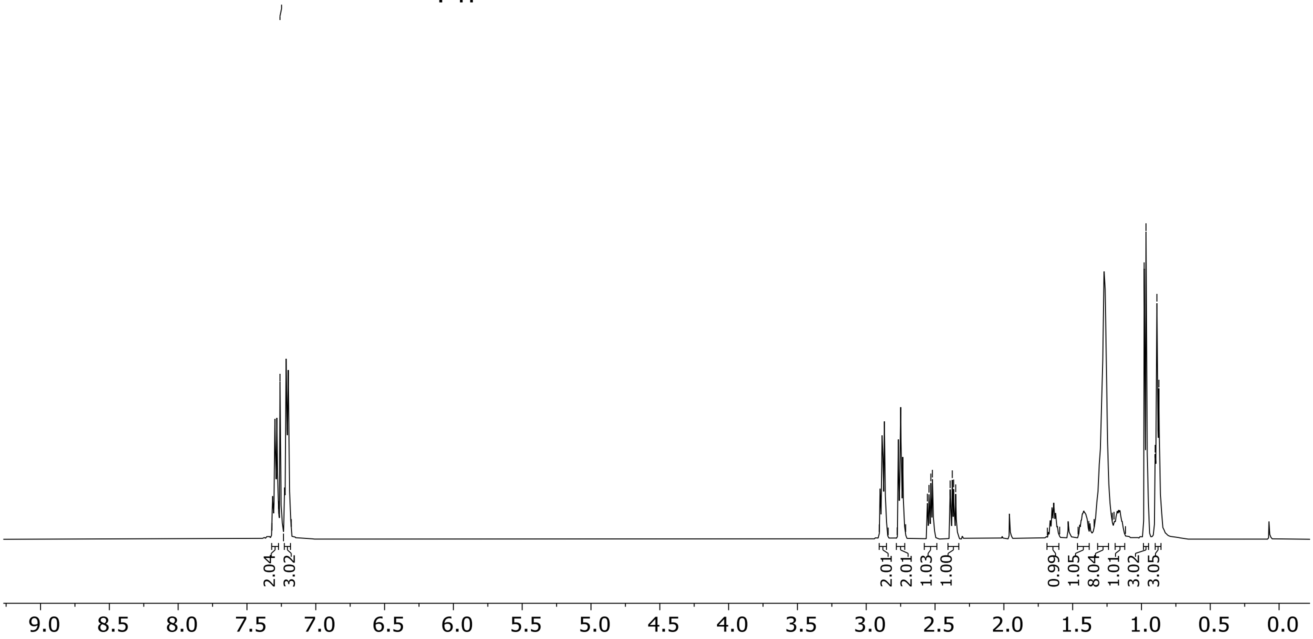

^{1}H NMR spectrum (500 MHz, 305 K, $CDCl_3$, x-axis in ppm)

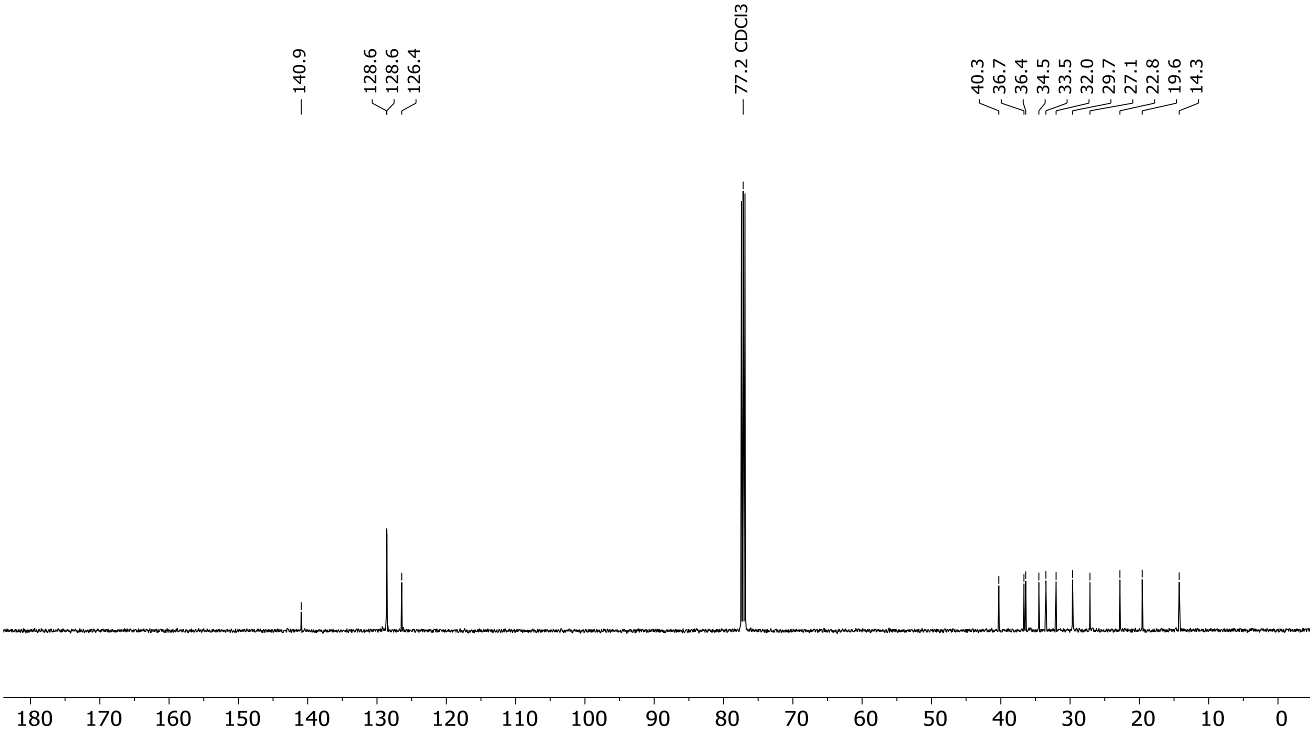

^{13}C{^{1}H} NMR spectrum (125 MHz, 305 K, $CDCl_3$, x-axis in ppm)

(2-Methyloctyl)thioethane (5q)

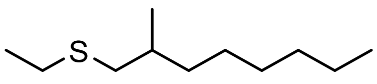

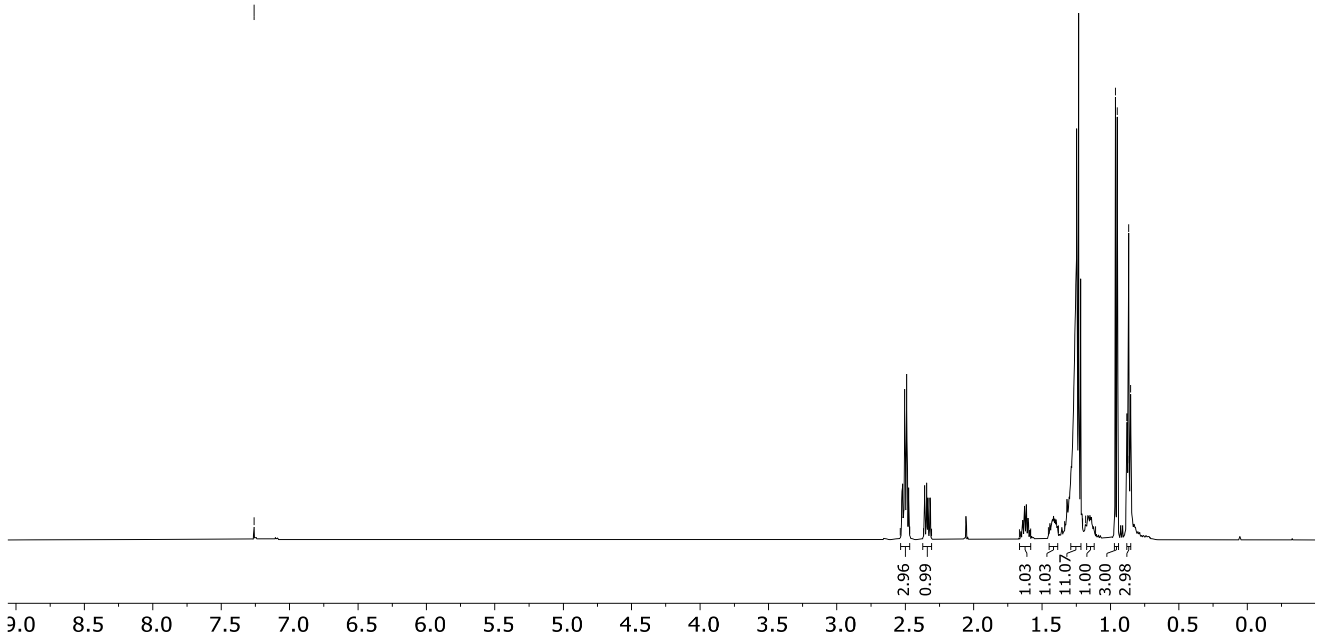

^{1}H NMR spectrum (500 MHz, 305 K, $CDCl_3$, x-axis in ppm)

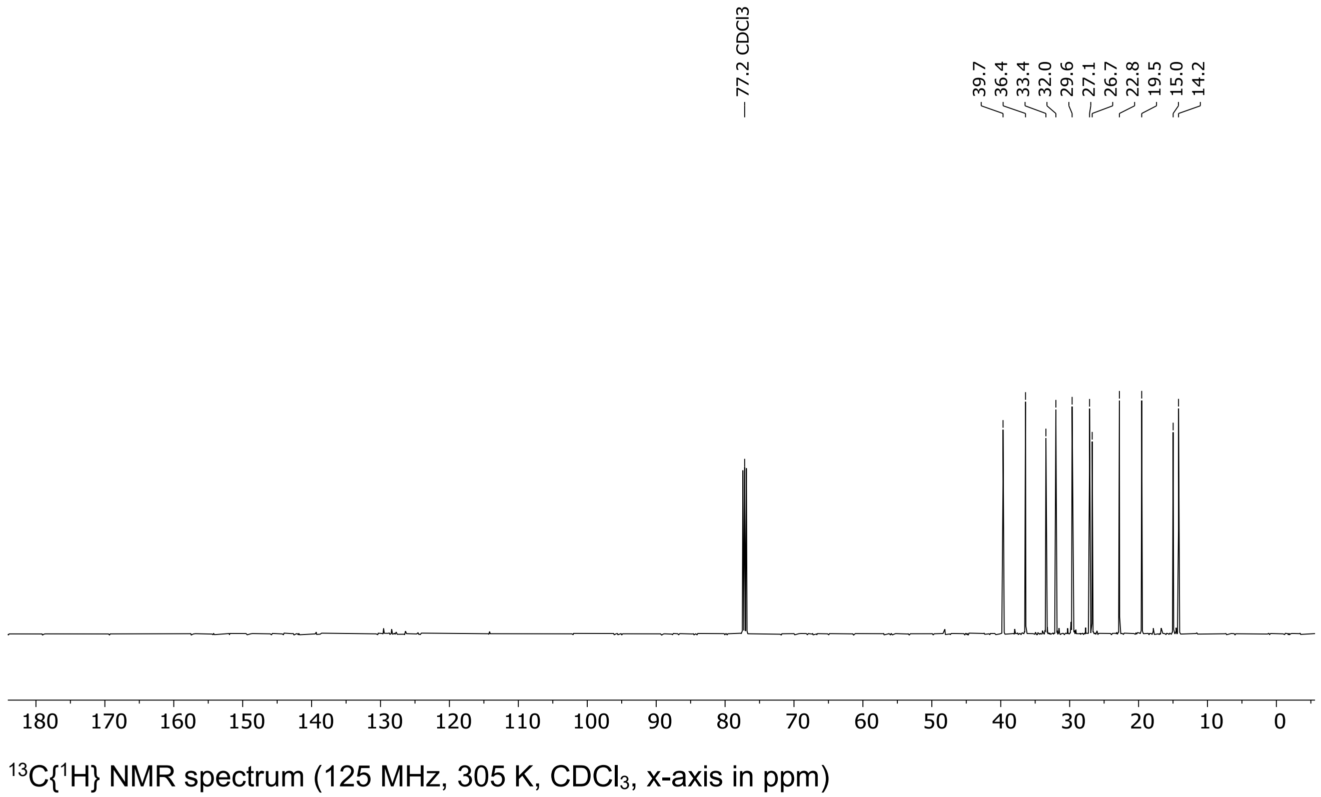

$^{13}C\{^{1}H\}$ NMR spectrum (125 MHz, 305 K, $CDCl_3$, x-axis in ppm)

1-[(2-Methyloctyl)thio]-2-methylpropane (5r)

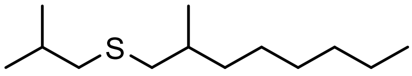

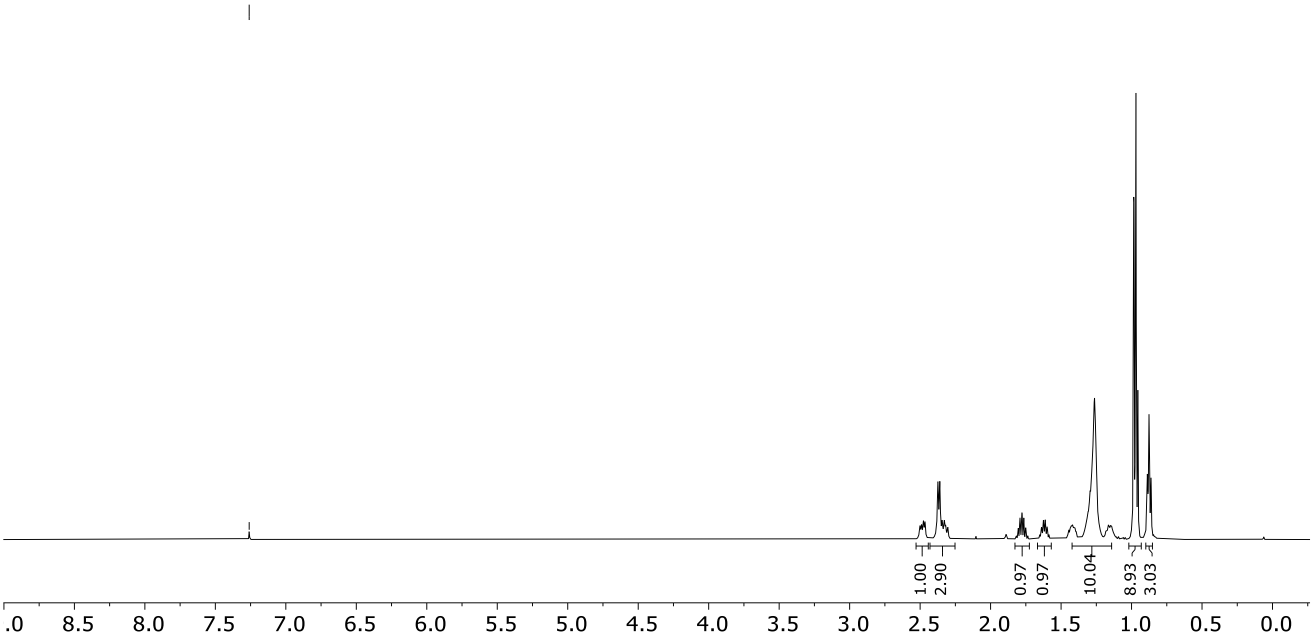

^{1}H NMR spectrum (500 MHz, 305 K, $CDCl_3$, x-axis in ppm)

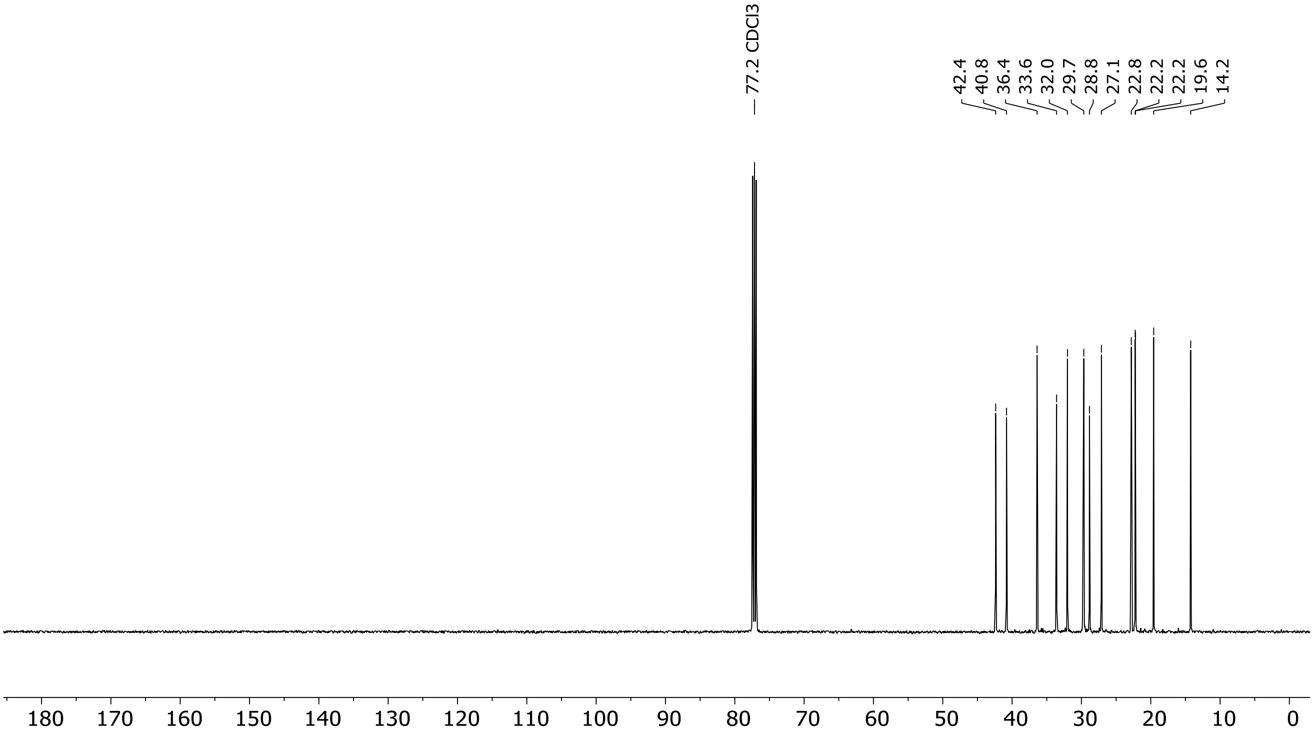

^{13}C{^{1}H} NMR spectrum (125 MHz, 305 K, $CDCl_3$, x-axis in ppm)

2-[(2-Methyloctyl)thio]propane (5s)

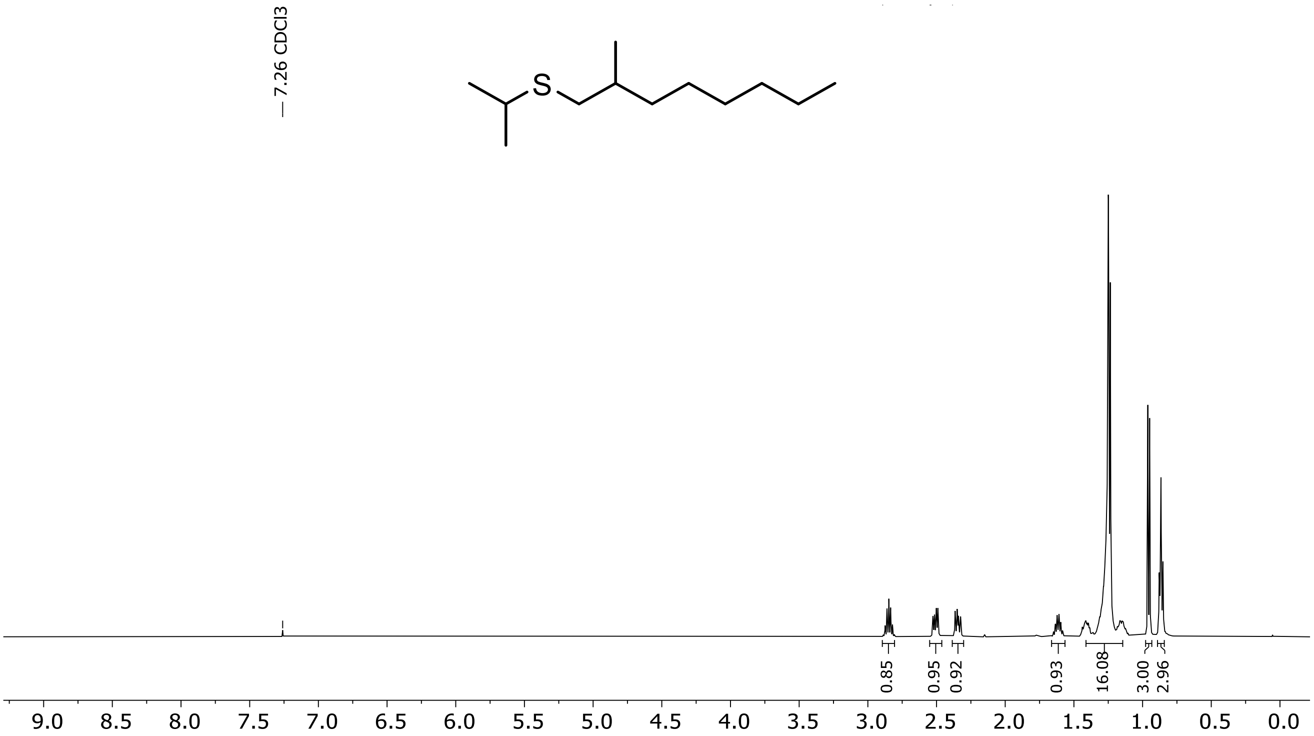

^{1}H NMR spectrum (500 MHz, 305 K, $CDCl_3$, x-axis in ppm)

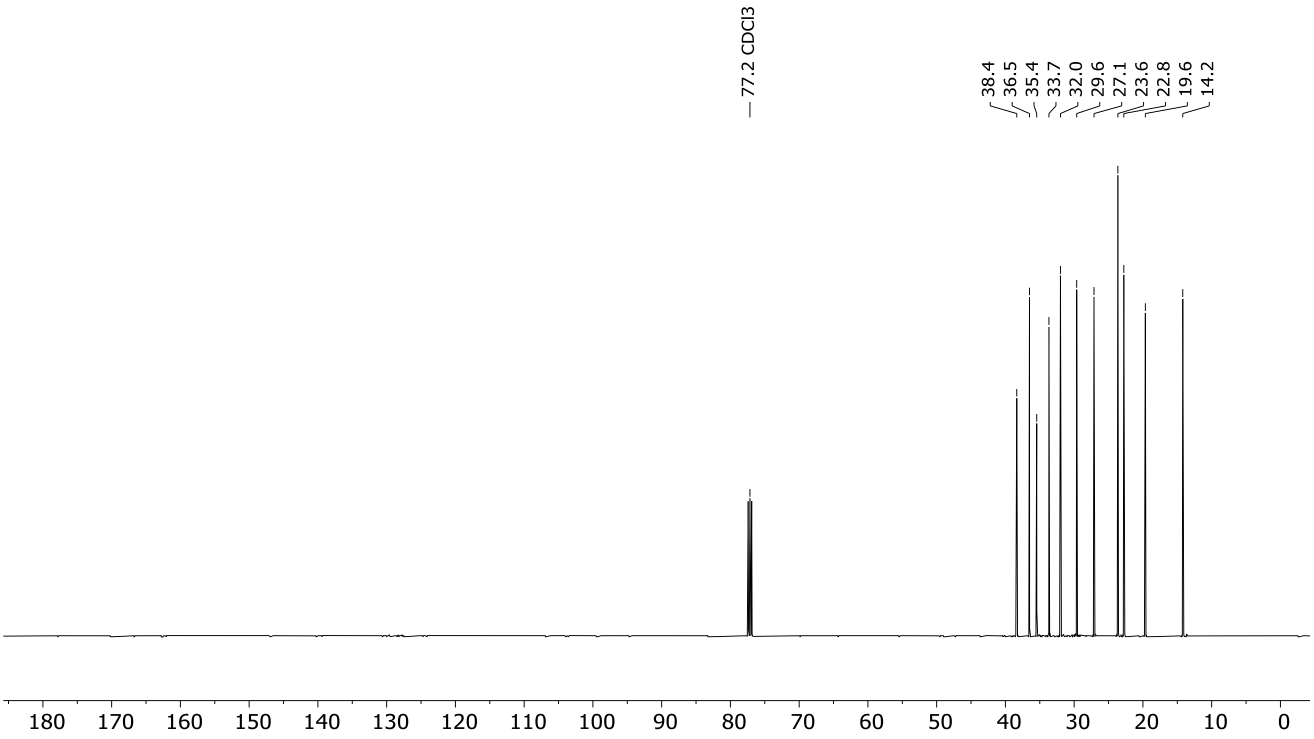

^{13}C{^{1}H} NMR spectrum (125 MHz, 305 K, $CDCl_3$, x-axis in ppm)

(2-Methyloctyl)thiocyclohexane (5t)

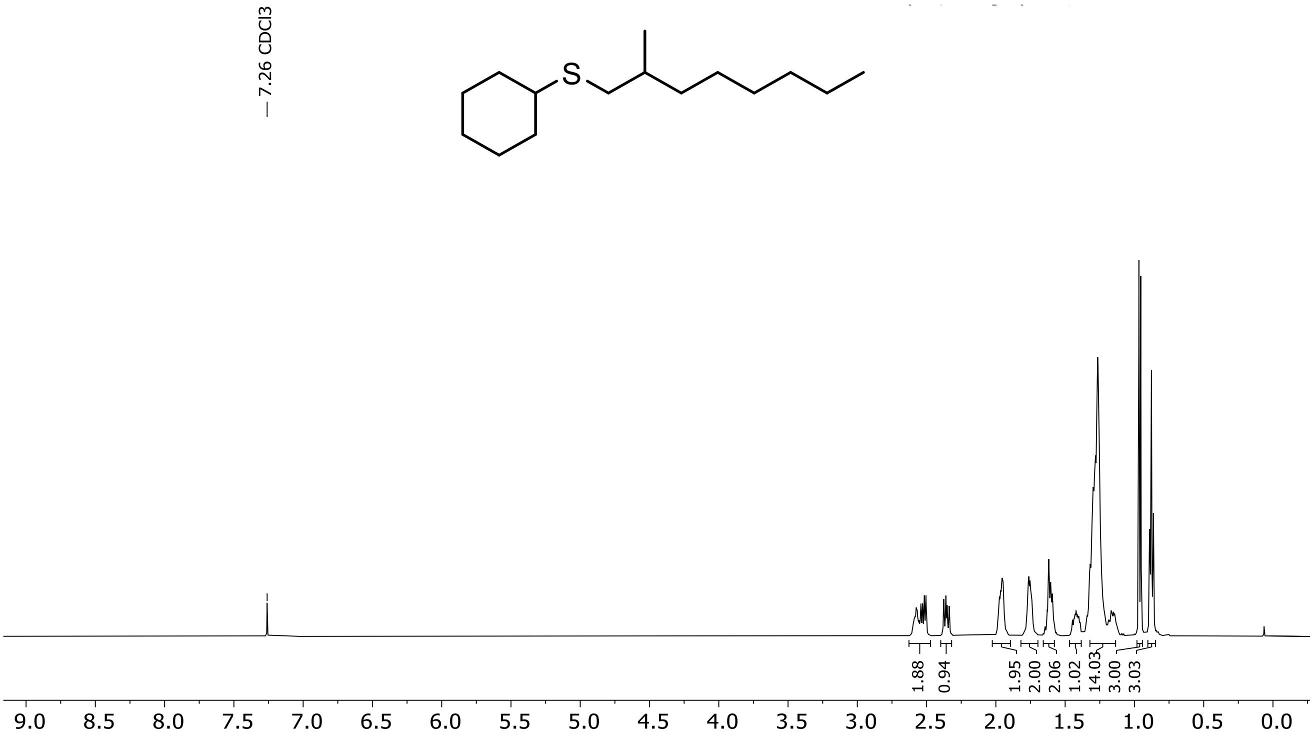

^{1}H NMR spectrum (500 MHz, 305 K, $CDCl_3$, x-axis in ppm)

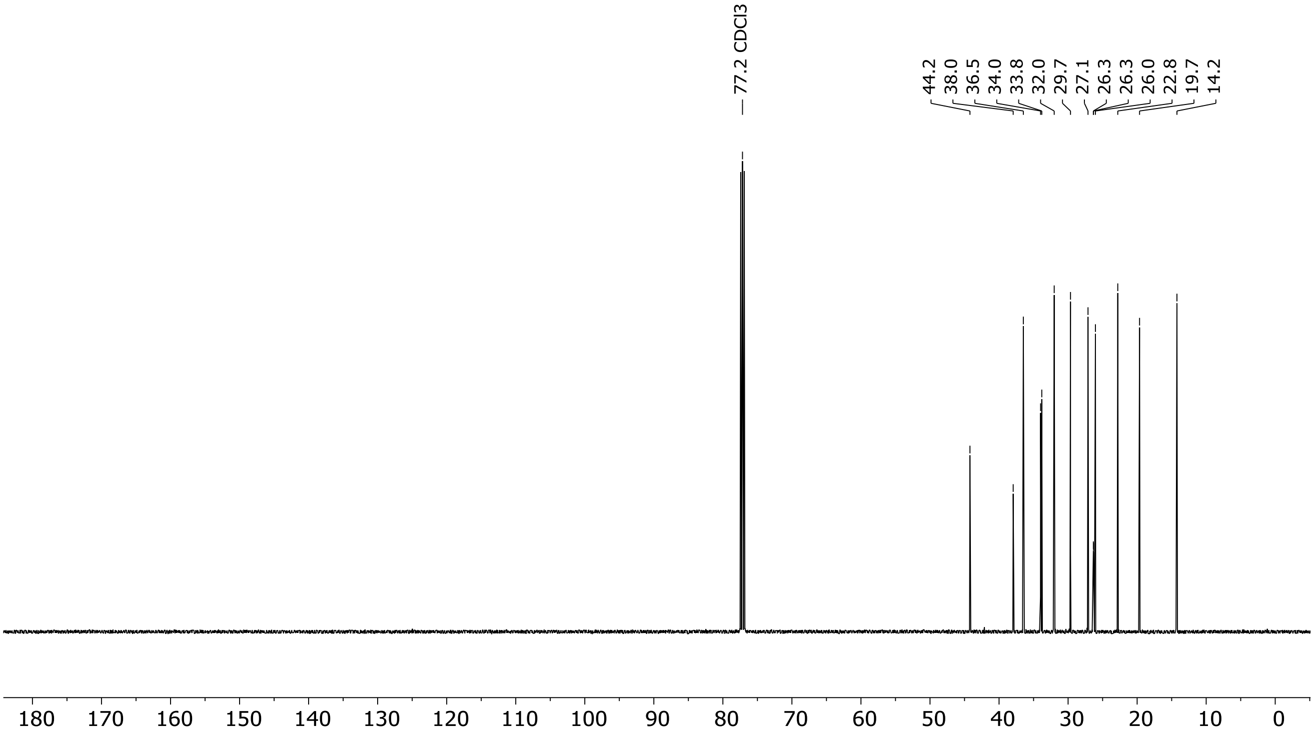

^{13}C{^{1}H} NMR spectrum (125 MHz, 305 K, $CDCl_3$, x-axis in ppm)

(2-Methyloctyl)thiomethane (5u)

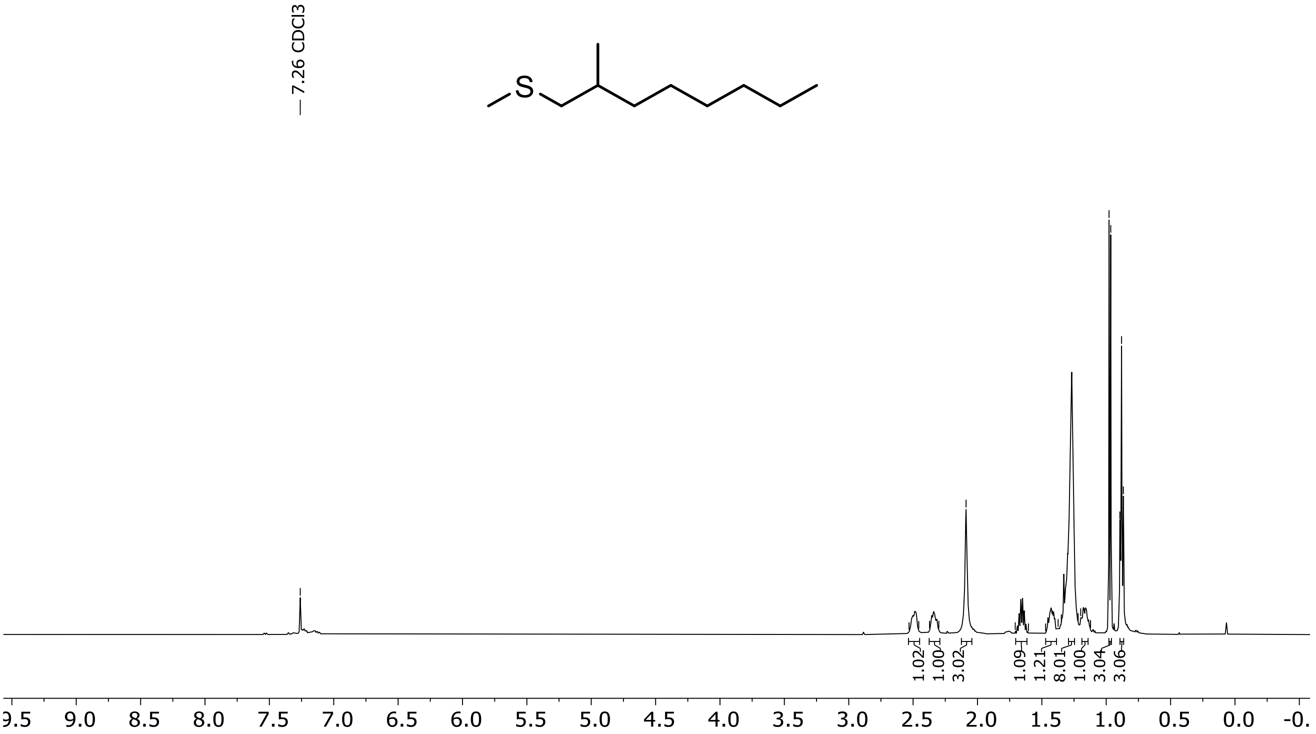

^{1}H NMR spectrum (500 MHz, 305 K, $CDCl_3$, x-axis in ppm)

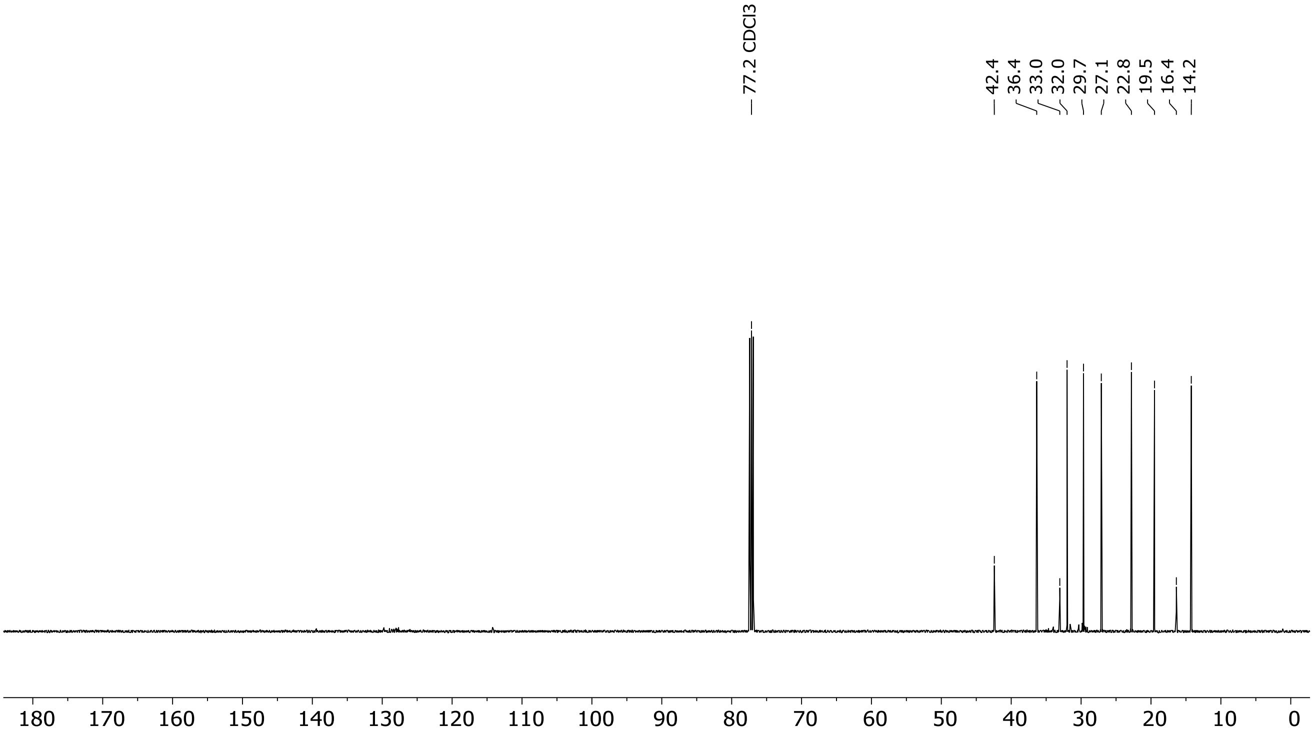

^{13}C{^{1}H} NMR spectrum (125 MHz, 305 K, $CDCl_3$, x-axis in ppm)

Bis(2-methyloctyl)sulfide (5v)

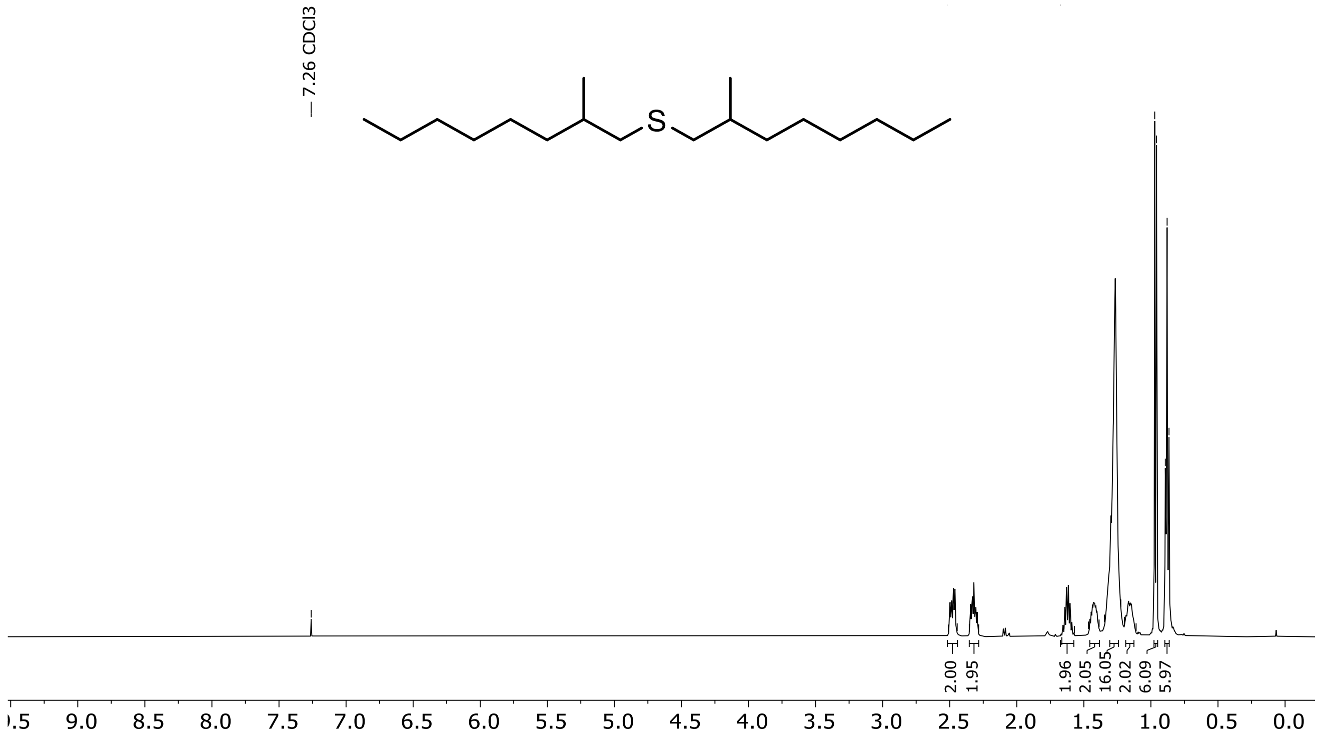

^{1}H NMR spectrum (500 MHz, 305 K, $CDCl_3$, x-axis in ppm)

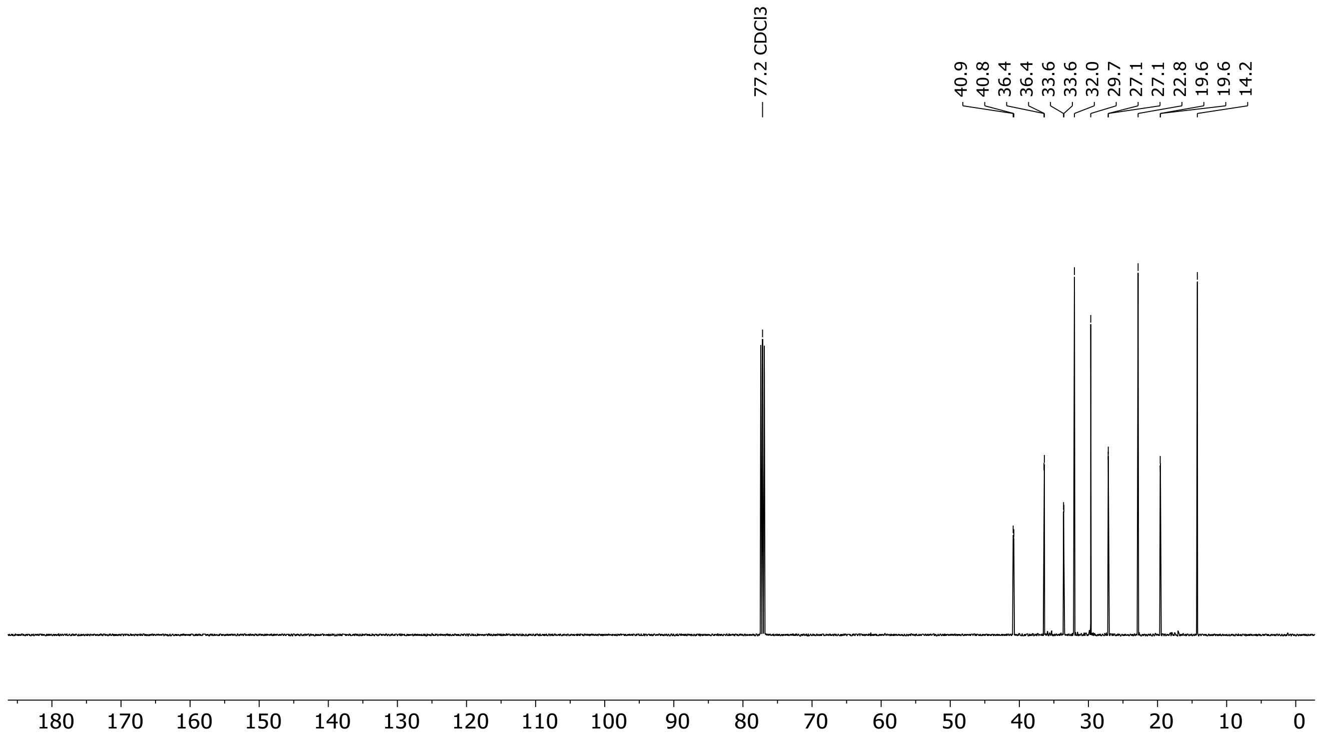

^{13}C{^{1}H} NMR spectrum (125 MHz, 305 K, $CDCl_3$, x-axis in ppm)

[(2-Methyloctyl)thio]methyltrimethylsilan (5w)

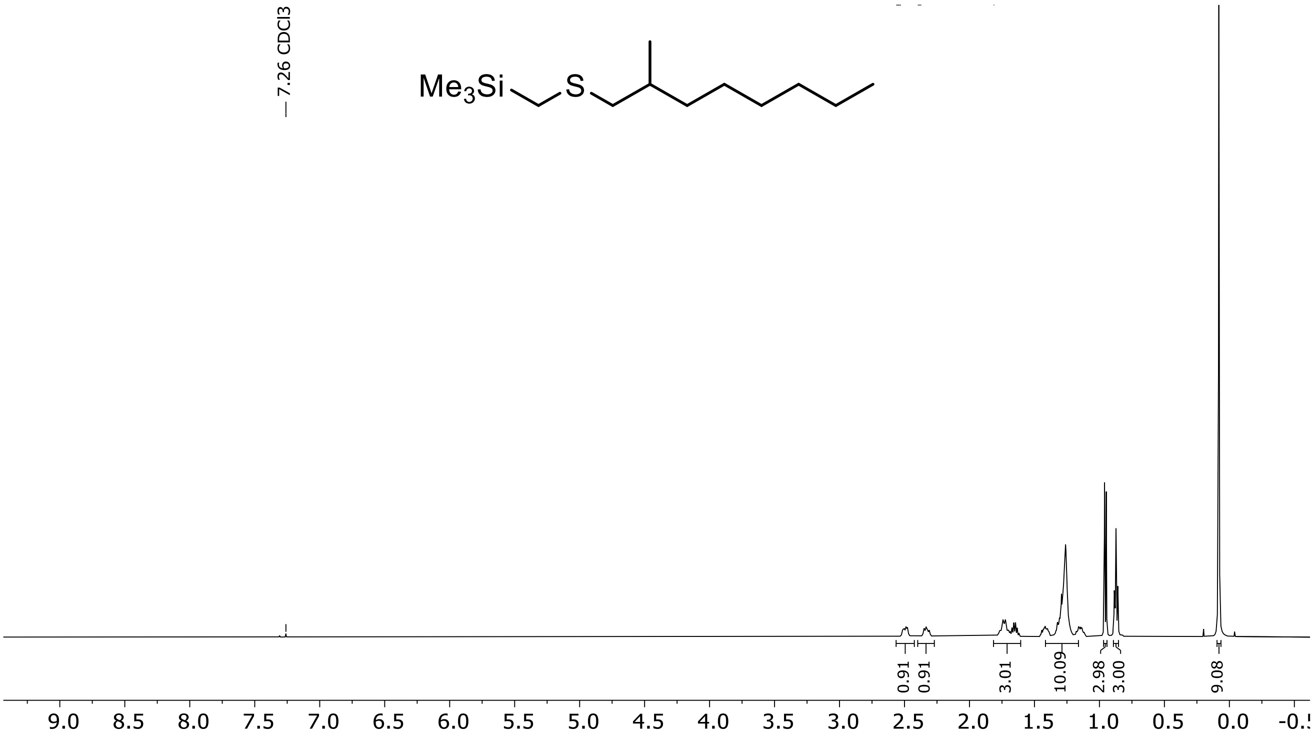

^{1}H NMR spectrum (500 MHz, 305 K, $CDCl_3$, x-axis in ppm)

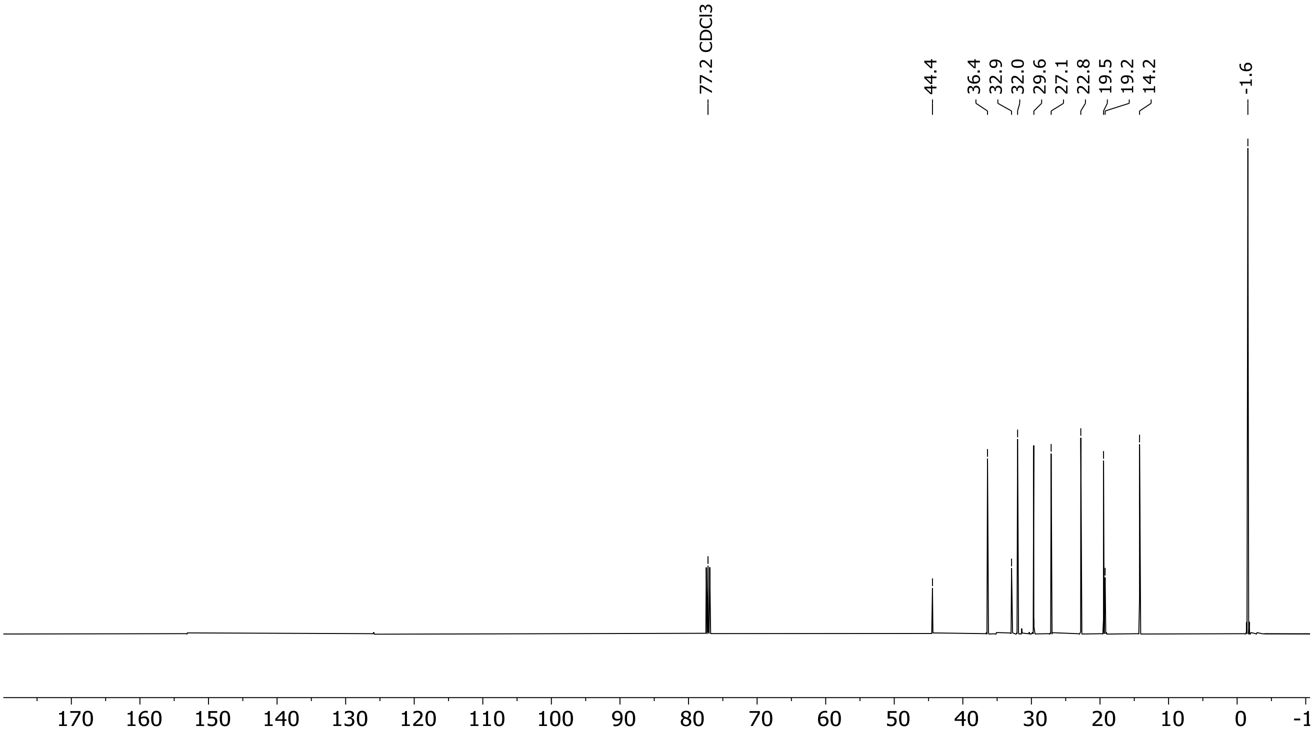

^{13}C{^{1}H} NMR spectrum (125 MHz, 305 K, $CDCl_3$, x-axis in ppm)

1-[(2-Methyloctyl)thio]-2-(4-bromophenyl)ethane (5x)

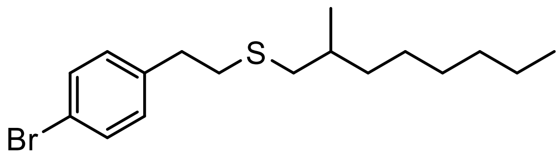

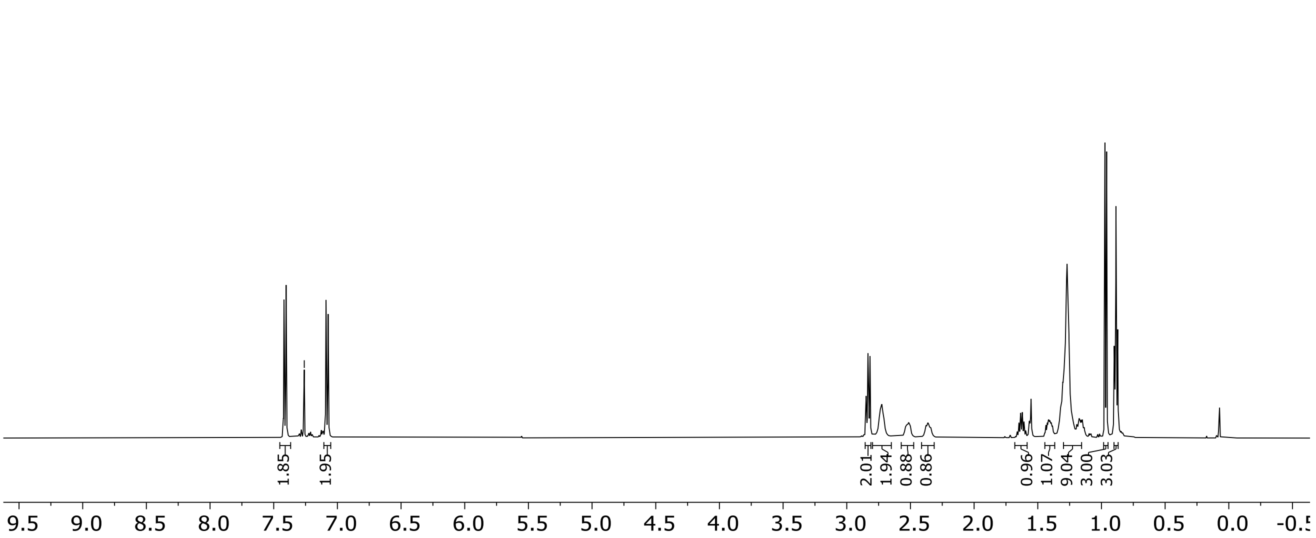

^{1}H NMR spectrum (500 MHz, 305 K, $CDCl_3$, x-axis in ppm)

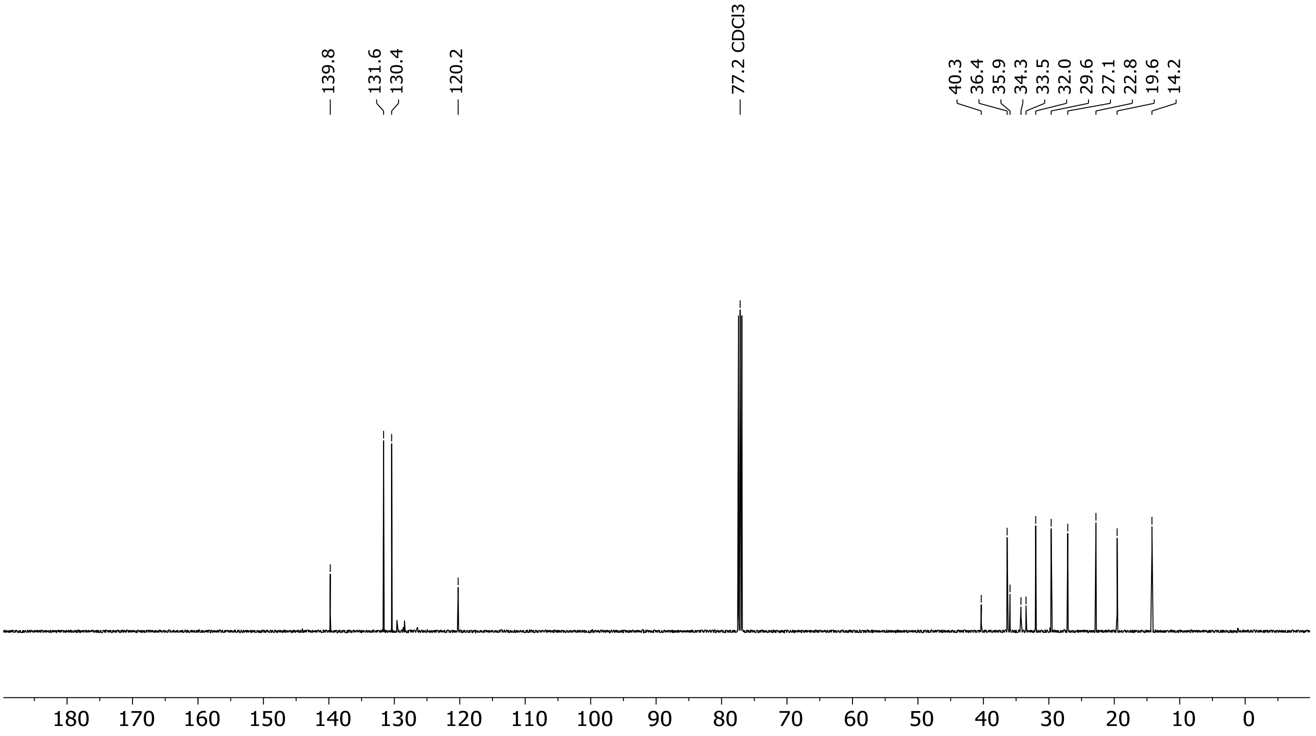

^{13}C{^{1}H} NMR spectrum (125 MHz, 305 K, $CDCl_3$, x-axis in ppm)

Triisopropyl((6-((2-methyloctyl)thio)hexyl)oxy)silane (5y)

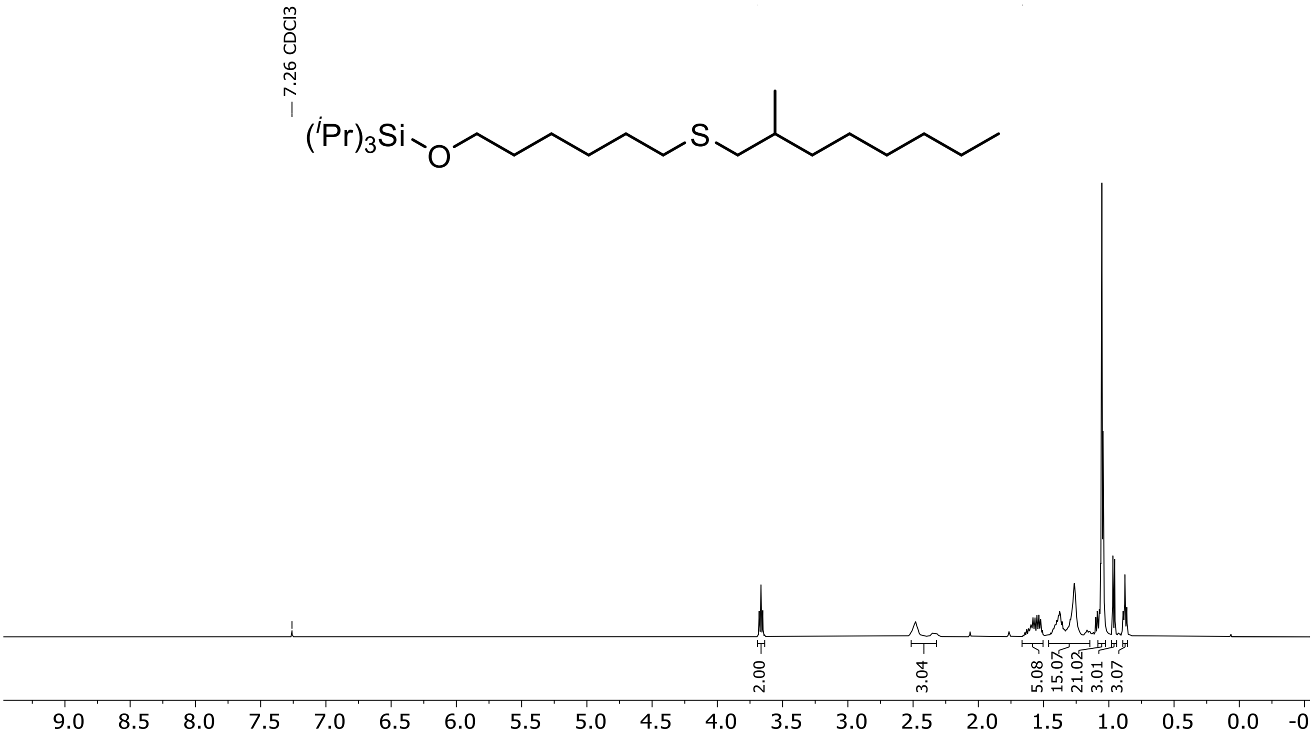

^{1}H NMR spectrum (500 MHz, 305 K, $CDCl_3$, x-axis in ppm)

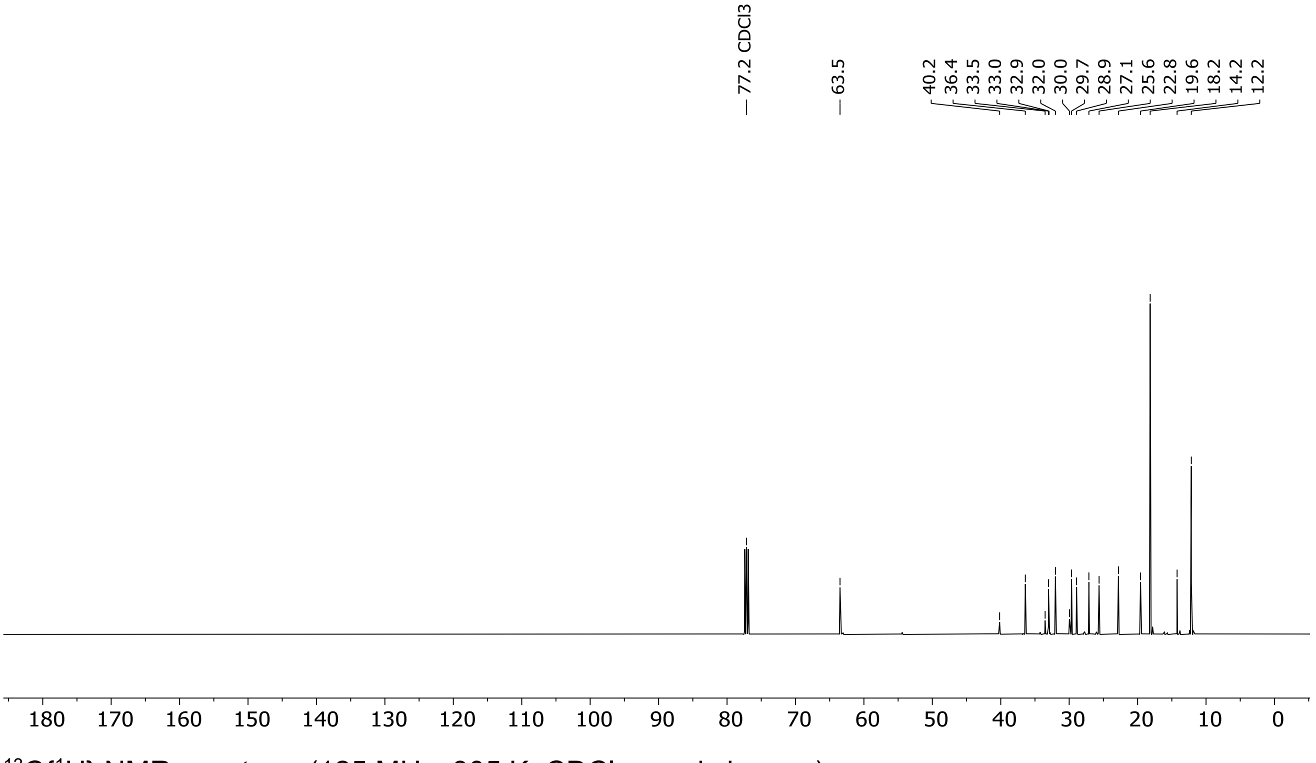

$^{13}C\{^1H\}$ NMR spectrum (125 MHz, 305 K, $CDCl_3$, x-axis in ppm)

2-Methyl-d1-1-(pentylthio)-1,1-d2-octane (*d*-5a)

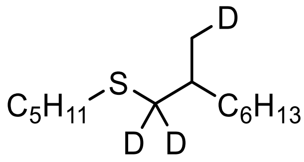

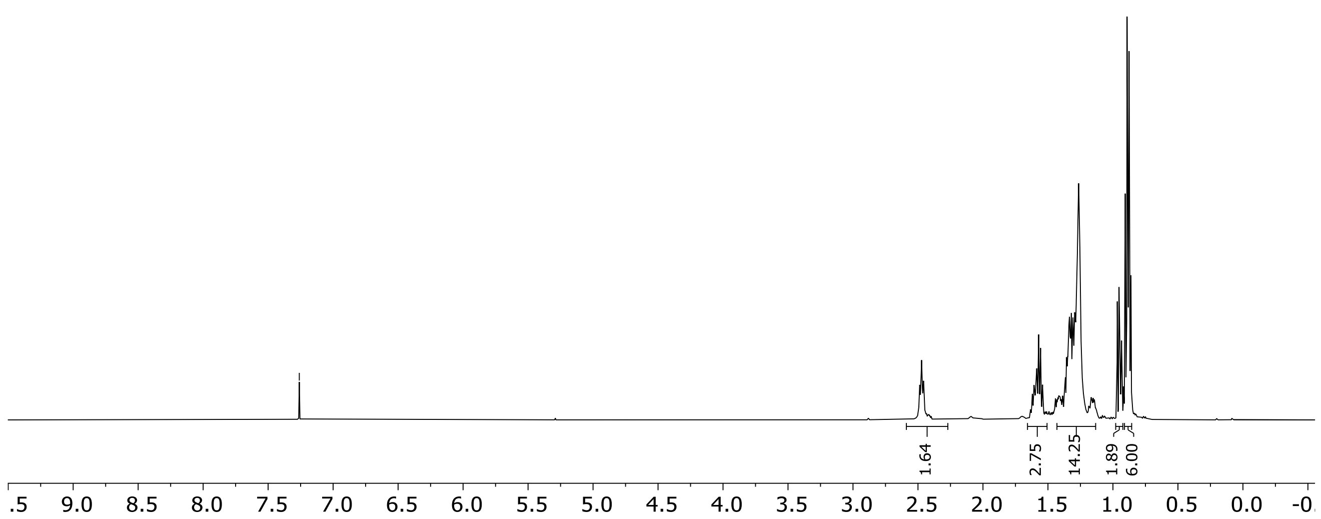

^{1}H NMR spectrum (500 MHz, 305 K, $CDCl_3$, x-axis in ppm)

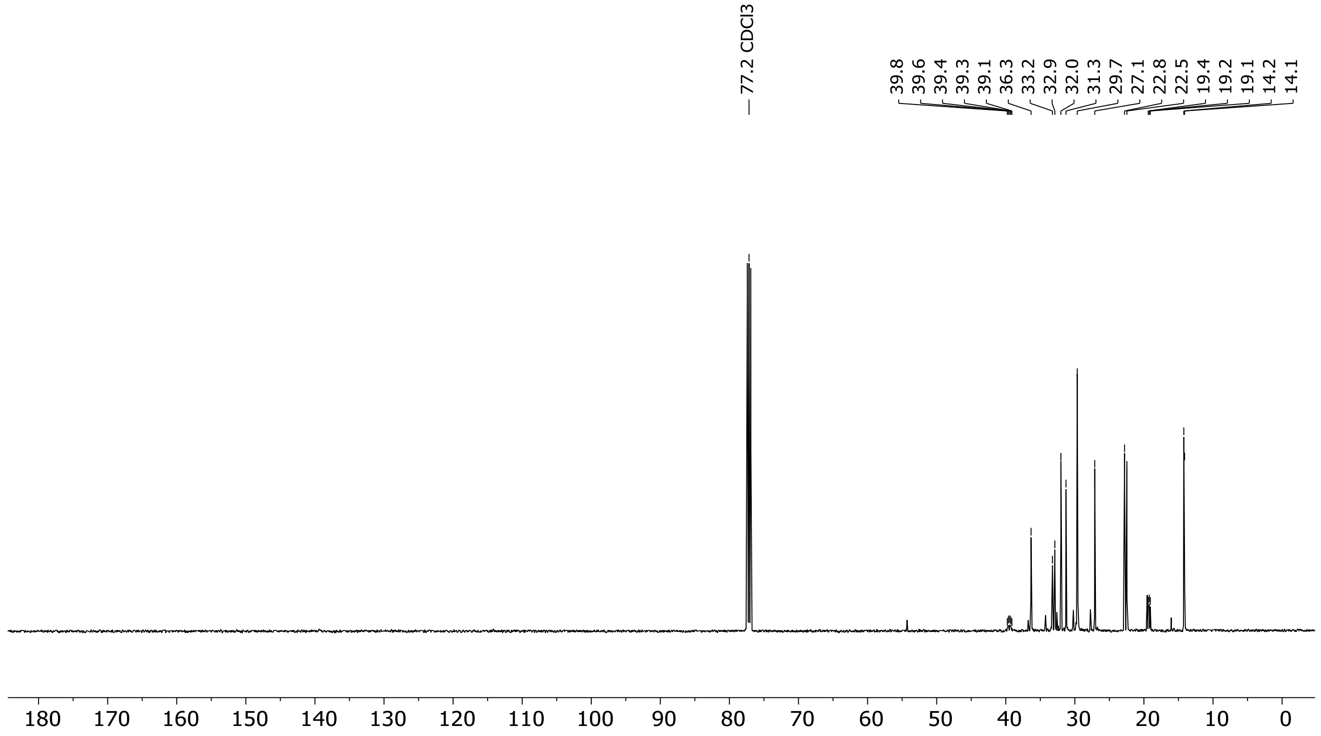

^{13}C{^{1}H} NMR spectrum (125 MHz, 305 K, $CDCl_3$, x-axis in ppm)

7. References

[1] Z. Liu, H. Wu, H. Zhang, F. Wang, X. Liu, S. Dong, X. Hong, X. Feng, *J. Am. Chem. Soc.* **2024**, *146*, 18050-18060.

[2] M. Večeřa J. Gasparič, *Detection and Identification of Organic Compounds*, Springer, Boston, MA, **1971**, pp 317-353.

[3] J. Bielefeld, S. Doye, *Angew. Chem. Int. Ed.* **2017**, *56*, 15155-15158; *Angew. Chem.* **2017**, *129*, 15352-15355.

[4] R. Collier, M. Lappert, R. Pearce, *J. Chem. Soc., Dalton Trans.* **1973**, *65*, 445-450.

[5] Y. Luo, Y. Ma, Z. Hou, *J. Am. Chem. Soc.* **2018**, *140*, 114-117.
